# Piperidine Azasugars Bearing Lipophilic Chains: Stereoselective Synthesis and Biological Activity as Inhibitors of Glucocerebrosidase (GCase)

**DOI:** 10.1021/acs.joc.1c01308

**Published:** 2021-09-01

**Authors:** Francesca Clemente, Camilla Matassini, Sara Giachetti, Andrea Goti, Amelia Morrone, Macarena Martínez-Bailén, Sara Orta, Pedro Merino, Francesca Cardona

**Affiliations:** † Dipartimento di Chimica “Ugo Schiff” (DICUS), University of Firenze, Via Della Lastruccia 3-13, 50019 Sesto Fiorentino (FI), Italy; ‡ Paediatric Neurology Unit and Laboratories, Neuroscience Department, Meyer Children’s Hospital, and Department of Neurosciences, Pharmacology and Child Health, University of Florence, Viale Pieraccini n. 24, 50139 Firenze, Italy; § Departamento de Química Orgánica, Facultad de Química, Universidad de Sevilla, c/ Prof. García González 1, E-41012 Sevilla, Spain; ∥ Unidad de Glicobiología, Instituto de Biocomputación y Física de Sistemas Complejos (BIFI), Universidad de Zaragoza, 50009 Zaragoza, Spain

## Abstract

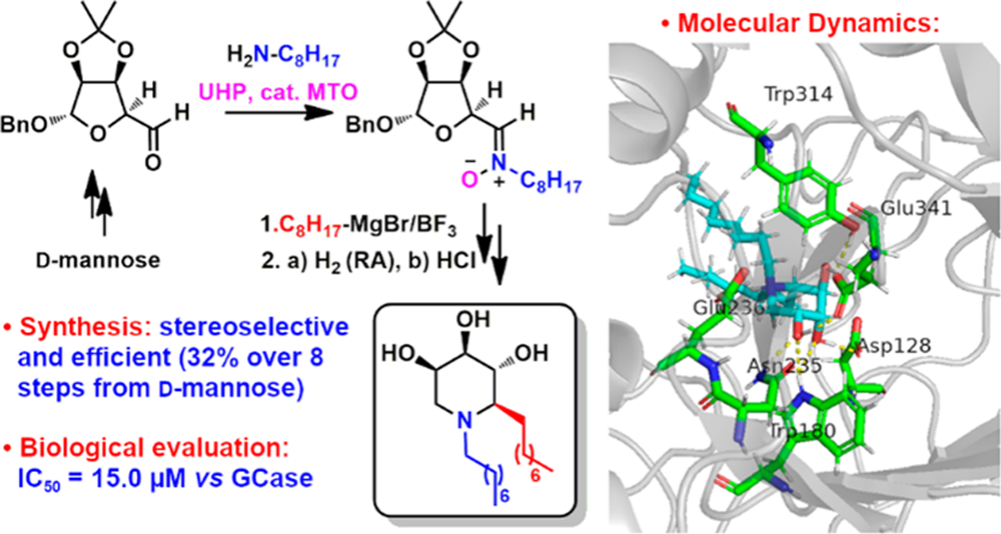

We report a straightforward synthetic strategy for the preparation of trihydroxypiperidine azasugars decorated with lipophilic chains at both the nitrogen and the adjacent carbon as potential inhibitors of the lysosomal enzyme glucocerebrosidase (GCase), which is involved in Gaucher disease. The procedure relies on the preparation of *C*-erythrosyl *N*-alkylated nitrones **10** through reaction of aldehyde **8** and primary amines **13** followed by oxidation of the imines formed *in situ* with the methyltrioxorhenium catalyst and urea hydrogen peroxide. The addition of octylMgBr to nitrone **10e** provided access to both epimeric hydroxylamines **21** and **22** with opposite configuration at the newly created stereocenter in a stereodivergent and completely stereoselective way, depending on the absence or presence of BF_3_·Et_2_O. Final reductive amination and acetonide deprotection provided compounds **14** and **15** from low-cost d-mannose in remarkable 43 and 32% overall yields, respectively, over eight steps. The C-2 *R*-configured bis-alkylated trihydroxypiperidine **15** was the best ligand for GCase (IC_50_ = 15 μM), in agreement with MD simulations that allowed us to identify the chair conformation corresponding to the best binding affinity.

## 1 Introduction

**Figure 1:**
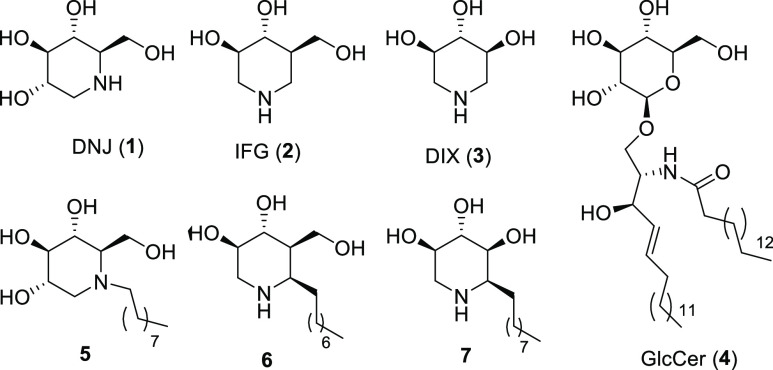
Some imino- and azasugars, their *N*- and *C*-alkyl derivatives, and glucosylceramide (GlcCer), the natural substrate of GCase.

Iminosugars [*e.g.*, deoxynojirimycin, DNJ (**1**), Figure 1] are among the most fascinating monosaccharide analogues in which a nitrogen atom replaces the endocyclic oxygen.^1^ Together with azasugars, [e.g. isofagomine, IFG (**2**), or 1,5-dideoxy-1,5-iminoxylitol, DIX (**3**), Figure 1], which are characterized by a nitrogen atom replacing the anomeric carbon of monosaccharides, iminosugars have been extensively investigated in the last thirty years as glycosidase^2^ and glycosyltransferase inhibitors.^3^

More recently, imino- and azasugar-based glycomimetics became attractive as potential therapeutic agents toward lysosomal storage disorders (LSDs), following the observation of their counter-intuitive effect in enhancing the enzyme activity, thus acting as chaperones. In the pharmacological chaperone therapy (PTC) of LSDs, these glycomimetics are employed at sub-inhibitory concentration to favor the mutated enzyme correct folding in the endoplasmic reticulum (ER), facilitate its translocation to the lysosomes, and recover some hydrolytic activity, compromised as a consequence of diverse genetic mutations.^4^

Gaucher disease (GD), the most common LSD, is determined by mutations in the *GBA* gene, which encodes for the lysosomal enzyme glucocerebrosidase (GCase). The *GBA* mutations provoke partial deficit of GCase, with consequent loss or reduction of its hydrolytic activity [*i.e.*, hydrolysis of glucosyl ceramide, GlcCer (**4**), Figure 1, to ceramide and glucose] in the lysosomes. Accumulation of glucosylceramide then leads to organ dysfunctions and severe impairment. Several good candidates behaving as pharmacological chaperones (PC) for GCase have been found, but no drugs are on the market yet.^5^

The presence of an alkyl chain either at the nitrogen atom or at the adjacent carbon of imino- and azasugars was found to improve their pharmacokinetic properties, furnishing better PCs for GD. For instance, contrary to DNJ (**1**), *N*-nonyl-DNJ (**5**) (Figure 1) is a potent inhibitor of lysosomal GCase (IC_50_ = 1 μM) which showed a twofold increase in the activity of the N370S mutant enzyme in fibroblasts at 10 μM concentration. However, it did not enhance the intracellular activity of the L444P variant.^6^

With IFG and DIX derivatives, better results in terms of PC properties were obtained by shifting the alkyl chain from nitrogen to the adjacent carbon. Indeed, 6-nonyl IFG (**6**) (Figure 1) displayed a remarkable GCase inhibition and PC activity (IC_50_ = 0.6 nM, 1.5-fold enzyme activity enhancement at 3 nM in N370S GD fibroblasts)^7^ and α-1-*C*-nonyl-DIX (**7**) showed an IC_50_ of 6.8 nM toward GCase and a 1.8-fold enzyme activity increase in N370S mutated fibroblasts at 10 nM.^8^

**Scheme 1:**
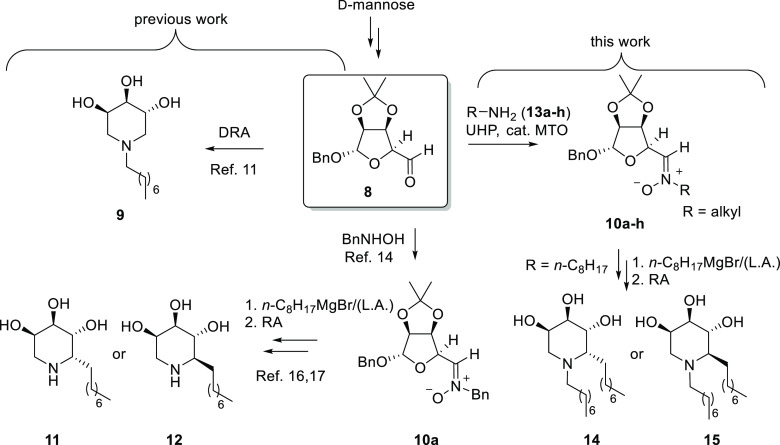
*Previous rResults and Scope of This Work* General strategies employed to access *C*- or *N*-alkyl trihydroxipiperidines from the “masked dialdehyde” **8** and nitrone **10a** derived thereof. *This work*: “one-pot” synthesis of *C*-erythrosyl *N*-alkyl nitrones **10** and their application to the synthesis of *C*,*N*-dialkylated azasugars **14** and **15**.

Among the most straightforward synthetic strategies to afford polyhydroxylated piperidine imino- and azasugars^9^ are the reductive amination (RA) ring-closure starting from nitrogen-containing carbohydrate-based precursors or the double reductive amination (DRA) of dicarbonyl carbohydrate derivatives with an external nitrogen source.^10^ By applying the latter strategy on the “masked” dialdehyde **8** derived from d-mannose, we have synthesized the *N*-alkyl trihydroxypiperidine **9** (Scheme 1),^11^ which showed IC_50_ = 30 μM toward GCase and 1.25-fold activity increase in N370S-mutated fibroblasts at 100 μM.^12^

The former RA strategy, instead, was applied to the *C*-erythrosyl nitrone **10a**,^13^ obtained in turn by condensation of the same “masked” dialdehyde **8** with *N*-benzyl hydroxylamine.^14^ Stereoselective Grignard additions to this key nitrone in the presence or absence of a suitable Lewis acid^14,15^ afforded, in a stereodivergent manner, both epimers of a series of 2-alkylated trihydroxypiperidines, among which the octyl derivatives **11** and **12** (Scheme 1) showed the most promising biological properties.^16^ In particular, the (2*R*) diastereoisomer **12** showed remarkable PC properties toward fibroblasts bearing the N370S/RecNcil mutation (1.9-fold enzyme recovery at 100 μM) and, more importantly, proved to be responsive with the homozygous L444P mutation (1.80-fold enzyme recovery at 100 μM), which is refractory to most PCs. Remarkably, both compounds **9** and **12** performed PC tasks toward wild-type fibroblasts (1.5-fold at 50 μM and 1.4-fold at 100 μM, respectively), which is an important factor for targeting sporadic forms of Parkinson disease.^17^

Given the relevance of the presence of a long alkyl chain for inducing good enzyme recognition, and in consideration of the structure of the natural substrate GlcCer (**4**), we speculated that compounds possessing two alkyl chains might show improved biological properties.

We therefore addressed the synthesis of 1,2-dialkyltrihydroxypiperidines and envisaged that a general synthetic strategy could involve the Grignard addition to *C*-erythrosyl *N*-alkyl nitrones **10** followed by RA (Scheme 1).

In this work, we report our results on this subject, which include a docking study of the putative 1,2-dialkyltrihydroxypiperidines in the GCase catalytic site; a general direct synthesis of nitrones **10** and their conversion to target compounds **14** and **15** and to derivatives containing three alkyl tails; the biological evaluation of the dialkylated trihydroxypiperidines toward commercial glycosidases and human lysosomal enzymes and their *in vitro* activity on cell lines; a molecular dynamics (MD) simulation of the new compounds within the GCase enzyme active cavity.

## 2 Results and Discussion

### 2.1 Preliminary Docking Studies

**Figure 2:**
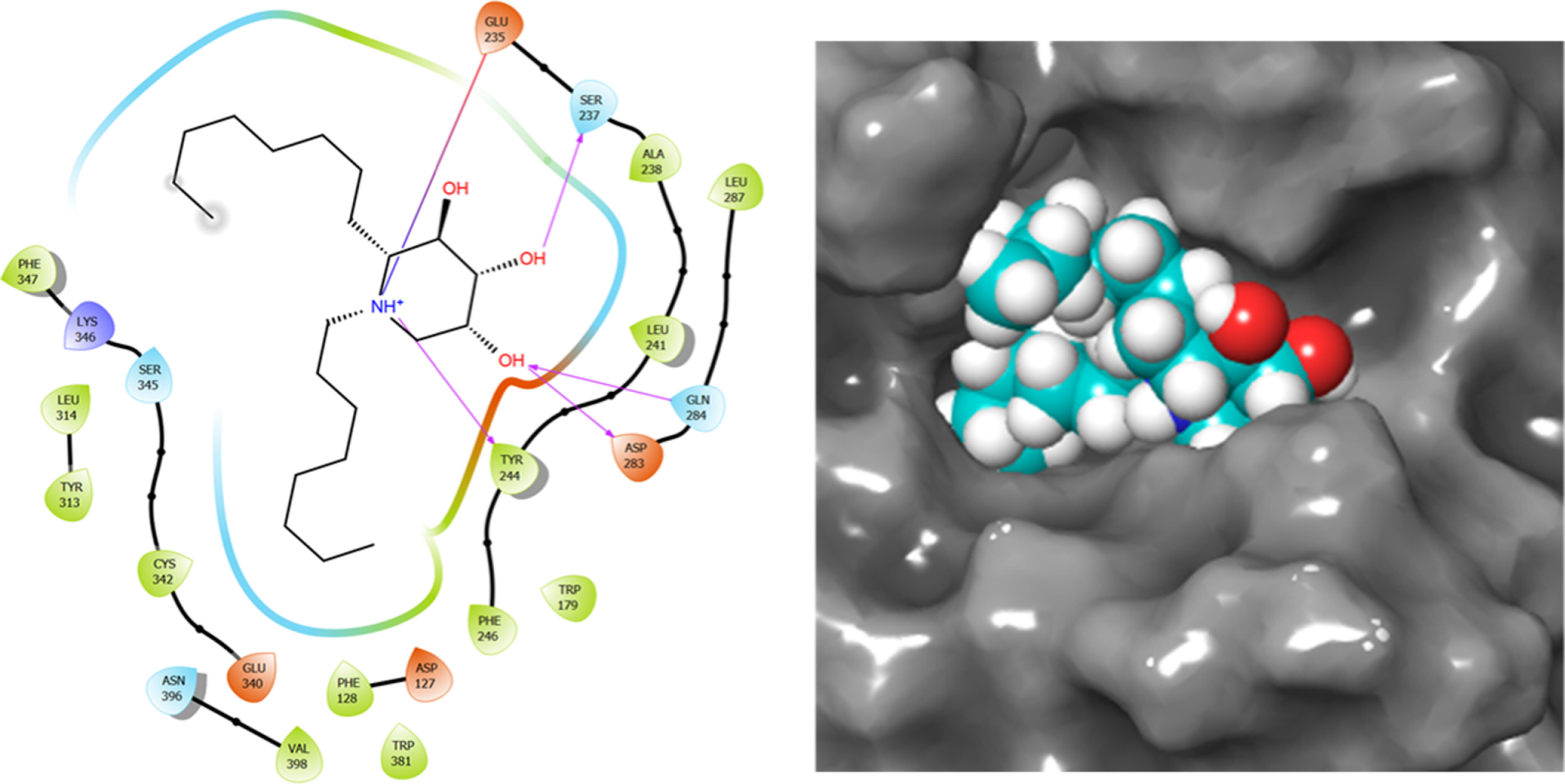
Molecular docking for protonated **15** with an (S)-configuration at the nitrogen atom. Left: 2D schematic view of interaction in the binding site. Right: Visualization of the ligand, represented as cyan CPK at the binding site.

With the aim of assessing the viability of target compounds **14** and **15** in GCase catalytic site, we carried out preliminary docking studies with acid-beta-glucosidase (PDB ID 2NSX).^18^ The protonated forms of compounds **14** and **15** were considered, taking into account that a stereogenic nitrogen atom is formed (for details, see the Supporting Information section). For compound **14**, the best pose corresponds to an (*R*)-configuration at the nitrogen atom adopting a ^1^C_4_ conformation (score −6.332). The observed orientation for the piperidine ring is different with respect to that observed in a known chaperone [5-hydroxymethyl-3,4-dihydroxypiperidine, IFG (**2**), Figure 1], thus loosing interactions of hydroxyl groups with Asp127, Trp179, and Asn396 that now form new interactions with Ser237, Asp283, and Gln284. Although the aliphatic chain linked to the nitrogen atom shows hydrophobic interactions with Phe128, Trp179, Tyr313, Leu314, and Ala238, the other aliphatic chain is clearly exposed to the solvent (see the Supporting Information section). The (*S*)-isomer shows a close value in the docking score (−6.071) for the same conformation and, also in this case, the piperidine ring is oriented in the wrong way with respect to IFG. A similar situation was found for protonated **15**. In this case, the (*S*)-isomer in a ^1^C_4_ conformation showed the best docking value (−6.614) due to hydrophobic interactions of the aliphatic chains in the binding site, the (*R*)-isomer in the same conformation being very close (docking score = −6.354). Again, in both cases, the piperidine ring is oriented in the wrong way when compared with the original structure bound to IFG. For the (*R*)-isomer, the chain bonded to the nitrogen atom gives interactions with Phe128, Trp179, Phe246, and Trp381. The similar orientation of the piperidine ring to that observed for **14** gives rise to the same interactions of the hydroxyl groups with Ser237, Asp283, and Gln284 (Figure 2). These preliminary results should be considered with caution due to the predicted orientations of the piperidine ring in a rigid protein. Even though the calculations predict a good binding to the enzyme, the particular interactions and orientations of the ligands will be confirmed (or rejected) through further molecular dynamics studies that will allow conformational changes in the protein (vide infra).

### 2.2 Synthesis and Structural Assignment

The aldehyde **8** was synthesized in four steps from d-mannose on gram scale as reported.^11,19^

The synthesis of nitrones **10** from **8** by condensation in analogy to the reported **10a** would require the corresponding *N*-monosubstituted hydroxylamines, compounds not readily available in general. However, nitrones can also be obtained in several alternative ways,^20^ among which the oxidation of imines developed by some of us^21^ was selected as the most appropriate for our purposes. This procedure was established first on preformed imines employing methyltrioxorhenium (MTO) as the catalyst and urea hydrogen peroxide complex (UHP) as a mild stoichiometric oxidant. The methodology has the great advantage over other oxidation methods (on secondary amines^22^ or *N*,*N*-disubstituted hydroxylamines)^20a^ of forming a single nitrone, since the double bond is previously installed during the imine formation. Moreover, a convenient one-pot version has been subsequently implemented, where imines are formed *in situ* from inexpensive or readily available primary amines and aldehydes.^23^

In this work, we employ the one-pot condensation/oxidation strategy to our carbohydrate-derived “masked” dialdehyde **8** with primary amines **13a–h** for the synthesis of several new *C*-carbohydrate *N*-alkyl nitrones **10** (Scheme 1). We also demonstrate that such nitrones undergo stereodivergent and highly stereoselective Grignard reagent additions in the presence or absence of a suitable Lewis acid. The following reductive amination (RA) of the formed adducts directly gives access to new *C,N*-dialkyl trihydroxypiperidines, as demonstrated with the synthesis of compounds **14** and **15** (Scheme 1). The target compounds were designed as to better mimic the two chains of the GlcCer (**4**) natural substrate.

The one-pot condensation/oxidation was investigated starting from aldehyde **8** with structurally diversified primary amines **13a–h** followed by addition of UHP and catalytic MTO, and the results are shown in Table 1. The reaction afforded the corresponding *N*-alkyl nitrones **10a–h** with good yields and in an operationally very simple manner. The procedure was optimized using benzylamine (**13a**) for the preparation of the known nitrone **10a**. The best results were obtained by stirring a solution of the aldehyde **8** with 1.2 equiv of the appropriate amine **13** in MeOH at room temperature in the presence of anhydrous Na_2_SO_4_, until disappearance of the starting aldehyde **8** at an ^1^H NMR control (3–5 h, depending on the amine), which attested the formation of the corresponding imine. After cooling of the reaction mixture at 0 °C, addition of UHP (3 equiv) and MTO (4 mol %) caused the solution to turn yellow, indicating the formation of the catalytically active peroxorhenium species.^24^ Upon completion of the oxidation at room temperature, a simple work-up consisting of solvent removal under reduced pressure followed by CH_2_Cl_2_ addition allowed filtering off the undissolved urea and Na_2_SO_4_ to give, after evaporation of the solvent, the crude nitrones which were purified by flash column chromatography on silica gel. Alternative addition of UHP and MTO since the beginning of the reaction (prior to imine formation) was found to be unpractical and afforded complex mixtures of products deriving from competitive oxidation of the primary amines.

**Table 1:**
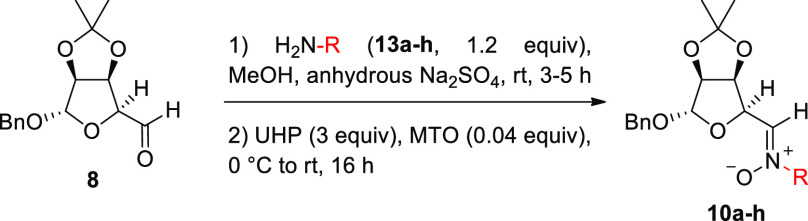

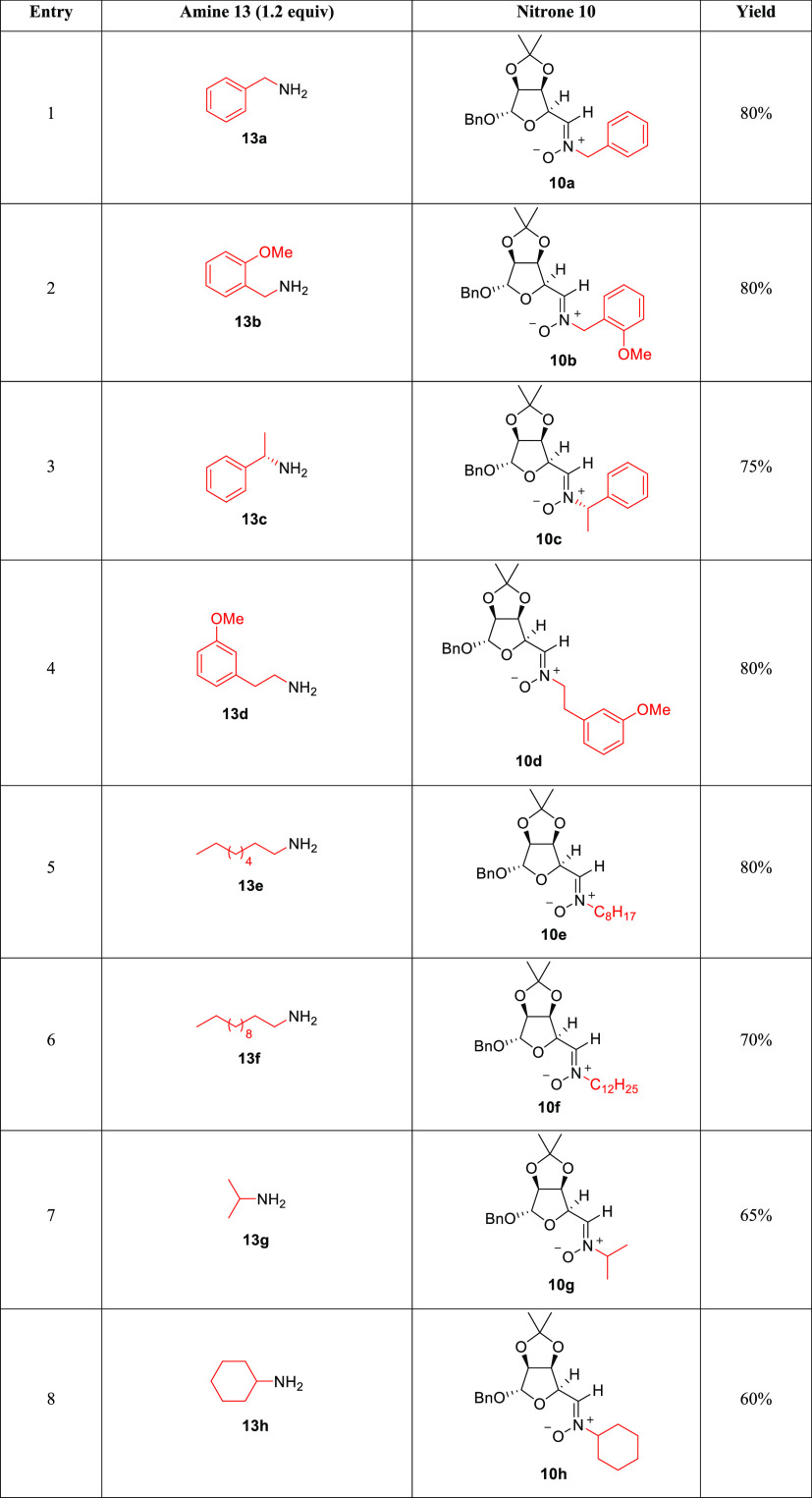
Condensation/Oxidation Strategy for the Synthesis of Nitrones **10a–h** from Aldehyde **8** and Primary Amines **13a–h**^a^

a Concerning the synthesis of nitrone **10a** (Table 1, entry 1), this method compares very well with the previously reported condensation procedure of aldehyde **8** with *N*-benzyl hydroxylamine (85%),^14^ especially considering the difference of costs with benzylamine (**13a**).

The scope listed in Table 1 demonstrates the versatility of this method, which gives comparable results with complete conversions and good yields (70–80%) for benzylamines **13a–c** (entries 1–3), homobenzylamine **13d** (entry 4), and linear aliphatic amines **13e–f** (entries 5–6). Decrease in reaction yields with the α-branched (methyl)benzyl **13c** (entry 3) and aliphatic amines **13g** and **13h** (entries 7–8) suggests that the oxidation reaction is somewhat sensitive to steric effects. However, the corresponding nitrones **10c** and **10g–h** were still obtained in overall satisfactory yields (60–75%) through the two-step process. All the nitrones **10** were obtained exclusively as the more stable *Z*-diastereoisomer, in agreement with previous results (see also the Supporting Information section for 1D NOESY experiments on nitrone **10f**).^21^

**Scheme 2:**
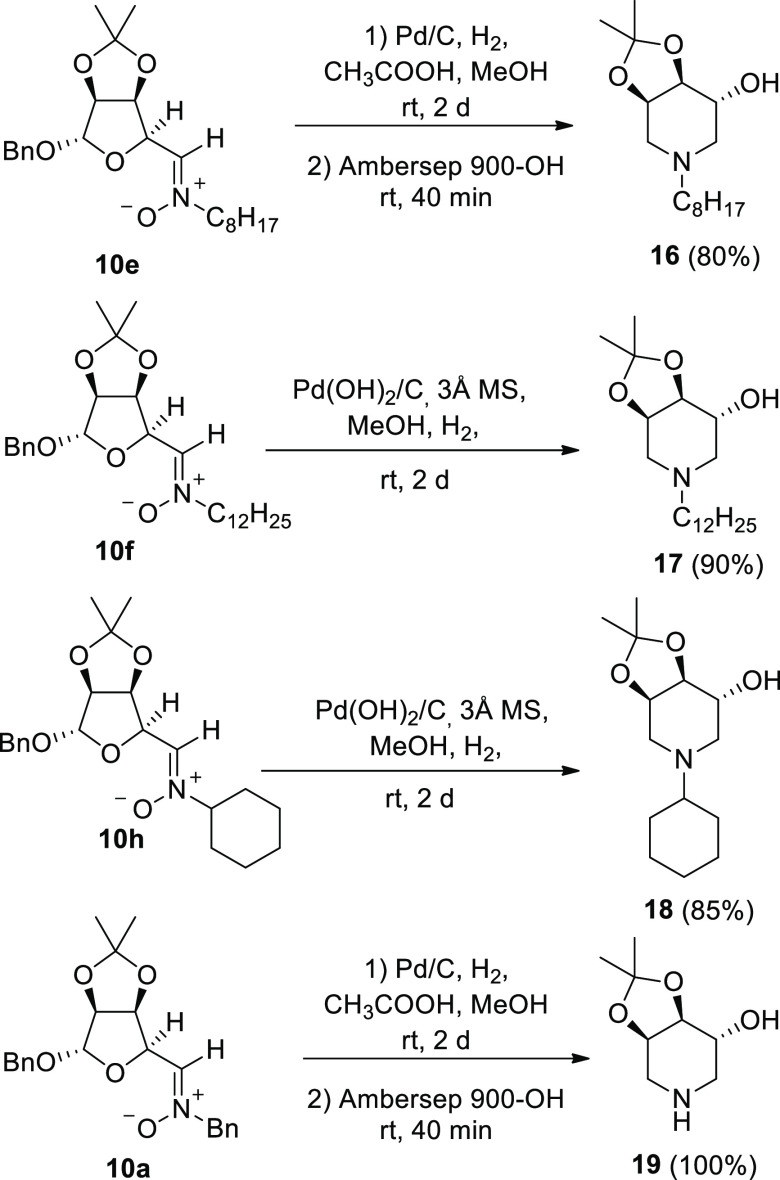
Reduction/Ring-Closure/RA Sequence to Trihydroxy Piperidines **16**–**19** from Nitrones **10**

Nitrones **10** turned out to be excellent starting materials for the straightforward synthesis of *N*-substituted trihydroxy piperidines. Indeed, treatment of the nitrones **10e**, **10f**, and **10h** in MeOH under H_2_ atmosphere (balloon) over Pd/C or Pd(OH)_2_/C as the catalyst afforded the *N*-substituted piperidines **16**,^11^**17**, and **18**, respectively, in excellent yields (Scheme 2). The overall efficiency of this one-pot process is remarkable, considering that it results from a cascade of several reactions, consisting of nitrone reduction, debenzylation, condensation with sugar aldehyde and iminium or enamine reduction (RA). Application of the same procedure to nitrone **10a** afforded the *N*-unsubstituted piperidine **19**(11) in quantitative yield.

**Scheme 3:**
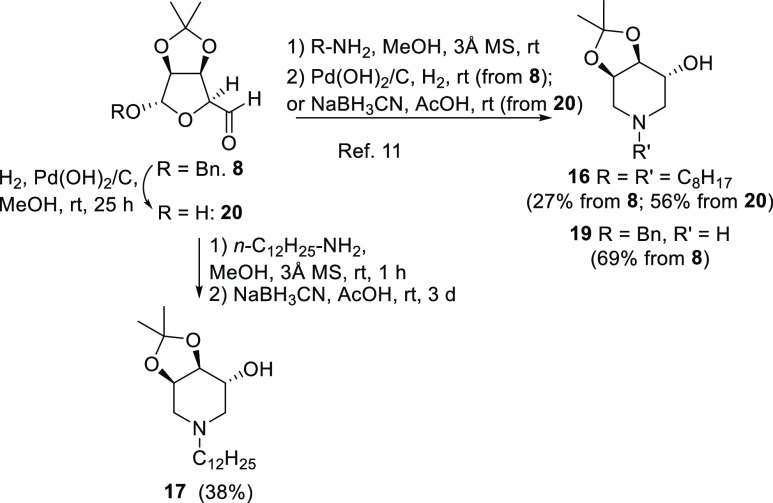
Previously Reported Strategies for the Synthesis of **16** and **19** Starting from the Aldehyde **8** and Alternative Synthesis of the Novel *N*-Dodecyl Trihydroxypiperidine **17**

It is worth to note that the yields obtained using this strategy were by far superior to those previously reported for the synthesis of piperidines **16** and **19** employing condensation of aldehyde **8** with primary amines and subsequent reduction to amine and RA-cyclization step, or, in alternative, employing a DRA strategy with NaBH_3_CN as the reducing agent on aldehyde **20**, in turn derived from **8**.^11^ (Scheme 3). For comparison, *N*-dodecyl trihydroxypiperidine **17**, obtained in 90% yield from nitrone **10f** (Scheme 2) was obtained also *via* DRA of **20** with dodecylamine in a modest 38% yield (Scheme 3).

**Scheme 4:**
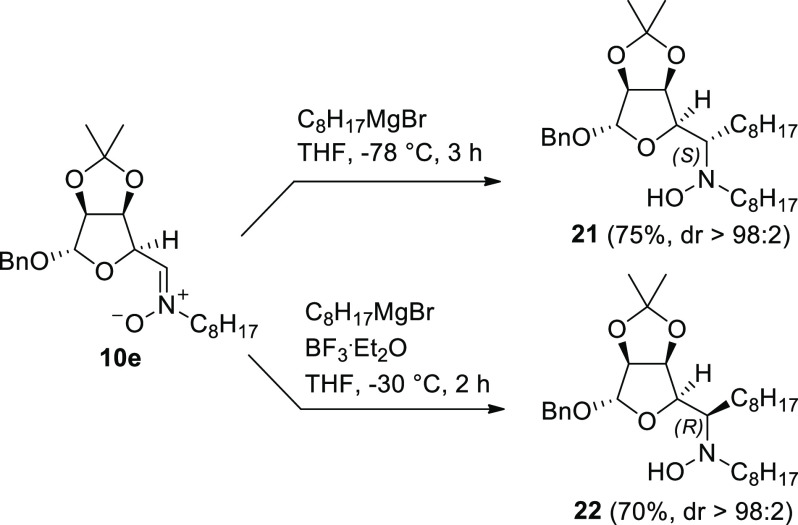
Addition of Octyl Magnesium Bromide (1.8 equiv) to Nitrone **10e** in the Presence or Absence of BF_3_·Et_2_O (1 equiv)

The synthetic strategy based on the addition of Grignard reagents to nitrone **10e** followed by intramolecular RA provided access to the desired 1,2-octyl trihydroxypiperidines **14** and **15** (Scheme 1). The length of the chains (C8) was chosen on the basis of the data previously obtained from biological assays. We had previously observed that the addition of Grignard reagents to the carbohydrate-derived nitrone **10a** proceeded with opposite diastereofacial preference in the presence or absence of a suitable Lewis acid. The addition reaction, followed by RA, allowed the introduction of different alkyl groups with opposite configurations at the piperidine C-2, affording two diastereomeric series of 2-alkyl trihydroxy piperidines.^14,16,17^ The addition of octylMgBr to nitrone **10e** was first carried out in THF at −78 °C for 3 h without any Lewis acid. The reaction gave smoothly the hydroxylamine **21**, with the *S* absolute configuration at the newly formed stereocenter accordingly to our prior work in good yield (75%) and excellent stereoselectivity (>98%) (Scheme 4). The configuration at the newly created stereocenter was unambiguously confirmed by ^1^H NMR and 1D NOESY spectra at a later stage of the synthesis (vide infra). The addition of octylMgBr to nitrone **10e** in the presence of BF_3_·Et_2_O at −30 °C for 2 h resulted in a complete reversal of the diastereoselectivity in favor of the epimeric hydroxylamine **22**, which was obtained with excellent stereoselectivity (>98%) and good yield (70%) with an *R* configuration at the newly formed stereocenter (Scheme 4).

Albeit the observed stereochemical outcome of the additions of octylMgBr to the nitrone **10e** in the absence or presence of BF_3_·Et_2_O is in agreement with the results obtained previously in the additions of several Grignard reagents to nitrone **10a**, we were delighted to see that in this case a complete diastereoselectivity was reached. Instead, in the Grignard additions to **10a**, we had observed diastereomeric ratios in the range 1.4–5.6:1 without Lewis acids and 3–9:1 with BF_3_·Et_2_O in favor of the (*S*) and (*R*) configured adducts, respectively.^16,17^

**Figure 3:**
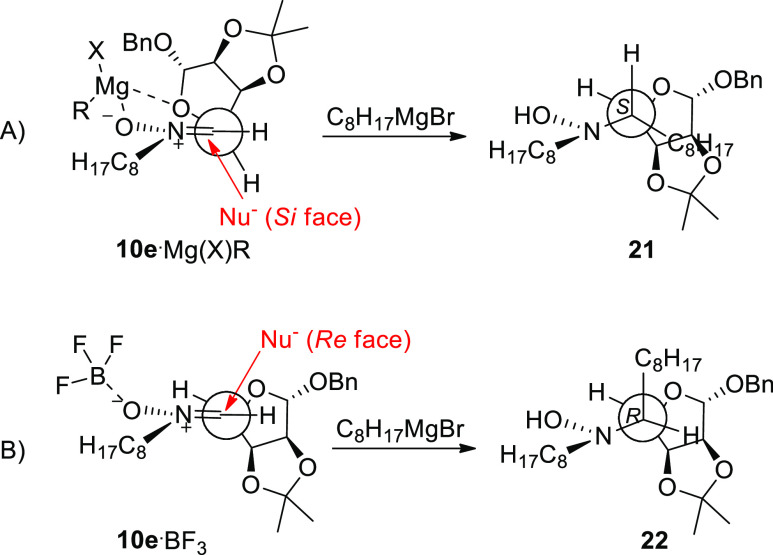
Transition state models proposed for the nucleophilic attack to nitrone **10e**: (A) cram-chelate TS in the absence of Lewis acid; (B) TS in the presence of BF_3_·Et_2_O.

A magnesium Cram-chelate transition state (TS) may account for the preferred nucleophilic attack to the *Si* diastereoface of nitrone **10e** in the absence of Lewis acid, resulting in the exclusive formation of the hydroxylamine **21** with the *S* configuration at the newly formed stereocenter (Figure 3A). Conversely, when an equimolar amount of BF_3_·Et_2_O is added, chelation is prevented, thus favoring the nucleophilic attack to the more accessible *Re* face of nitrone **10e**, which leads to the hydroxylamine **22** with the *R* configuration at the newly formed stereocenter (Figure 3B).^14,17^

**Scheme 5:**
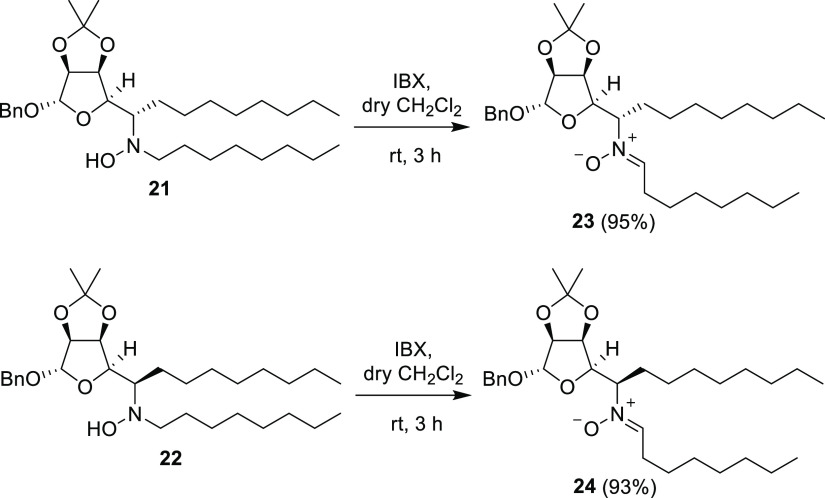
Oxidation of Hydroxylamines **21** and **22** with IBX: Synthesis of Nitrones **23** and **24**

The hydroxylamines **21** and **22** showed high tendency to oxidize spontaneously in air to the corresponding aldonitrones **23** and **24** (Scheme 5), according to the behavior observed for other hydroxylamines obtained by addition of Grignard reagents to the *N*-benzyl nitrone **10a**; notwithstanding the C=N bond does not benefit of conjugation in the present case.^16,17^ In particular, spontaneous partial oxidation of **21** and **22** occurred with 50% conversion, as evaluated by ^1^H NMR spectroscopy. The exclusive formation of aldonitrones instead of ketonitrones was providential, since it did not trigger any loss of configurational integrity at the newly formed stereocenter and was not detrimental for our synthetic purposes (see below).

However, a certain regioselectivity in favor of the aldonitrones was expected based on our previous studies.

Due to the low stability of hydroxylamines **21** and **22**, they were characterized only by ^1^H NMR and MS analyses immediately after purification by column chromatography. Characterization of pure nitrones **23** and **24** was carried out after complete oxidation of the hydroxylamines **21** and **22** with the hypervalent iodine reagent 2-iodoxy benzoic acid (IBX), which is the reagent of choice to promote regioselective oxidation of *N*,*N*-disubstituted hydroxylamines in favor of the corresponding aldonitrones.^13,25^ In this case, it provided the corresponding nitrones **23** and **24** in excellent yields and with complete regioselectivity (Scheme 5).

**Scheme 6:**
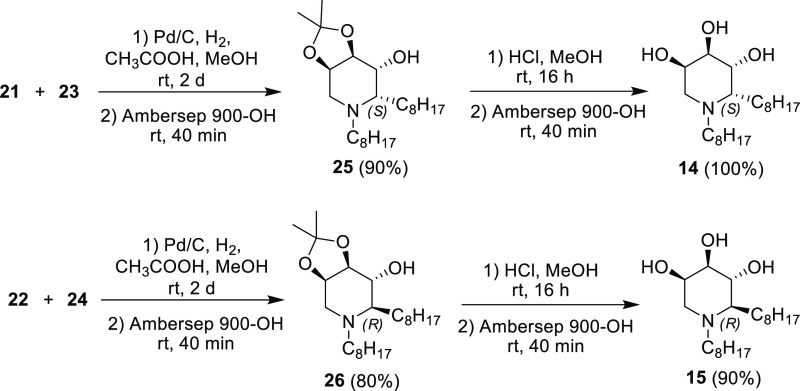
Ring-Closure/Reductive Amination to **25** and **26** and Their Final Deprotection to 1,2-Dioctyl Trihydroxypiperidines **14** and **15**

The final reductive amination step was performed on the hydroxylamine/nitrone mixtures by employing 2 equiv of acetic acid under an H_2_ atmosphere (balloon) and Pd/C as the catalyst in MeOH (0.015 M solution), followed by treatment with a strongly basic resin to give the free amines **25** and **26** in excellent 90 and 80% yield, respectively (Scheme 6).

**Figure 4:**
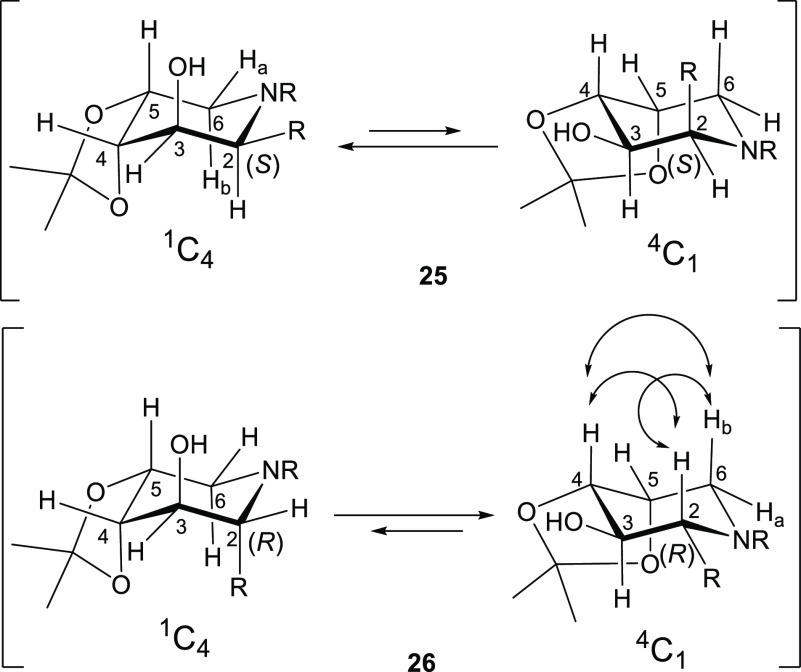
Chair conformations of piperidines **25** and **26** (R = *n*C_8_H_17_). Double-ended arrows show the observed diagnostic nOe correlation peaks.

Careful analysis of the ^1^H NMR, 2D NMR (COSY, HSQC), and 1D NOESY spectra of piperidines **25** and **26** allowed us to establish unambiguously their configuration, thus validating the stereochemical outcome of the additions of octylMgBr reported in Scheme 4. The two chair conformations of each compound are reported in Figure 4. The ^1^H NMR spectrum of (2*S*) piperidine **25** showed a small coupling constant between 3-H and 4-H (^3^*J*_3–4_ = 4.0 Hz), suggesting a high preference for its ^1^C_4_ conformation where both 3-H and 4-H are equatorial. The ^1^C_4_ is expected to be the absolute minimum energy conformation for the *S*-configured piperidine at C-2, with the octyl chain lying in equatorial position (Figure 4). Accordingly, the 1D NOESY spectrum of **25** did not show the nOe correlation peak between 2-H and 4-H. For the diastereomeric piperidine **26**, the opposite *R* configuration at C-2 shifted the equilibrium to a preferred ^4^C_1_ conformation in order to accommodate again the octyl chain in an equatorial position, as attested by the increase of the coupling constant between 3-H and 4-H (^3^*J*_3–4_ = 8.0 Hz) in agreement with their *ax–ax* relationship (Figure 4). The set of strong nOe correlation peaks observed in the 1D-NOESY spectrum of **26** among all axial protons (2-H with 4-H and 6-H_b_ and 4-H with 6-H_b_ 2-H, see the Supporting Information section) substantiated this assignment.

Final deprotection of the acetonide protecting groups under acidic conditions (aqueous HCl in MeOH) followed by treatment with the strongly basic resin Ambersep 900-OH gave the target 1,2-dioctyl trihydroxypiperidines **14** and **15** as free amines in excellent yields (Scheme 6).

**Scheme 7:**
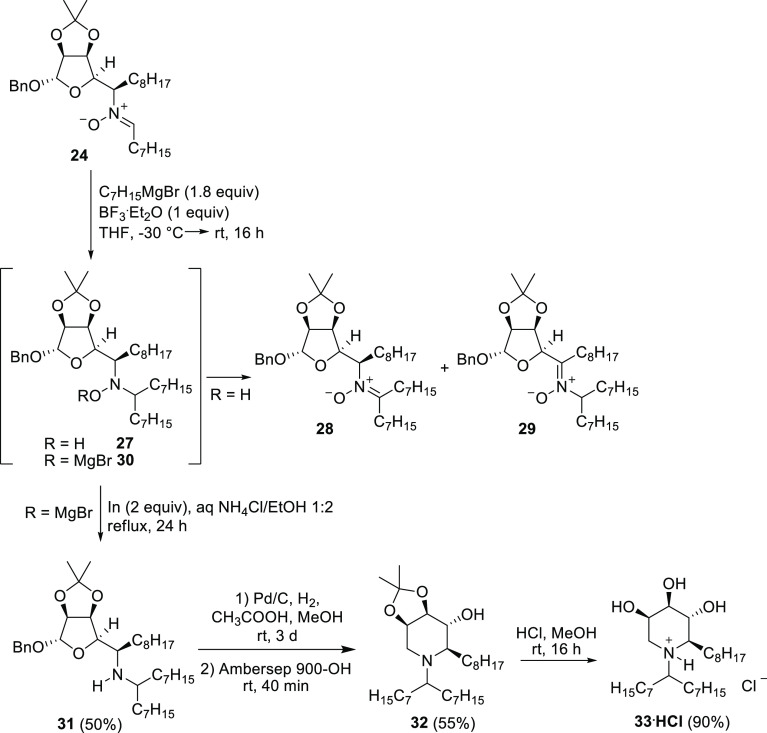
Addition of heptylMgBr to Nitrone **24**, One-Pot Addition/Reduction to Amine **31**, and Synthesis of the Three-Tailed Trihydroxypiperidine **33·HCl**

We envisaged that the intriguing formation of the aldonitrones **23** and **24** by easy and regioselective oxidation of hydroxylamines **21** and **22** would furnish the chance for further extending our study. Indeed, another alkyl chain might be introduced *via* iteration of the Grignard addition to these nitrones to provide final piperidines possessing three lipophilic tails. This hypothesis was proven with nitrone **24**. In order to avoid formation of a further stereogenic center resulting in a mixture of diastereoisomers, heptyl magnesium bromide was chosen as the appropriate Grignard reagent, but the addition turned out to be sluggish. In the absence of the promoter at different temperatures (−30 °C, 0 °C, rt), in THF, it failed to give any product. Addition of BF_3_·Et_2_O (1.0 equiv) resulted in the formation of the desired hydroxylamine **27** after 16 h (Scheme 7), as attested by the peak at *m*/*z*: 618.31 ([M + H]^+^) at an ESI-MS analysis. The hydroxylamine **27** was unstable and presumably underwent mainly rapid oxidation to the corresponding ketonitrones **28** and **29**, as suggested by the presence of two TLC spots with very similar R_*f*_ and a peak at 638.55, corresponding to [M + Na]^+^, in the ESI-MS spectrum (Scheme 7).

The lack of regioselectivity in the oxidation of **27** is a major drawback in view of our synthetic target, since the ketonitrone **29** has lost the stereochemical information to be installed at C-2 in the final piperidine. In order to prevent the rapid oxidation of the hydroxylamine **27**, the adduct **30** was reduced *in situ* to the corresponding amine **31** by direct addition to the crude reaction mixture of indium powder in slightly acidic aqueous ethanol.^26^ This one-pot Grignard addition/reduction afforded the amine **31** in 50% yield without any loss of stereochemical integrity (Scheme 7). The RA of amine **31** with H_2_ as the reducing agent in the presence of catalytic Pd/C and acetic acid (2 equiv) in MeOH provided the partially protected trihydroxy piperidine **32** in 55% yield (Scheme 7). Final deprotection of the acetonide performed under acidic conditions (aqueous HCl in MeOH) led to the desired trihydroxy piperidine **33** as the hydrochloride salt (Scheme 7).^27^

### 2.3 Biology and Molecular Dynamics

#### 2.3.1 Preliminary Biological Evaluation toward Commercial Glycosidases

Preliminary biological evaluation of compounds **14** and **15** (at 100 μM inhibitor concentration) was performed toward a panel of 12 commercial glycosidases (α-l-fucosidase EC 3.2.1.51 from *Homo sapiens*, α-galactosidase EC 3.2.1.22 from coffee beans, β-galactosidases EC 3.2.1.23 from *Escherichia coli* and *Aspergillus oryzae*, α-glucosidases EC 3.2.1.20 from yeast and rice, amyloglucosidase EC 3.2.1.3 from *Aspergillus niger*, β-glucosidase EC 3.2.1.21 from almonds, α-mannosidase EC 3.2.1.24 from Jack beans, β-mannosidase EC 3.2.1.25 from snail, and β-*N*-acetylglucosaminidases EC 3.2.1.52 from Jack beans and bovine kidney).

No remarkable inhibitory activity was found at this concentration for compounds **14** and **15** toward any of these enzymes apart from a 30% inhibition of β-glucosidase from almonds by compound **14** (see the Supporting Information section). Even if moderate, this value was encouraging, since we have previously noticed compounds with a low inhibitory activity toward this commercial enzyme that turned out to be stronger inhibitors of human lysosomal GCase.^12,17^

#### 2.3.2 Inhibitory Activity of GCase

Compounds **14**, **15**, and **33·HCl** were then tested at 1 mM for GCase inhibition in human leukocyte homogenates. The percentages of inhibition, together with the corresponding IC_50_ values, are shown in Table 2. The results obtained were compared with data of previously reported compounds **9**, **11**, and **12**.^12,16,17^

**Table 2:** GCase Inhibition and IC_50_ Values in Human Leukocytes from Healthy Donors

entry	compound	GCase inhibition [%]^a^	IC_50_ (μM)^b^
1	**9**	98	30.0 ± 1.0^c^
2	**11**	80	93.5 ± 5.3^d^
3	**12**	100	29.3 ± 1.8^d^
4	**14**	100	100.0 ± 9.0
5	**15**	100	15.0 ± 4.0
6	**33·HCl**	75	130 ± 15

a Percentage inhibition of GCase in human leukocytes extracts incubated with azasugars (1 mM).

b IC_50_ values were determined by measuring GCase activity at different concentrations of each inhibitor.

c Ref (12) (IC_50_ value obtained using the same experimental protocol and substrate concentration of this manuscript).

d Refs (16) and (17) (IC_50_ value obtained using the same experimental protocol and substrate concentration of this manuscript).

Our results show that both the newly synthesized trihydroxypiperidines **14** and **15** were able to strongly inhibit GCase, imparting 100% inhibition of the enzyme at 1 mM concentration (Table 2, entries 4–5). However, a remarkable difference emerged between the two compounds from measurement of their IC_50_, which followed the same trend observed for the C-2 mono-alkylated azasugars **11** and **12.** Indeed, the 1,2-dioctyl azasugar **15** with the *R* configuration at C-2 was a stronger inhibitor than its *S*-configured diastereoisomer **14** (IC_50_ = 15.0 μM *vs* IC_50_ = 100 μM, Table 2 entry 5 *vs* 4). In this latter case, the difference was even more pronounced than for the corresponding monoalkylated congeners **12** and **11**. It is also worth to note that the dialkylated piperidine **15** is twofold more active than its monoalkylated counterpart **12**. Apparently, the presence of a second octyl chain at the nitrogen atom imparted beneficial interactions within the enzyme active site only in the case of the 2*R*-configured pair. Conversely, the 1,2-dialkylated azasugar **33·HCl**, although maintaining the octyl chain at C-2 with *R* configuration is a much less potent GCase inhibitor than **15** (IC_50_ = 130.0 μM *vs* IC_50_ = 15.0 μM, Table 2 entry 6 *vs* 5), suggesting that the presence of a further alkyl chain is detrimental.

#### 2.3.3 Molecular Dynamic Studies

**Figure 5:**
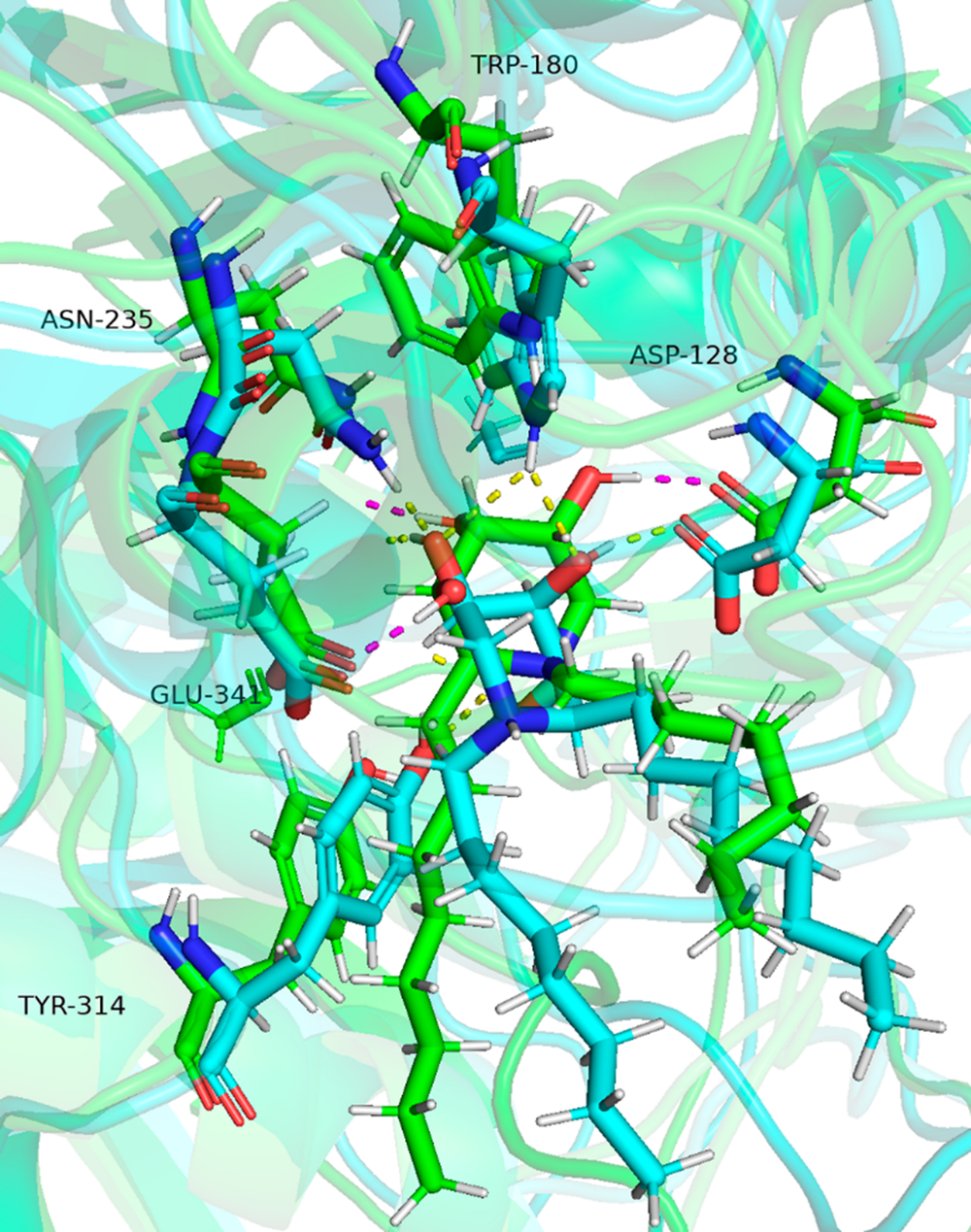
Close-up view of GCase in complex with compounds (*R*)-**15H**^**+**^^4^C_1_ (cyan) with (*R*) configuration at C-2 and (*R*)-**14H**^**+**^^4^C_1_ (green) with (*S*) configuration at C-2. Dashed yellow and magenta lines indicate H-bond interactions of (*R*)-**15H**^**+**^^4^C_1_ and (*R*)-**14H**^**+**^^4^C_1_, respectively. It can be appreciated that the former has more polar contacts with the residues of the binding site than the latter.

We carried out MD simulations for (*R*) and (*S*) protonated forms of compounds **14** and **15**, considering both conformations ^4^C_1_ and ^1^C_4_ as starting points and taken into consideration the best poses obtained from the docking studies and also the best ones in which the piperidine ring is correctly oriented as IFG. In all cases, MD converged to stable complexes which were reached after 10 ns of simulation and continued to be stable for 250 ns (all MD simulations were replicated four times). The complexes showed the ligand in the binding site establishing different interactions with key residues Asp128, Trp180, Asn235, Glu236, Tyr314, and Glu341 and with the correct orientation of the piperidine ring (see the Supporting Information section).^28^ The overall RMSD for the protein system appeared to have reached equilibrium in the first ns, and the stabilization of the protein–ligand complex after 20 ns keeping the interactions of the complexes constant during the rest of the simulation. The ligands showed a complete stability of chair conformations and tend to arrange the aliphatic chains toward the external part, exposed to the solvent, in order to keep interactions of the hydroxyl groups of the piperidine ring. The presence of the alkyl chains induces some visible conformational change of the protein in the area close to the binding site. Interestingly, similar protein conformations were observed for (*R*)-**14H**^**+**^^4^C_1_ and (*S*)–**14H**^**+**^^1^C_4_, whereas a loop formed by residues 345–349 is partially opened with (*R*)- **14H**^**+**^^1^C_4_ and completely opened with (*S*)-**14H**^**+**^^4^C_1_ (see the Supporting Information section). In the case of compound **15H**^**+**^, the protein shows a higher flexibility with the loop formed by residues 345–349 adopting different orientations. The mobility of the loop, rich in hydrophobic residues (Met347, Phe348, and Trp349) can be attributable to hydrophobic interactions with alkyl chains that stabilizes the complex as expected. The sole inspection of the interactions in the binding site shows that (*R*)-**15H**^**+**^^4^C_1_ is the complex having the highest stability (Figure 5).

With the aim of obtaining more accurate information on the stability of the complexes (rather than the qualitative observation of the interactions), we carried out additional calculations to evaluate the binding energy. For the purpose of comparison both MM/GBSA and MM/PSBA calculations were performed.^29^ In both cases, higher values were obtained for compound **15**, the best values being found for (*R*)-**15H**^**+**^^4^C_1_ (*E*_binding_ = −57.9 kcal/mol with MM/GBSA and −49.4 kcal/mol with MM/PBSA) corroborating the observed interactions and in good agreement with experimental results (see the Supporting Information section).

#### 2.3.4 Preliminary Biological Screening toward Human Lysosomal Glycosidases

In order to avoid undesired inhibitory side effects of a potential new drug, it has to be selective for a given target. The selectivity of the newly synthesized compounds **14**, **15**, and **33·HCl** toward GCase was then investigated, evaluating their inhibition at 1 mM concentration toward six other lysosomal glycosidases (namely, α-and β-mannosidases, α- and β-galactosidases, α-fucosidase, and α-glucosidase) in cell homogenates (leucocytes or lymphocytes) isolated from healthy donors. The results, reported in the Supporting Information section, clearly show that these compounds are selective inhibitors of GCase. Indeed, only moderate inhibitory activity (*ca.* 50%) was found toward human β-galactosidase for both piperidines **14** and **15**, while negligible inhibitory activity was detected in all other cases.

#### 2.3.5 Pharmacological Chaperoning Activity

The ability of compounds **14** and **15** to enhance the activity of GCase after incubation (4 days) with fibroblasts bearing the selected mutations was evaluated. The experiments were performed using fibroblasts derived from Gaucher patients bearing the N370S/RecNcil and L444P/L444P mutations (see the Supporting Information section). Unfortunately, no enzymatic activity rescue was observed after incubation with increasing concentrations of compounds **14** and **15** from 5 nM to 10 μM. The assay could not performed at higher concentration (50 or 100 μM), since the low cell viability observed hampered the measurement of the enzymatic activity.

These data show that the simultaneous presence of an octyl chain at both the endocyclic nitrogen and C-2 of the piperidine skeleton makes these compounds too cytotoxic at the highest concentrations, while at the lowest concentrations, no activity rescue was observed.

The low viability detected above 50 μM concentration may be ascribed to interactions of **14** and **15** with lipids and proteins of the cell membranes which lead to cell lysis, as it was previously reported for other amphiphilic compounds when tested *in vitro*.^30^

Expecting an even higher impact on cell viability from compound **33·HCl**, which has an additional lipophilic chain, a preliminary MTT test was carried out on wild-type fibroblasts (see Supporting Information). Since a remarkable cytotoxicity was observed for prolonged incubation at 100 and 50 μM concentrations, compound **33·HCl** was co-incubated in Gaucher patients’ fibroblasts (bearing the N370S/RecNcil mutation) in lower concentrations, ranging from 5 nM to 30 μM. Unfortunately, also in this case, no enzymatic activity rescue was observed after 4 days of incubation.

## 3 Conclusions

Following our interest in the discovery of new inhibitors and/or pharmacological chaperones for the GCase enzyme, we investigated the condensation/oxidation reaction of carbohydrate-derived aldehyde **8** with several primary amines employing urea hydrogen peroxide as the stoichiometric oxidant and methyltrioxorhenium as the catalyst. This strategy afforded in a simple and straightforward way several new *C*-carbohydrate *N*-alkyl nitrones **10a–h**. Further ring-closure reductive amination provided a series of new precursors of trihydroxypiperidine azasugars bearing different substituents at the nitrogen atom much more effectively than previously reported.

The reaction of the *C*-erythrosyl *N*-octyl nitrone **10e** with octylMgBr in the presence or absence of BF_3_·Et_2_O provided access to both epimeric hydroxylamines with opposite configuration at the newly created stereocenter in a stereodivergent and completely stereoselective way. Final reductive amination and acetonide deprotection provided compounds **14** and **15** from low-cost d-mannose in remarkable 43 and 32% overall yields, respectively, over eight steps. A third alkyl chain was introduced by iteration of the organometal addition to a nitrone easily obtained by oxidation with complete regioselectivity after the first addition, followed by the one-pot hydroxylamine/amine reduction mediated by indium metal. This strategy provided the azasugar **33·HCl** bearing three alkyl chains.

The new compounds, assayed toward commercial and human lysosomal glucosidases, proved to be good and selective GCase (human lysosomal β-glucosidase) inhibitors. In particular, the C-2 *R*-configured bis-alkylated trihydroxypiperidine **15** showed a lower IC_50_ than the corresponding mono-alkylated analogue **12** (IC_50_ = 15 μM *vs* IC_50_ = 29.3 μM). Conversely, the presence of a third alkyl tail as in compound **33·HCl** affected negatively the GCase inhibitory activity (IC_50_ = 130 μM).

However, once tested in fibroblasts derived from Gaucher patients bearing the N370S/RecNcil and L444P/L444P mutations, no GCase activity enhancements were observed, demonstrating that the addition of one (**14** and **15**) or two alkyl chains (**33·HCl**) strongly increases the cytotoxicity of the compounds and it is detrimental for the pharmacological chaperoning activity.

Preliminary docking studies suggested compound **15** as the best ligand although the preferred conformation and orientation were not envisaged. Further MD simulations identified the protonated derivative of **15** with (*R*) configuration at the nitrogen atom and adopting a ^4^C_1_ conformation as the best ligand. The different isomers and the corresponding conformers induce a substantial change in the conformation of a loop, composed by residues 345–349, to accommodate the aliphatic alkyl chains. MM/GBSA calculations corroborated the preference by (*R*)-**15H**^**+**^^4^C_1_ with a binding energy of −57.9 kcal/mol.

## 4 Experimental Section

### 4.1 Chemistry

#### 4.1.1 General Methods

Commercial reagents were used as received. All reactions were carried out under magnetic stirring and monitored by TLC on 0.25 mm silica gel plates (Merck F254). Column chromatographies were carried out on Silica Gel 60 (32–63 μm) or on silica gel (230–400 mesh, Merck). Yields refer to spectroscopically and analytically pure compounds unless otherwise stated. ^1^H NMR spectra were recorded on a Varian Gemini 200 MHz, a Varian Mercury 400 MHz, or on a Varian INOVA 400 MHz instruments at 25 °C. ^13^C NMR spectra were recorded at 50 MHz or at 100 MHz. Chemical shifts are reported relative to CDCl_3_ (^1^H: δ = 7.27 ppm, ^13^C: δ = 77.0 ppm). Integrals are in accordance with assignments, coupling constants are given in Hz. For detailed peak assignments, 2D spectra were measured (g-COSY, g-HSQC) and 1D-NOESY. The following abbreviations were used to designate multiplicities: s = singlet, d = doublet, t = triplet, q = quartet, m = multiplet, quin = quintuplet, sext = sextet, sept = septet, br s = broad singlet, and dd = double-doublet. IR spectra were recorded with an IRAffinity-1S Shimadzu spectrophotometer. ESI-MS spectra were recorded with a Thermo Scientific LCQ fleet ion trap mass spectrometer. Elemental analyses were performed with a Thermo Finnigan FLASH EA 1112 CHN/S analyzer. Optical rotation measurements were performed on a JASCO DIP-370 polarimeter.

#### 4.1.2 General Procedure for the Synthesis of Nitrones

Amines **13a–h** were purchased from Merck (**13a**, **13c**, **13f**, and**13h**), Fluka (**13d**, **13g**), TCI (**13e**), Janssen (**13b**) and used without further purification.

To a stirred solution of aldehyde **8** (200 mg, 0.72 mmol) and anhydrous Na_2_SO_4_ (380 mg) in MeOH (9 mL) at room temperature, the appropriate amine **13a–h** (1.2 equivalents) was added and the resulting mixture was stirred at room temperature under nitrogen atmosphere until ^1^H NMR control attested the formation of imine intermediates (3 h). The reaction mixture was cooled at 0 °C and urea–hydrogen peroxide (UHP, 203 mg, 2.16 mmol), and methyltrioxorhenium (MTO, 7.2 mg, 0.29 mmol) were added sequentially. The reaction mixture was stirred at room temperature for 16 h, when a TLC control (PEt/EtOAc 1:1) attested the disappearance of the starting material and the presence of a new UV visible spot; then, the solvent was removed under reduced pressure. CH_2_Cl_2_ was added to the crude mixture, and the undissolved urea was filtered off. Solvent removal under reduced pressure afforded the crude product, which was purified by flash column chromatography on silica gel.

#### 4.1.3 Synthesis of (*Z*)-Benzyl 2,3-*O*-(1-methylethylidene)-5-deoxy-*N*-benzyl-d-lyxofuranosylamine *N*-Oxide (**10a**)^14^

Application of the general procedure to 200 mg (0.72 mmol) of **8** with 94 μL (0.86 mmol) of benzylamine (**13a**) furnished, after purification by column chromatography (PEt/EtOAc 3:1), 221 mg (0.56 mmol, 80%) of **10a** as a white solid (R_*f*_ = 0.40, PEt/EtOAc 2:1).

**10a**: white solid. ^1^H NMR (400 MHz, CDCl_3_): δ 7.37–7.19 (m, 10H, Ar), 6.75 (dd, *J* = 4.4, 2.4 Hz, 1H), 5.11–5.07 (m, 2H), 5.04 (s, 1H), 4.89 (s, 2H), 4.61–4.58 (m, 2H), 4.37 (d, *J* = 11.7 Hz, 1H), 1.30 (s, 3H), 1.21 (s, 3H) ppm. ^13^C{1H} NMR (50 MHz, CDCl_3_): δ 137.0, 135.6, 132.4, 129.4–127.9 (10 C), 112.6, 105.0, 84.5, 79.7, 76.7, 69.1, 68.9, 26.0, 24.7 ppm.

#### 4.1.4 Synthesis of (*Z*)-Benzyl 2,3-*O*-(1-methylethylidene)-5-deoxy-*N*-(2-methoxybenzyl)-d-lyxofuranosylamine *N*-Oxide (**10b**)

Application of the general procedure to 200 mg (0.72 mmol) of **8** with 112 μL (0.86 mmol) of 2-methoxybenzylamine (**13b**) furnished, after purification by column chromatography (PEt/EtOAc 2:1), 238 mg (0.58 mmol, 80%) of **10b** as a white solid (R_*f*_ = 0.20, PEt/EtOAc 2:1).

**10b**: white solid. mp 118–120 °C. [α]_D_^25^ + 55.9 (*c* 0.65, CHCl_3_). ^1^H NMR (400 MHz, CDCl_3_): δ 7.42–7.26 (m, 7H, Ar), 6.99 (t, *J* = 6.6 Hz, 1H, Ar), 6.92 (d, *J* = 8.0 Hz, 1H, Ar), 6.79 (d, *J* = 4.0 Hz, 1H, *H*C=N), 5.17–5.10 (m, 2H, 4-H and 3-H), 5.09 (s, 1H, 1-H), 4.99 AB system, *J* = 12.0 Hz, 2H NC*H*_*2*_Ar, 4.67 (d, *J* = 12.0 Hz, 1H, OC*H*_2_Ar), 4.66–4.65 (m, 1H, 2-H), 4.43 (d, *J* = 12.0 Hz, 1H, OC*H*_2_Ar), 3.85 (s, 3H, OC*H*_*3*_), 1.35 (s, 3H, C(C*H*_*3*_)_2_), 1.27 (s, 3H, C(C*H*_*3*_)_2_) ppm. ^13^C{1H} NMR (100 MHz, CDCl_3_): δ 157.9 (Ar-OCH_3_), 137.1 (Ar), 135.3 (*C*=N), 132.0 (Ar), 130.9 (Ar), 128.6–128.0 (5C, Ar), 121.0 (Ar), 120.8 (Ar), 112.6 (*C*(CH_3_)_2_), 110.8 (Ar), 105.0 (C-1), 84.6 (C-2), 79.8 (C-3), 76.8 (C-4), 69.0 (O*C*H_2_Ar), 64.0 (N*C*H_2_Ar), 55.2 (O*C*H_3_), 26.2 (C(*C*H_3_)_2_), 24.8 (C(*C*H_3_)_2_) ppm. MS (ESI) *m*/*z* (%): 849.0 (100) [2M + Na]^+^, 436.1 (82) [M + Na]^+^. IR (CDCl_3_) ν: 976, 1159, 1254, 1379, 1462, 1496, 1606, 2241, 2843, 2941, 3034 cm^–1^. Anal. Calcd for C_23_H_27_NO_6_: C, 66.81; H, 6.58; N, 3.39. Found: C, 67.01; H, 6.50; N, 3.20.

#### 4.1.5 Synthesis of (*Z*)-Benzyl 2,3-*O*-(1-methylethylidene)-5-deoxy-*N*-(1-phenylethyl)-d-lyxofuranosylamine *N*-Oxide (**10c**)

Application of the general procedure to 200 mg (0.72 mmol) of **8** with 110 μL (0.86 mmol) of (*S*)-(−)-1-phenylethylamine (**13c**) furnished, after purification by column chromatography (PEt/EtOAc 2:1), 214 mg (0.34 mmol, 75%) of **10c** as a white solid (R_*f*_ = 0.20, PEt/EtOAc 2:1).

**10c**: white solid. mp 97–99 °C. [α]_D_^25^ – 5.4 (*c* = 0.86, CHCl_3_). ^1^H NMR (400 MHz, CDCl_3_): δ 7.50 (d, *J* = 8.0 Hz, 2H, Ar), 7.38–7.26 (m, 8H, Ar), 6.79 (d, *J* = 4.0 Hz, 1H, *H*C = N), 5.16–5.15 (m, 2H, 4-H and 3-H), 5.13 (s, 1H, 1-H), 5.10 (q, *J* = 6.8 Hz, 1H, (CH_3_)C*H*Ar), 4.69 (d, *J* = 12.0 Hz, 1H, OC*H*_2_Ar), 4.66–4.65 (m, 1H, 2-H), 4.45 (d, *J* = 12.0 Hz, 1H, OC*H*_2_Ar), 1.84 (d, *J* = 6.8 Hz, 3H, (C*H*_*3*_)CHAr), 1.35 (s, 3H, C(C*H*_*3*_)_2_), 1.25 (s, 3H, C(C*H*_*3*_)_2_) ppm. ^13^C{1H} NMR (100 MHz, CDCl_3_): δ 137.8 (Ar), 136.9 (Ar), 134.2 (H*C*=N), 128.7–127.3 (10C, Ar), 112.4 (*C*(CH_3_)_2_), 104.8 (C-1), 84.4 (C-2), 79.6 (C-3), 76.7 (C-4), 73.2, ((CH_3_)*C*HAr), 68.9 (O*C*H_2_Ar), 26.0 (C(*C*H_3_)_2_), 24.7 (C(*C*H_3_)_2_), 18.7 ((*C*H_3_)CHAr) ppm. MS (ESI) *m*/*z* (%): 817.0 (100) [2M + Na]^+^, 420.12 (61) [M + Na]^+^. IR (CDCl_3_) ν: 970, 1382, 1456, 1496, 1608, 2249, 2873, 2938, 2983, 3034, 3406 cm^–1^. Anal. Calcd for C_23_H_27_NO_5_: C, 69.50; H, 6.85; N, 3.52. Found: C, 69.45; H, 6.96; N, 3.65.

#### 4.1.6 Synthesis of (*Z*)-Benzyl 2,3-*O*-(1-methylethylidene)-5-deoxy-*N*-[2-(3-methoxyphenyl)ethyl]-d-lyxofuranosylamine *N*-Oxide (**10d**)

Application of the general procedure to 200 mg (0.72 mmol) of **8** with 120 μL (0.86 mmol) of 2-(3-methoxyphenyl)ethylamine (**13d**) furnished, after purification by column chromatography (PEt/EtOAc 2:1), 246 mg (0.58 mmol, 80%) of **10d** as a white solid (R_*f*_ = 0.20, PEt/EtOAc 2:1).

**10d**: white solid. mp 87–89 °C. [α]_D_^25^ – 18.0 (*c* 0.90, CHCl_3_). ^1^H NMR (400 MHz, CDCl_3_): δ 7.32–7.21 (m, 5H, Ar), 7.17 (t, *J* = 7.4 Hz, 1H, Ar), 6.77 (d, *J* = 8.0 Hz, 1H, Ar), 6.74 (d, *J* = 8.0 Hz, 1H, Ar), 6.73 (s, 1H, Ar), 6.67 (d, *J* = 5.2 Hz, 1H, *H*C=N), 5.10 (pseudo t, *J* = 4.2 Hz, 1H, 4-H), 5.06–5.04 (m, 2H, 3-H and 1-H), 4.63 (d, *J* = 12.0 Hz, 1H, OC*H*_2_Ar), 4.62–4.60 (m, 1H, 2-H), 4.40 (d, *J* = 12.0 Hz, 1H, OC*H*_2_Ar), 4.01–3.95 (m, C*H*_*2*_N), 3.73 (s, 3H, OC*H*_*3*_), 3.18 (t, *J* = 7.2 Hz, 2H, NCH_2_C*H*_*2*_Ar), 1.30 (s, 3H, C(C*H*_*3*_)_2_), 1.23 (s, 3H, C(C*H*_*3*_)_2_) ppm. ^13^C{1H} NMR (100 MHz, CDCl_3_): δ 159.9 (Ar-OCH_3_), 139.0 (Ar), 137.0 (Ar), 135.5 (H*C*=N), 129.8 (Ar), 128.5–128.0 (5C, Ar), 121.0 (Ar), 114.4 (Ar), 112.7 (*C*(CH_3_)_2_), 112.5 (Ar), 105.1 (C-1), 84.7 (C-2), 79.8 (C-3), 76.3 (C-4), 68.9 (O*C*H_2_Ar), 66.1 (N*C*H_2_CH_2_Ar), 55.2 (O*C*H_3_), 33.8 (NCH_2_*C*H_2_Ar), 26.1 (C(*C*H_3_)_2_), 24.7 (C(*C*H_3_)_2_) ppm. MS (ESI) *m*/*z* (%): 450.13 (100) [M + Na]^+^. IR (CDCl_3_) ν: 976, 1078, 1159, 1211, 1379, 1460, 1490, 1604, 2241, 2839, 2876, 2941, 3032 cm^–1^. Anal. Calcd for C_24_H_29_NO_6_: C, 67.43; H, 6.84; N, 3.28. Found: C, 67.40; H, 6.85; N, 3.22.

#### 4.1.7 Synthesis of (*Z*)-Benzyl 2,3-*O*-(1-methylethylidene)-5-deoxy-*N*-octyl-d-lyxofuranosylamine *N*-Oxide (**10e**)

Application of the general procedure to 300 mg (1.08 mmol) of **8** with 215 μL (1.30 mmol) of octylamine (**13e**) furnished, after purification by column chromatography (Hex/EtOAc 2:1), 350 mg (0.86 mmol, 80%) of **10e** as a white solid (R_*f*_ = 0.34, Hex/EtOAc 2:1).

**10e**: white solid. mp 64–66 °C. [α]_D_^23^ – 8.2 (*c* 0.65, CHCl_3_). ^1^H NMR (400 MHz, CDCl_3_): δ 7.32–7.24 (m, 5H, Ar), 6.83 (d, *J* = 4.4 Hz, 1H, *H*C=N), 5.14–5.11 (m, 2H, 4-H and 3-H), 5.09 (s, 1H, 1-H), 4.65 (d, *J* = 12.0 Hz, 1H, OC*H*_2_Ar), 4.64–4.63 (m, 1H, 2-H), 4.43 (d, *J* = 12.0 Hz, 1H, OC*H*_2_Ar), 3.78 (t, *J* = 6.0 Hz, 2H, C*H*_*2*_N), 1.95–1.91 (m, 1H), 1.85–1.83 (m, 1H), 1.38 (s, 3H, C(C*H*_*3*_)_2_), 1.34–1.26 (m, 10H), 1.25 (s, 3H, C(C*H*_*3*_)_2_), 0.85 (t, *J* = 6.0 Hz, 3H) ppm. ^13^C{1H} NMR (100 MHz, CDCl_3_): δ 137.0 (Ar), 135.1 (H*C*=N), 128.5–127.9 (5C, Ar), 112.6 (*C*(CH_3_)_2_), 105.0 (C-1), 85.4 (C-2), 79.7 (C-3), 76.4 (C-4), 69.0 (O*C*H_2_Ar), 65.3 (*C*H_2_N), 31.8–22.7 (6C and 2C, C(*C*H_3_)_2_), 14.1 ppm. MS (ESI) *m*/*z* (%): 833.0 (100) [2M + Na]^+^, 428.23 (20) [M + Na]^+^. IR (CDCl_3_) ν: 970, 1028, 1101, 1161, 1209, 1267, 1356, 1456, 2232, 2928, 3034, 3066 cm^–1^. Anal. Calcd for C_23_H_35_NO_5_: C, 68.12; H, 8.70; N, 3.45. Found: C, 68.34; H, 8.40; N, 3.50.

#### 4.1.8 Synthesis of (*Z*)-Benzyl 2,3-*O*-(1-methylethylidene)-5-deoxy-*N*-dodecyl-d-lyxofuranosylamine *N*-Oxide (**10f**)

Application of the general procedure to 200 mg (0.72 mmol) of **8** with 160 mg (0.86 mmol) of dodecylamine (**13f**) furnished, after purification by column chromatography (PEt/EtOAc 2:1), 233 mg (0.50 mmol, 70%) of **10f** as a white solid (R_*f*_ = 0.31, PEt/EtOAc 2:1).

**10f**: white solid. mp 95–97 °C. [α]_D_^20^ – 3.2 (*c* 0.81, CHCl_3_). ^1^H NMR (400 MHz, CDCl_3_): δ 7.34–7.26 (m, 5H, Ar), 6.86 (d, *J* = 4.8 Hz, 1H, *H*C=N), 5.18–5.14 (m, 2H, 4-H and 3-H), 5.13 (s, 1H, 1-H), 4.70 (d, *J* = 12.0 Hz, 1H, OC*H*_2_Ar), 4.69–4.66 (m, 1H, 2-H), 4.47 (d, *J* = 12.0 Hz, 1H, OC*H*_2_Ar), 3.82 (t, *J* = 7.0 Hz, 2H, C*H*_*2*_N), 1.98–1.95 (m, 1H), 1.88–1.85 (m, 1H), 1.41 (s, 3H), 1.32–1.26 (m, 21H), 0.88 (t, *J* = 6.6 Hz, 3H) ppm. ^13^C{1H} NMR (100 MHz, CDCl_3_): δ 137.1 (Ar), 135.2 (H*C*=N), 128.6–128.0 (5C, Ar), 112.7 (*C*(CH_3_)_2_), 105.1 (C-1), 84.7 (C-2), 79.8 (C-3), 76.5 (C-4), 69.1 (O*C*H_2_Ar), 65.5 (*C*H_2_N), 32.0–24.8 (10C and 2C, C(*C*H_3_)_2_), 14.2 ppm. MS (ESI) *m*/*z* (%): 944.73 (100) [2M + Na]^+^, 484.32 (30) [M + Na]^+^. IR (CDCl_3_) ν: 972, 1026, 1087, 1161, 1211, 1263, 1356, 1456, 2232, 2928, 3034 cm^–1^. Anal. Calcd for C_27_H_43_NO_5_: C, 70.25; H, 9.39; N, 3.03. Found: C, 70.35; H, 9.25; N, 3.00.

#### 4.1.9 Synthesis of (*Z*)-Benzyl 2,3-*O*-(1-methylethylidene)-5-deoxy-*N*-isopropyl-d-lyxofuranosylamine *N*-Oxide (**10g**)

Application of the general procedure to 200 mg (0.72 mmol) of **8** with 74 μL (0.86 mmol) of isopropylamine (**13g**) furnished, after purification by column chromatography (Hex/EtOAc 2:1), 157 mg (0.47 mmol, 65%) of **10g** as a colorless oil (R_*f*_ = 0.20, Hex/EtOAc 2:1).

**10g**: colorless oil. [α]_D_^24^ – 15.9 (*c* 1.00, CHCl_3_). ^1^H NMR (400 MHz, CDCl_3_): δ 7.36–7.26 (m, 5H, Ar), 6.93 (d, *J* = 6.8 Hz, 1H, *H*C=N), 5.20–5.13 (m, 2H, 4-H and 3-H), 5.12 (s, 1H, 1-H), 4.71 (d, *J* = 12.0 Hz, 1H, OC*H*_2_Ar), 4.70–4.67 (m, 1H, 2-H), 4.46 (d, *J* = 12.0 Hz, 1H, OC*H*_2_Ar), 4.13 (sept, *J* = 6.0 Hz, 1H, C*H*(CH_3_)_2_), 1.47–1.43 (m, 6H, −CH(C*H*_3_)_2_), 1.41 (s, 3H, C(C*H*_*3*_)_2_), 1.28 (s, 3H, C(C*H*_*3*_)_2_) ppm. ^13^C{1H} NMR (50 MHz, CDCl_3_): δ 137.0 (Ar), 132.8 (H*C*=N), 128.6–128.1 (5C, Ar), 112.7 (*C*(CH_3_)_2_), 105.0 (C-1), 84.6 (C-2), 79.8 (C-3), 76.3 (C-4), 69.1 (O*C*H_2_Ar), 66.3 (*C*H(CH_3_)_2_), 26.2 (C(*C*H_3_)_2_), 24.8 (C(*C*H_3_)_2_), 21.2 (CH(*C*H_3_)_2_), 20.7 (CH(*C*H_3_)_2_) ppm. MS (ESI) *m*/*z* (%): 358.12 (100) [M + Na]^+^. IR (CDCl_3_) ν: 972, 1082, 1161, 1377, 1456, 1737, 2234, 2875, 2933, 2985, 3606, 3689 cm^–1^. Anal. Calcd for C_18_H_25_NO_5_: C, 64.46; H, 7.51; N, 4.18. Found: C, 64.50; H, 7.71; N, 3.98.

#### 4.1.10 Synthesis of (*Z*)-Benzyl 2,3-*O*-(1-methylethylidene)-5-deoxy-*N*-cyclohexyl-d-lyxofuranosylamine *N*-Oxide (**10h**)

Application of the general procedure to 200 mg (0.72 mmol) of **8** with 99 μL (0.86 mmol) of cyclohexylamine (**13h**) furnished, after purification by column chromatography (Hex/EtOAc 2:1), 162 mg (0.43 mmol, 60%) of **10h** as a white solid (R_*f*_ = 0.30, Hex/EtOAc 2:1).

**10h**: white solid. mp 113–115 °C. [α]_D_^24^ – 11.1 (*c* 1.00, CHCl_3_). ^1^H NMR (400 MHz, CDCl_3_): δ 7.36–7.25 (m, 5H, Ar), 6.90 (d, *J* = 8.0 Hz, 1H, *H*C=N), 5.19 (pseudo t, *J* = 4.2 Hz, 1H, 4-H), 5.14–5.12 (m, 2H, 1-H and 3-H), 4.70 (d, *J* = 12.0 Hz, 1H, OC*H*_2_Ar), 4.67 (dd, *J* = 6.0, 1.2 Hz, 1H, 2-H), 4.46 (d, *J* = 12.0 Hz, 1H, OC*H*_2_Ar), 3.78–3.72 (m, 1H, C*H*N), 2.09–2.06 (m, 2H), 1.91–1.77 (m, 4H), 1.70–1.67 (m, 2H), 1.41 (s, 3H, C(C*H*_*3*_)_2_), 1.28 (s, 3H, C(C*H*_*3*_)_2_), 1.36–1.20 (m, 3H) ppm. ^13^C{1H} NMR (50 MHz, CDCl_3_): δ 137.1 (Ar), 133.0 (H*C*=N), 128.6–128.1 (5C, Ar), 112.7 (*C*(CH_3_)_2_), 105.0 (C-1), 84.7 (C-2), 79.8 (C-3), 76.4 (C-4), 73.9 (*C*HN), 69.0 (O*C*H_2_Ar), 31.4–31.0 (2C), 26.2–24.8 (3C, and 2C, C(*C*H_3_)_2_) ppm. MS (ESI) *m*/*z* (%): 772.64 (100) [2M + Na]^+^, 398.13 (98) [M + Na]^+^. IR (CDCl_3_) ν: 968, 1026, 1088, 1161, 1211, 1359, 1382, 1454, 2231, 2939 cm^–1^. Anal. Calcd for C_21_H_29_NO_5_: C, 67.18; H, 7.79; N, 3.73. Found: C, 67.00; H, 7.80; N, 3.75.

#### 4.1.11 Synthesis of (3*R*,4*S*,5*R*)-5-Hydroxy-3,4-*O*-(1-methylethylidene)-*N*-octyl-piperidine (**16**)^11^

To a mixture nitrone **10a** (75 mg, 0.18 mmol) in dry MeOH (16 mL), acetic acid (2 equivalents) and Pd/C (40 mg) were added under nitrogen atmosphere. The mixture was stirred at room temperature under hydrogen atmosphere (balloon) for 2 days, until a control by ^1^H NMR spectroscopy attested the presence of the acetate salt of (3*R*,4*S*,5*R*)-3-hydroxy-4,5-*O*-(1-methylethylidene)-*N*-octyl-piperidine (**16**). The mixture was filtered through Celite, and the solvent was removed under reduced pressure. The corresponding free amine was obtained by dissolving the residue in MeOH; then, the strongly basic resin Ambersep 900-OH was added, and the mixture was stirred for 40 min. The resin was removed by filtration, and the solvent evaporated to afford compound **16** (41 mg, 0.14 mmol) as a white solid in 80% yield.

**16**: white solid. ^1^H NMR (400 MHz, CDCl_3_): δ 4.27 (dt, *J* = 7.6, 5.1 Hz, 1H), 4.08 (dd, *J* = 4.9, 3.9 Hz, 1H), 3.95 (dd, *J* = 7.6, 3.8 Hz, 1H), 2.82 (ddd, *J* = 7.7, 6.3, 1.5 Hz, 1H), 2.56 (d, *J* = 2.4 Hz, 2H), 2.43–2.34 (m, 3H), 1.50 (s, 3H), 1.48–1.41 (m, 4H) 1.35 (s, 3H), 1.33–1.20 (m, 8H), 0.88 (t, *J* = 6.8 Hz, 3H) ppm.

#### 4.1.12 Synthesis of (3*R*,4*S*,5*R*)-5-Hydroxy-3,4-*O*-(1-methylethylidene)-*N*-dodecyl-piperidine (**17**)

To a mixture of nitrone **10b** (65 mg, 0.14 mmol) in dry MeOH (15 mL), Pd(OH)_2_ (40 mg) was added under nitrogen atmosphere. The mixture was stirred at room temperature under hydrogen atmosphere (balloon) for 2 days, until a control by ^1^H NMR spectroscopy attested the presence of (3*R*,4*S*,5*R*)-3-hydroxy-4,5-*O*-(1-methylethylidene) -*N*-dodecyl-piperidine (**17**). The mixture was filtered through Celite, and the solvent was removed under reduced pressure to afford compound **17** (38 mg, 0.13 mmol) as a colorless waxy solid in 90% yield.

**17**: colorless waxy solid. [α]_D_^22^ + 12.0 (*c* 0.31 in CHCl_3_). ^1^H NMR (400 MHz, CDCl_3_): δ 4.31 (pseudo q, *J* = 6.2 Hz, 1H, 3-H), 4.08 (t, *J* = 4.4 Hz, 1H, 4-H), 3.97–3.94 (m, 1H, 5-H), 2.82 (dd, *J* = 12.0, 6.0 Hz, 1H, 2-Ha), 2.58 (d, *J* = 2.9 Hz, 2H, 6-H), 2.42–2.35 (m, 3H, 2-Hb and C*H*_*2*_N), 1.60 (br s, OH), 1.51 (s, 3H, C(C*H*_*3*_)_2_), 1.50–1.46 (m, 2H), 1.36 (s, 3H, C(C*H*_*3*_)_2_), 1.31–1.25 (m, 18H), 0.87 (t, *J* = 6.8 Hz, 3H) ppm. ^13^C{1H} NMR (50 MHz, CDCl_3_): δ 109.5 (*C*(CH_3_)_2_), 77.3 (C-4), 72.4 (C-3), 67.8 (C-5), 58.1 (*C*H_2_N), 56.1 (C-2), 55.8 (C-6), 32.1–22.9 (10C and 2C, C(*C*H_3_)_2_), 14.3 ppm. MS (ESI) *m*/*z* (%): 342.33 (100) [M + H]^+^. IR (CHCl_3_) ν: 3458, 2928, 2854, 1468, 1456, 1404, 1383, 1246, 1144 cm^–1^. Anal. Calcd for C_20_H_39_NO_3_: C, 70.33; H, 11.51; N, 4.10. Found: C, 70.51; H, 11.72, N, 4.22.

#### 4.1.13 Synthesis of (3*R*,4*S*,5*R*)-5-Hydroxy-3,4-*O*-(1-methylethylidene)-*N*-Cyclohexyl-piperidine (**18**)

To a solution of nitrone **10d** (117 mg, 0.31 mmol) in dry MeOH (30 mL), Pd(OH)_2_ (70 mg) was added under nitrogen atmosphere. The mixture was stirred at room temperature under hydrogen atmosphere (balloon) for 2 days, until a control by ^1^H NMR spectroscopy attested the presence of (3*R*,4*S*,5*R*)-5-hydroxy-3,4-*O*-(1-methylethylidene)-*N*-cyclohexylpiperidine (**18**). The mixture was filtered through Celite, and the solvent was removed under reduced pressure to afford compound **18** (67 mg, 0.26 mmol) as a colorless oil in 85% yield.

**18**: colorless oil. [α]_D_^24^ + 22.9 (*c* 1.00, CHCl_3_). ^1^H NMR (400 MHz, CD_3_OD): δ 4.31–4.29 (m, 1H, 3-H), 3.87–3.84 (m, 1H, 4-H), 3.83–3.78 (m, 1H, 5-H), 3.04 (d, *J* = 12.0 Hz, 1H, 2-H_a_), 2.83 (d, *J* = 12.0 Hz, 1H, 6-H_a_), 2.76 (d, *J* = 12.0 Hz, 1H, 2-H_b_), 2.52–2.41 (m, 1H, C*H*N), 2.30–2.25 (m, 1H, 6-H_b_), 1.91–1.89 (m, 2H), 1.85–1.82 (m, 2H), 1.68–1.65 (m, 1H), 1.50 (s, 3H, C(C*H*_*3*_)_2_), 1.34 (s, 3H, C(C*H*_*3*_)_2_), 1.43–1.10 (m, 5H) ppm. ^13^C{1H} NMR (100 MHz, CD_3_OD): δ 110.2 (*C*(CH_3_)_2_), 80.0 (C-4), 74.4 (C-3), 70.5 (C-5), 64.8 (*C*HN), 53.3 (C-6), 50.7 (C-2), 29.6–26.5 (5C, and 2C, C(*C*H_3_)_2_) ppm. MS (ESI) *m*/*z* (%): 256.16 (100) [M + H]^+^, 278.11 (36) [2M + Na]^+^. IR (CD_3_OD) ν: 1073, 1110, 1220, 1380, 2065, 2640, 2858, 2930, 3340 cm^–1^. Anal. Calcd for C_14_H_25_NO_3_: C, 65.85; H, 9.87; N, 5.49. Found: C, 65.87; H, 9.77; N, 5.53.

#### 4.1.14 Synthesis of (3*R*,4*S*,5*R*)-5-Hydroxy-3,4-*O*-(1-methylethylidene)-piperidine (**19**)^11^

To a solution of nitrone **10e** (915 mg, 2.39 mmol) in dry MeOH (150 mL), acetic acid (2 equivalents) and Pd/C (458 mg) were added under nitrogen atmosphere. The mixture was stirred at room temperature under hydrogen atmosphere (balloon) for 2 days, until a control by ^1^H NMR spectroscopy attested the presence of the acetate salt of (3*R*,4*S*,5*R*)-5-hydroxy-3,4-*O*-(1-methylethylidene)-piperidine (**19**). The mixture was filtered through Celite, and the solvent was removed under reduced pressure. The corresponding free amine was obtained by dissolving the residue in MeOH; then, the strongly basic resin Ambersep 900-OH was added, and the mixture was stirred for 40 min. The resin was removed by filtration, and the solvent evaporated under reduced pressure to afford (3*R*,4*S*,5*R*)-5-hydroxy-3,4-*O*-(1-methylethylidene)-piperidine (**19**, 414 mg, 2.39 mmol) as a white solid in 100% yield.

**19**: white solid. ^1^H NMR (400 MHz, CD_3_OD): δ 4.26–4.16 (m, 1H), 3.91 (t, *J* = 6.1 Hz, 1H), 3.75–3.62 (m, 1H), 3.13 (dd, *J* = 14.5, 2.5 Hz, 1H), 3.01–2.94 (m, 1H), 2.94–2.86 (m, 1H), 2.38 (dd, *J* = 13.1, 9.2 Hz, 1H), 1.50 (s, 3H), 1.35 (s, 3H) ppm.

#### 4.1.15 Synthesis of Hydroxylamines: (5*S*)-Benzyl-2,3-*O*-(1-methylethylidene)-5-deoxy-5-octyl-d-lyxofuranosyl *N*-octyl-hydroxylamine (**21**) and (5*R*)-Benzyl-2,3-*O*-(1-methylethylidene)-5-deoxy-5-octyl-d-lyxofuranosyl *N*-octyl-hydroxylamine (**22**)

##### 4.1.15.1 Procedure without Lewis Acid

A solution of nitrone **10a** in dry THF (0.03 M) was stirred at −78 °C under nitrogen atmosphere and 2.0 M solution of octylmagnesium bromide in diethyl ether (1.13 mmol, 600 μL) was slowly added. The reaction mixture was stirred at −78 °C under nitrogen atmosphere for 3 h, when a TLC control (Hex/EtOAc 2:1) attested the disappearance of the starting material. A saturated ammonium chloride solution (10 mL) and Et_2_O (10 mL) were added to the mixture at 0 °C and stirred for 15 min. The two layers were separated, and the aqueous layer was extracted with Et_2_O (2 × 10 mL). The combined organic layers were washed with brine (2 × 30 mL), dried with Na_2_SO_4_, and concentrated under reduced pressure to give a mixture of hydroxylamines **21** and **22** (**21** > 98%). The crude mixture was purified by silica gel column chromatography (gradient eluent from Hex/EtOAc 12:1 to 10:1) to give 246 mg (0.47 mmol, 75%) of **21** (R_*f*_ = 0.36, Hex/EtOAc 12:1) as a colorless oil.

**21**: colorless oil. ^1^H NMR (400 MHz, CDCl_3_): δ 7.37–7.26 (m, 5H, Ar), 5.13 (s, 1H, 1-H), 5.11 (br s, OH), 4.69–4.66 (m, 2H, 3-H and OC*H*_2_Ar), 4.61 (d, *J* = 4.0 Hz, 1H, 2-H), 4.52 (d, *J* = 12.0 Hz, 1H, OC*H*_2_Ar), 4.24 (dd, *J* = 9.2, 3.0 Hz, 1H, 4-H), 3.31–3.26 (m, 1H, 5-H), 2.91–2.86 (m, 1H, C*H*_2_N), 2.77–2.72 (m, 1H, C*H*_2_N), 1.64–1.54 (m, 2H), 1.45–1.27 (m, 30H), 0.88 (t, *J* = 8.0 Hz, 6H) ppm. MS (ESI) *m*/*z* (%): 542.45 (100) [M + Na]^+^, 520.37 (34) [M + H]^+^.

The secondary hydroxylamine **21** spontaneously oxidizes to the corresponding nitrone **23** so we could only perform ^1^H NMR and MS-ESI spectra immediately after their purification by column chromatography.

##### 4.1.15.2 Procedure with Lewis Acid

To a stirred solution of nitrone **10a** in dry THF (0.03 M) at room temperature, boron trifluoride diethyl etherate (0.82 mmol, 100 μL) was added, and the resulting mixture was stirred at room temperature under nitrogen atmosphere for 15 min. The reaction mixture was cooled at −30 °C and 2.0 M solution of octylmagnesium bromide in diethyl ether (1.48 mmol, 800 μL) was slowly added. The reaction mixture was stirred at −30 °C under nitrogen atmosphere for 2 h, when a TLC control (Hex/EtOAc 2:1) attested the disappearance of the starting material. A saturated ammonium chloride solution (10 mL) and Et_2_O (10 mL) were added to the mixture at 0 °C and stirred for 20 min. The two layers were separated, and the aqueous layer was extracted with Et_2_O (2 × 10 mL). The combined organic layers were washed with brine (2 × 30 mL), dried with Na_2_SO_4_, and concentrated under reduced pressure to give a mixture of hydroxylamines **21** and **22** (**22** > 98%). The crude mixture was purified by silica gel column chromatography (gradient eluent from Hex/EtOAc 12:1 to 10:1) to give 298 mg (0.57 mmol, 70%) of **22** (R_*f*_ = 0.20, Hex/EtOAc 12:1) as a colorless oil.

**22**: colorless oil. ^1^H NMR (400 MHz, CDCl_3_): δ 7.34–7.25 (m, 5H, Ar), 6.54 (br s, OH), 5.07 (s, 1H, 1-H), 4.77–4.76 (m, 1H, 3-H), 4.66 (d, *J* = 12.0 Hz, 1H, OC*H*_2_Ar), 4.61 (d, *J* = 4.0 Hz, 1H, 2-H), 4.48 (d, *J* = 12.0 Hz, 1H, OC*H*_2_Ar), 4.32–4.29 (m, 1H, 4-H), 3.30 (q, *J* = 8.0 Hz, 1H, 5-H), 2.77–2.69 (m, 2H, C*H*_*2*_N), 1.65–1.58 (m, 2H), 1.48–1.28 (m, 30H), 0.89 (t, *J* = 6.0 Hz, 6H) ppm. MS (ESI) *m*/*z* (%): 520.36 (100) [M + H]^+^, 542.31 (46) [M + Na]^+^.

The secondary hydroxylamine **22** spontaneously oxidizes to the corresponding nitrone **24**, so we could only perform the ^1^H NMR and MS-ESI spectra immediately after their purification by column chromatography.

#### 4.1.16 Synthesis of (5*S,Z*)-Benzyl-2,3-*O*-(1-methylethylidene)-5-deoxy-5-octyl-d-lyxofuranosyl 5-(*N*-octylidene *N*-oxide) (**23**)

To a stirred solution of hydroxylamine **21** (213 mg, 0.41 mmol) in dry CH_2_Cl_2_ (6 mL), IBX [2-iodoxybenzoic acid contains stabilizer (45 wt %)] (382 mg, 0.62 mmol) was added, and the resulting mixture was stirred under nitrogen atmosphere at room temperature for 3 h, when a TLC control (Hex/EtOAc 12:1) attested the disappearance of the starting material. Saturated solution of NaHCO_3_ (10 mL) was added, and the aqueous layer was extracted with CH_2_Cl_2_ (3 × 10 mL). The combined organic layers were washed with brine (2 × 15 mL) and evaporated under reduced pressure after drying with Na_2_SO_4_. The residue was purified by silica gel flash column chromatography (Hex/EtOAc from 2:1) to give 202 mg (0.39 mmol, 95%) of nitrone **23** (R_*f*_ = 0.34, Hex/EtOAc from 2:1) as a straw yellow oil.

**23**: straw yellow oil. [α]_D_^24^ + 80.0 (*c* 0.80, CHCl_3_). ^1^H NMR (400 MHz, CDCl_3_): δ 7.32–7.26 (m, 5H, Ar), 6.71 (t, *J* = 6.0 Hz, 1H, *H*C=N), 5.00 (s, 1H, 1-H), 4.71–4.70 (m, 1H, 3-H), 4.65–4.62 (m, 3H, 2-H and OC*H*_2_Ar), 4.53 (dd, *J* = 8.0, 4.0 Hz, 1H, 4-H), 4.39 (d, *J* = 12.0 Hz, 1H, OC*H*_2_Ar), 3.83 (t, *J* = 8.0 Hz, 1H, 5-H), 2.55 (q, *J* = 6.7 Hz, 2H, C*H*_*2*_CHN), 2.10–2.02 (m, 1H), 1.60–1.54 (m, 3H), 1.45 (s, 3H, C(C*H*_*3*_)_2_), 1.40–1.25 (m, 23H), 0.89–0.84 (m, 6H) ppm. ^13^C{1H} NMR (50 MHz, CDCl_3_): δ 139.8 (H*C*=N), 137.4 (Ar), 128.6–127.9 (5C, Ar), 112.8 (*C*(CH_3_)_2_), 104.5 (C-1), 85.4 (C-2), 79.7 (C-3), 78.8 (C-4), 75.1 (C-5), 68.7 (O*C*H_2_Ar), 32.0–22.8 (13C, and 2C, C(*C*H_3_)_2_), 14.2 (2C) ppm. MS (ESI) *m*/*z* (%): 1057.16 (100) [2M + Na]^+^, 540.43 (23) [M + Na]^+^. IR (CDCl_3_) ν: 957, 1080, 1107, 1161, 1209, 1496, 1595, 2857, 2928, 3032, 3067 cm^–1^. Anal. Calcd for C_31_H_51_NO_5_: C, 71.92; H, 9.93; N, 2.71. Found: C, 71.90; H, 10.05; N, 3.01.

#### 4.1.17 Synthesis of (5*R,Z*)-Benzyl-2,3-*O*-(1-methylethylidene)-5-deoxy-5-octyl-d-lyxofuranosyl 5-(*N*-Octylidene *N*-oxide) (**24**)

To a stirred solution of hydroxylamine **22** (150 mg, 0.29 mmol) in dry CH_2_Cl_2_ (4.5 mL), IBX [2-iodoxybenzoic acid contains stabilizer (45 wt %)] (273 mg, 0.44 mmol) was added, and the resulting mixture was stirred under nitrogen atmosphere at room temperature for 3 h, when a TLC control (Hex/EtOAc 12:1) attested the disappearance of the starting material. Saturated solution of NaHCO_3_ (8 mL) was added, and the aqueous layer was extracted with CH_2_Cl_2_ (3 × 8 mL). The combined organic layers were washed with brine (2 × 13 mL) and evaporated under reduced pressure after drying with Na_2_SO_4_. The residue was purified by silica gel flash column chromatography (Hex/EtOAc from 2:1) to give 140 mg (0.27 mmol, 93%) of nitrone **24** (R_*f*_ = 0.35, Hex/EtOAc from 2:1) as a straw yellow oil.

**24**: straw yellow oil. [α]_D_^24^ – 47.5 (*c* 0.72, CHCl_3_). ^1^H NMR (400 MHz, CDCl_3_): δ 7.35–7.26 (m, 5H, Ar), 6.71 (t, *J* = 6.0 Hz, 1H, *H*C=N), 5.03 (s, 1H, 1-H), 4.69–4.67 (m, 1H, 3-H), 4.64 (d, *J* = 12.0 Hz, 1H, OC*H*_2_Ar), 4.60 (d, *J* = 8.0 Hz, 1H, 2-H), 4.48 (d, *J* = 12.0 Hz, 1H, OC*H*_2_Ar), 4.40 (dd, *J* = 8.0, 4.0 Hz, 1H, 4-H), 3.96–3.90 (m, 1H, 5-H), 2.60–2.41 (m, 2H, C*H*_*2*_CHN), 2.10–2.01 (m, 1H), 1.75–1.69 (m, 1H), 1.59–1.49 (m, 2H), 1.43 (s, 3H, C(C*H*_*3*_)_2_), 1.39–1.24 (m, 23H), 0.87 (t, *J* = 8 Hz, 6H) ppm. ^13^C{1H} NMR (100 MHz, CDCl_3_): δ 141.9 (H*C*=N), 137.4 (Ar), 128.6–127.9 (5C, Ar), 112.4 (*C*(CH_3_)_2_), 105.5 (C-1), 85.3 (C-2), 79.9 (C-4), 79.4 (C-3), 73.2 (C-5), 69.2 (O*C*H_2_Ar), 32.0–22.7 (13C and 2C, C(*C*H_3_)_2_), 14.2 (2C) ppm. MS (ESI) *m*/*z* (%): 1057.02 (100) [2M + Na]^+^, 540.42 (32) [M + Na]^+^. IR (CDCl_3_) ν: 961, 1012, 1080, 1161, 1209, 1261, 1496, 1597, 2237, 2856, 2927, 3032, 3066 cm^–1^. Anal. Calcd for C_31_H_51_NO_5_ (517.38): C, 71.92; H, 9.93; N, 2.71. Found: C, 71.80; H, 10.00; N, 2.63.

#### 4.1.18 Synthesis of (2*S*,3*R*,4*S*,5*R*)-3-Hydroxy-4,5-*O*-(1-methylethylidene)-2-octyl-*N*-octyl-piperidine (**25**)

To a mixture of nitrone **23** and hydroxylamine **21** in dry MeOH (0.015 M), acetic acid (2 equivalents) and Pd/C (107 mg) were added under nitrogen atmosphere. The mixture was stirred at room temperature under hydrogen atmosphere (balloon) for 2 days, until a control by ^1^H NMR spectroscopy attested the presence of the acetate salt of compound **25**. The mixture was filtered through Celite, and the solvent was removed under reduced pressure. The corresponding free amine was obtained by dissolving the residue in MeOH; then, the strongly basic resin Ambersep 900-OH was added, and the mixture was stirred for 40 min. The resin was removed by filtration, and the crude product was purified on silica gel by flash column chromatography (CH_2_Cl_2_/MeOH/NH_4_OH (6%) 10:1:0.1) to afford 150 mg (0.38 mmol, 90%) of **25** (R_*f*_ = 0.50, CH_2_Cl_2_/MeOH/NH_4_OH (6%) 10:1:0.1) as a white solid.

**25**: white solid. mp 50–52 °C. [α]_D_^25^ + 22.7 (*c* 0.77, CHCl_3_). ^1^H NMR (400 MHz, CD_3_OD): δ 4.26–4.23 (m, 1H, 5-H), 4.04 (t, *J* = 6.0 Hz, 1H, 4-H), 3.87 (dd, *J* = 6.4, 4.0 Hz, 1H, 3-H), 2.94 (dd, *J* = 14.0, 4.0 Hz, 1H, 6-H_a_), 2.82 (dd, *J* = 14.0, 3.9 Hz, 1H, 6-H_b_), 2.75–2.68 (m, 2H, 2-H and C*H*_2_N), 2.64–2.57 (m, 1H, C*H*_2_N), 1.55–1.43 (m, 3H), 1.47 (s, 3H, C(C*H*_*3*_)_2_), 1.38–1.32 (m, 26H), 0.91 (t, *J* = 6.0 Hz, 6H) ppm. ^13^C{1H} NMR (50 MHz, CD_3_OD): δ 109.8 (*C*(CH_3_)_2_), 78.3 (C-4), 74.6 (C-5), 70.1 (C-3), 61.8 (C-2), 55.8 (*C*H_2_N), 49.2 (C-6), 30.7–23.7 (13C and 2C, C(*C*H_3_)_2_), 14.4 (2C) ppm. MS (ESI) *m*/*z* (%): 817.04 (100) [2M + Na]^+^, 398.43 (75) [M + H]^+^. IR (CD_3_OD) ν: 1072, 1109, 1219, 1246, 1379, 1462, 2250, 2295, 2637, 2859, 2930, 3339 cm^–1^. Anal. Calcd for C_24_H_47_NO_3_: C, 72.49; H, 11.91; N, 3.52. Found: C, 72.50; H, 12.05; N, 3.85.

#### 4.1.19 Synthesis (2*R*,3*R*,4*S*,5*R*)-3-Hydroxy-4,5-*O*-(1-methylethylidene)-2-octyl-*N*-octyl-piperidine (**26**)

To a mixture of nitrone **24** and hydroxylamine **22** in dry MeOH (0.015 M), acetic acid (2 equivalents) and Pd/C (125 mg) were added under nitrogen atmosphere. The mixture was stirred at room temperature under hydrogen atmosphere (balloon) for 2 days, until a control by ^1^H NMR spectroscopy attested the presence of the acetate salt of compound **26**. The mixture was filtered through Celite, and the solvent was removed under reduced pressure. The corresponding free amine was obtained by dissolving the residue in MeOH; then, the strongly basic resin Ambersep 900-OH was added, and the mixture was stirred for 40 min. The resin was removed by filtration, and the crude product was purified on silica gel by flash column chromatography (CH_2_Cl_2_/MeOH/NH_4_OH (6%) 10:1:0.1) to afford 150 mg (0.38 mmol, 80%) of **26** (R_*f*_ = 0.50, CH_2_Cl_2_/MeOH/NH_4_OH (6%) 10:1:0.1) as a straw yellow oil.

**26**: straw yellow oil. [α]_D_^25^ – 27.0 (*c* 0.92, CHCl_3_). ^1^H NMR (400 MHz, CD_3_OD): δ 4.28 (q, *J* = 4.6 Hz, 1H, 5-H), 3.90 (t, *J* = 6.0 Hz, 1H, 4-H), 3.58 (t, *J* = 7.5 Hz, 1H, 3-H), 3.08 (dd, *J* = 13.6, 3.8 Hz, 1H, 6-H_a_), 2.71–2.63 (m, 2H, 6-H_b_ and C*H*_2_N), 2.46–2.39 (m, 1H, C*H*_2_N), 2.24–2.19 (m, 1H, 2-H), 1.71–1.63 (m, 1H), 1.60–1.53 (m, 1H), 1.47 (s, 3H, C(C*H*_*3*_)_2_), 1.44–1.32 (m, 27H)), 0.91 (t, *J* = 6.0 Hz, 6H) ppm. ^13^C{1H} NMR (100 MHz, CD_3_OD): δ 110.0 (*C*(CH_3_)_2_), 80.7 (C-4), 74.2 (C-5), 72.0 (C-3), 64.2 (C-2), 53.3 (*C*H_2_N), 51.3 (C-6), 33.1–23.7 (13C and 2C, C(*C*H_3_)_2_), 14.4 (2C) ppm. 1D-NOESY: irradiation of 2-H gave a NOE at 4-H and 6-H_b_, and irradiation of 4-H gave a NOE at 2-H and 6-H_b_. MS (ESI) *m*/*z* (%): 398.40 (100) [M + H]^+^. IR (CD_3_OD) ν: 1084, 1117, 1219, 1246, 1379, 1464, 2293, 2640, 2857, 2930, 3343 cm^–1^. Anal. Calcd for C_24_H_47_NO_3_: C, 72.49; H, 11.91; N, 3.52. Found: C, 72.40; H, 11.89; N, 3.50.

#### 4.1.20 Synthesis of (2*S*,3*R*,4*R*,5*R*)-1,2-Dioctylpiperidine-3,4,5-triol (**14**)

A solution of **25** (150 mg, 0.38 mmol) in MeOH (24 mL) was stirred with 12 M HCl (750 μL) at room temperature for 16 h. The crude mixture was concentrated to yield the hydrochloride salt of compound **14**. The corresponding free amine was obtained by dissolving the residue in MeOH; then, the strongly basic resin Ambersep 900-OH was added, and the mixture was stirred for 40 min. The resin was removed by filtration, and the solvent evaporated to afford 134 mg (0.38 mmol, 100%) of **14** as an orange oil.

**14**: orange oil. [α]_D_^25^ + 11.9 (*c* 0.91, CH_3_OH). ^1^H NMR (400 MHz, CD_3_OD): δ 3.95–3.93 (m, 1H, 5-H), 3.77–3.74 (m, 2H, 4-H and 3-H), 2.67–2.53 (m, 5H, 2-H, 6-H and C*H*_*2*_N), 1.55–1.51 (m, 2H), 1.49–1.31 (m, 24H), 0.90 (t, *J* = 6.0 Hz, 6H) ppm. ^13^C{1H} NMR (50 MHz, CD_3_OD): δ 72.5 (C-4), 71.3 (C-3), 67.4 (C-5), 59.4 (C-2), 54.2 (*C*H_2_N), 53.2 (C-6), 33.1–23.7 (13C), 14.4 (2C) ppm. MS (ESI) *m*/*z* (%): 358.42 (100) [M + H]^+^. Anal. Calcd for C_21_H_43_NO_3_: C, 70.54; H, 12.12; N, 3.92. Found: C, 70.54; H, 12.15; N, 4.10.

#### 4.1.21 Synthesis of (2*R*,3*R*,4*R*,5*R*)-1,2-Dioctylpiperidine-3,4,5-triol (**15**)

A solution of **26** (131 mg, 0.32 mmol) in MeOH (20 mL) was stirred with 12 M HCl (650 μL) at room temperature for 16 h. The crude mixture was concentrated to yield the hydrochloride salt of compound **15**. The corresponding free amine was obtained by dissolving the residue in MeOH; then, the strongly basic resin Ambersep 900-OH was added, and the mixture was stirred for 40 min. The resin was removed by filtration, and the solvent evaporated to afford 107 mg (0.30 mmol, 90%) of **15** as a yellow oil.

**15**: yellow oil. [α]_D_^25^ – 14.9 (*c* 0.84, CH_3_OH). ^1^H NMR (400 MHz, CD_3_OD): δ 3.84 (br s, 1H, 5-H), 3.53 (t, *J* = 8.0 Hz, 1H, 3-H), 3.29–3.24 (m, 1H, 4-H), 2.98 (dd, *J* = 12.0, 4.0 Hz, 1H, 6-H_a_), 2.71–2.65 (m, 1H, C*H*_2_N), 2.39–2.32 (m, 2H, 6-H_b_ and C*H*_2_N), 2.08–2.06 (m, 1H, 2-H),1.78–1.73 (m, 1H), 1.69–1.62 (m, 1H), 1.50–1.32 (m, 24H), 0.90 (t, *J* = 6.0 Hz, 6H) ppm. ^13^C{1H} NMR (100 MHz, CD_3_OD): δ 76.6 (C-4), 71.5 (C-3), 69.5 (C-5), 66.0 (C-2), 54.2 (C-6), 53.2 (*C*H_2_N), 33.1–23.8 (13C), 14.5 (2C) ppm. MS (ESI) *m*/*z* (%): 358.42 (100) [M + H]^+^. Anal. Calcd for C_21_H_43_NO_3_: C, 70.54; H, 12.12; N, 3.92. Found: C, 70.35; H, 11.99; N, 4.02.

#### 4.1.22 Synthesis of Benzyl-2,3-*O*-(1-methylethylidene)-α-d-lyxofuranosyl -5-(octyl)-5-(*N*-pentadecan-8-amine) (**31**)

To a stirred solution of nitrone **24** (190 mg, 0.37 mmol) in dry THF (7 mL) at room temperature, boron trifluoride diethyl etherate (0.37 mmol, 46 μL) was added and the resulting mixture was stirred at room temperature under nitrogen atmosphere for 15 min. The reaction mixture was cooled at −30 °C, and a solution of heptylmagnesium bromide in THF (0.74 mmol, 1.00 mL) was slowly added. The reaction mixture was stirred at room temperature under nitrogen atmosphere for 16 h, when a TLC control (Hex/EtOAc 2:1) attested the disappearance of the starting material. A 2:1 solution of EtOH and sat. aq. NH_4_Cl (3 mL) and indium powder (85 mg, 0.74 mmol) were added, and this mixture was heated to reflux (oil bath).

After completion of the reaction (TLC control), the mixture was cooled, filtered through Celite, and the solvent evaporated under reduced pressure. Then, a sat. aq. Na_2_CO_3_ solution (15 mL) was added, and the product was extracted with ethyl acetate (3 × 15 mL). The organic phase was dried over Na_2_SO_4_, then filtered, and the solvent evaporated under reduced pressure. The crude mixture was purified by silica gel column chromatography (gradient eluent CH_2_Cl_2_/MeOH/NH_4_OH (6%) 20:1:0.1) to give 100 mg (0.17 mmol, 50%) of **31** (R_*f*_ = 0.50, CH_2_Cl_2_/MeOH/NH_4_OH (6%) 20:1:0.1) as a colorless oil.

**31**: colorless oil. [α]_D_^24^ + 9.14 (*c* 1.20, CHCl_3_). ^1^H NMR (400 MHz, CDCl_3_): δ 7.34–7.23 (m, 5H, Ar), 5.04 (s, 1H, 1-H), 4.77–4.75 (m, 1H, 3-H), 4.66 (d, *J* = 12.0 Hz, 1H, OC*H*_2_Ar), 4.62–4.59 (m, 1H, 2-H), 4.46 (d, *J* = 12.0 Hz, 1H, OC*H*_2_Ar), 3.83–3.81 (m, 1H, 4-H), 3.06–3.05 (m, 1H, 5-H), 2.65 (br s, 1H, C*H*N), 1.62–1.27 (m, 44H), 0.89–0.87 (m, 9H) ppm. ^13^C{1H} NMR (50 MHz, CDCl_3_): δ 137.8 (Ar), 128.5–127.8 (5C, Ar), 112.2 (*C*(CH_3_)_2_), 105.3 (C-1), 85.3 (C-2), 82.4 (C-4), 80.1 (C-3), 68.9 (O*C*H_2_Ar), 54.8 (*C*HN), 52.9 (C-5), 32.1–22.8 (19C and 2C, C(*C*H_3_)_2_), 14.2 (3C) ppm. MS (ESI) *m*/*z* (%): 602.41 (100) [M + Na]^+^. IR (CDCl_3_) ν: 957, 1080, 1107, 1161, 1209, 1496, 1595, 2857, 2928, 3032, 3067 cm^–1^. Anal. Calcd for C_38_H_67_NO_4_: C, 75.82; H, 11.22; N, 2.33. Found: C, 76.01; H, 11.02; N, 2.02.

#### 4.1.23 Synthesis of (2*R*,3*R*,4*S*,5*R*)-3-Hydroxy-4,5-*O*-(1-methylethylidene)-2-octyl-*N*-pentadecan-8-yl-piperidine (**32**)

To a solution of amine **31** (100 mg, 0.17 mmol) in dry MeOH (20 mL), acetic acid (2 equivalents) and Pd/C (50 mg) were added under nitrogen atmosphere. The mixture was stirred at room temperature under hydrogen atmosphere (balloon) for 2 days, until a control by ^1^H NMR spectroscopy attested the presence of the acetate salt of compound **32**. The mixture was filtered through Celite, and the solvent was removed under reduced pressure. The corresponding free amine was obtained by dissolving the residue in MeOH; then, the strongly basic resin Ambersep 900-OH was added, and the mixture was stirred for 40 min. The resin was removed by filtration, and the solvent evaporated to afford **32** (46 mg, 0.093 mmol) as a straw yellow oil in 55% yield.

**32**: straw yellow oil. [α]_D_^24^ – 16.4 (*c* 0.40, CHCl_3_). ^1^H NMR (400 MHz, CD_3_OD): δ 4.23–4.21 (m, 1H, 5-H), 3.91 (t, *J* = 6.5 Hz, 1H, 4-H), 3.62 (t, *J* = 7.2 Hz, 1H, 3-H), 3.08 (dd, *J* = 14.0, 4.0 Hz, 1H, 6-H_a_), 2.62–2.59 (m, 2H, 6-H_b_ and C*H*N), 2.37 (br s, 1H, 2-H), 1.66–1.59 (m, 4H), 1.40 (s, 3H, C(C*H*_*3*_)_2_), 1.32–1.20 (m, 37H), 0.91 (t, *J* = 6.0 Hz, 9H) ppm. ^13^C{1H} NMR (100 MHz, CD_3_OD): δ 110.0 (s, *C*(CH_3_)_2_), 80.8 (C-4), 74.8 (C-5), 72.7 (C-3), 62.4 (C-2), 58.3 (*C*HN), 44.9 (C-6), 33.1–30.5 (19C and 2C, C(*C*H_3_)_2_), 14.4 (3C) ppm. MS (ESI) *m*/*z* (%): 496.20 (100) [M + H]^+^. IR (CD_3_OD) ν: 1084, 1246, 1380, 1463, 2293, 2640, 2857, 2930, 3342 cm^–1^. Anal. Calcd for C_31_H_61_NO_3_: C, 75.09; H, 12.40; N, 2.82. Found: C, 75.03; H, 12.76; N, 2.53.

#### 4.1.24 Synthesis of (2*R*,3*R*,4*R*,5*R*)-2-Octyl-1-(pentadecan-8-yl)-piperidine-3,4,5-triol Hydrochloride (**33·HCl**)

A solution of **32** (60 mg, 0.12 mmol) in MeOH (10 mL) was stirred with 12 M HCl (300 μL) at room temperature for 16 h. The crude mixture was concentrated to yield the hydrochloride salt of compound **33** (53 mg, 0.11 mmol) as a yellow waxy solid in 90% yield.

**33**·**HCl**: yellow waxy solid. [α]_D_^23^ – 31.4 (*c* 0.50, MeOH). ^1^H NMR (400 MHz, CD_3_OD): δ 4.14 (br s, 1H, 5-H), 3.81 (t, *J* = 9.0 Hz, 1H, 3-H), 3.56 (dd, *J* = 9.2, 3.2 Hz, 1H, 4-H), 3.42–3.38 (m, 2H, 6-H_a_ and C*H*N), 3.23 (d, *J* = 12.0 Hz, 1H, 6-H_b_), 3.18–3.16 (m, 1H, 2-H), 1.92–1.89 (m, 2H), 1.84–1.79 (m, 2H), 1.75–1.70 (m, 2H), 1.61–1.29 (m, 32H), 0.91–0.89 (m, 9H) ppm. ^13^C{1H} NMR (100 MHz, CD_3_OD): δ 74.2 (C-4), 71.0 (C-3), 66.9 (C-5), 66.4 (C-2), 63.0 (*C*HN), 51.6 (C-6), 33.9–23.7 (19C), 14.4 (3C) ppm. MS (ESI) *m*/*z* (%): 456.35 (100) [M + H]^+^. Anal. Calcd for C_28_H_58_ClNO_3_: C, 68.32; H, 11.88; N, 2.85. Found: C, 68.30; H, 11.90; N, 2.93.

### 4.2 Biology

#### 4.2.1 Preliminary Biological Screening toward Commercial Glycosidases

The percentage (%) of inhibition for compounds **14** and **15** toward the corresponding glycosidase was determined by quadruplicate in the presence of 100 μM of the inhibitor on the well. Each enzymatic assay (final volume 0.12 mL) contains 0.01–0.5 units/mL of the enzyme (with previous calibration) and 4.2 mM aqueous solution of the appropriate *p*-nitrophenyl glycopyranoside (substrate) buffered to the optimal pH of the enzyme. Enzyme and inhibitor were pre-incubated for 5 min at rt, and the reaction started by addition of the substrate. After 20 min of incubation at 37 °C, the reaction was stopped by addition of 0.1 mL of sodium borate solution (pH 9.8). The *p*-nitrophenolate formed was measured by visible absorption spectroscopy at 405 nm (Asys Expert 96 spectrophotometer). Under these conditions, the *p*-nitrophenolate released led to optical densities linear with both reaction time and concentration of the enzyme.

#### 4.2.2 Enzymatic Assays with Human GCase

##### 4.2.2.1 Biochemical Characterization with Human GCase

The compounds **14**, **15**, and **33·HCl** were screened toward GCase in leukocytes isolated from healthy donors (controls). Isolated leukocytes were disrupted by sonication, and a micro BCA protein assay kit (Sigma-Aldrich) was used to determine the total protein amount for the enzymatic assay, according to the manufacturer instructions. Enzyme activity was measured in a flat-bottomed 96-well plate. Compound solution (3 μL), 4.29 μg/μL leukocytes homogenate (7 μL), and substrate 4-methylumbelliferyl-β-d-glucoside (3.33 mM, 20 μL, Sigma-Aldrich) in citrate/phosphate buffer (0.1:0.2, M/M, pH 5.8) containing sodium taurocholate (0.3%) and Triton X-100 (0.15%) at 37 °C were incubated for 1 h. The reaction was stopped by addition of sodium carbonate (200 μL; 0.5 M, pH 10.7) containing Triton X-100 (0.0025%), and the fluorescence of 4-methylumbelliferone released by β-glucosidase activity was measured in SpectraMax M2 microplate reader (λ_ex_ = 365 nm, λ_em_ = 435 nm; Molecular Devices). Percentage GCase inhibition is given with respect to the control (without compound). Data are mean SD (*n* = 3).

##### 4.2.2.2 IC_50_ Determination

The IC_50_ values of inhibitors against GCase were determined by measuring the initial hydrolysis rate with 4-methylumbelliferyl-β-d-glucoside (3.33 mM). Data obtained were fitted by using the appropriate Equation (for more details, see the Supporting Information section).

#### 4.2.3 Preliminary Biological Screening toward Human Lysosomal Glycosidases

The effect of 1 mM concentration of **14**, **15**, and **33·HCl** was assayed toward six lysosomal glycosidases other than GCase, namely, α-mannosidase, β-mannosidase, α-galactosidase, β-galactosidase, α-fucosidase from leukocytes isolated from healthy donors (controls) and α-glucosidase from lymphocytes isolated from healthy donors’ flesh blood (controls). Isolated leukocytes or lymphocytes were disrupted by sonication, and a micro BCA protein assay kit (Sigma-Aldrich) was used to determine the total protein amount for the enzymatic assay, according to the manufacturer’s instructions (for more details, see the Supporting Information section).

#### 4.2.4 Pharmacological Chaperoning Activity

Fibroblasts with the N370S/RecNcil (or L444P/L444P) mutation from Gaucher disease patients were obtained from the “Cell line and DNA Biobank from patients affected by Genetic Diseases” (Gaslini Hospital, Genova, Italy). Fibroblast cells (15.0 × 10^4^) were seeded in T25 flasks with DMEM supplemented with fetal bovine serum (10%), penicillin/streptomycin (1%), and glutamine (1%) and incubated at 37 °C with 5% CO_2_ for 24 h. The medium was removed, and fresh medium containing the compounds (**14**, **15** and **33·HCl**) was added to the cells and incubated for 4 days. The medium was removed, and the cells were washed with PBS and detached with trypsin to obtain cell pellets, which were washed four times with PBS, frozen, and lysed by sonication in water. Enzyme activity was measured as reported above. Reported data are mean S.D. (*n* = 2).

#### 4.2.5 Cytotoxicity Test

The MTT test was carried out using the human fibroblasts wild type at different concentrations of compound **33·HCl**. Fibroblasts were grown in the presence of Dulbecco’s modified Eagle’s medium supplemented with 10% fetal bovine serum (FBS), 1% glutamine, and 1% penicillin–streptomycin, at 37 °C in controlled atmosphere with 5% CO_2_. For the experiments, cells were seeded at a density of 20,000 cells per well in 24-well plates and grown for 24 h (or 48 h) before adding the compound. The compound was dissolved in water; then, aliquots of these were diluted in the growth medium. To preserve sterility of solutions, these were filtered with 0.22 μm filters before adding to the dishes containing fibroblasts. Then, cells were incubated at 37 °C in 5% CO_2_ for 24 h (or 48 h). After this time, the media were replaced with medium containing 0.5 mg/mL of 3-(4,5-dimethylthiazol-2-yl)-2,5-diphenyltetrazolium bromide (MTT); the cells were incubated for an additional 1 h at 37 °C in 5% CO_2_. Finally, the number of viable cells was quantified by the estimation of their dehydrogenase activity, which reduces MTT to water-insoluble formazan. Growth medium was removed and substituted with 300 μL of DMSO to dissolve the formazan produced. The quantitation was carried out measuring the absorbance of samples at 570 nm with the iMark microplate absorbance reader (BIO RAD) in a 96-well format.

## Supporting Information Available

Copies of ^1^H NMR and ^13^C NMR spectra of all new compounds, 1D NOESY spectra for compounds **25** and **26**; biological screening toward commercial glycosidases; percentage of GCase inhibition of compounds **14**, **15**, and **33·HCl** toward human GCase. IC_50_ for compounds **14**, **15**, and **33·HCl** toward GCase; biological screening toward human lysosomal glycosidases; chaperoning activity assays on fibroblasts with the N370S/RecNcil and L444P/L444P mutations from Gaucher disease patients; and computational methods (docking and MD simulations), docked poses, final snapshots of MD simulations and full data of MMGBSA and MMPBSA data (PDF)

## Dedication

Dedicated to Prof. Alberto Brandi on the occasion of his 70th birthday.
