# Supplementary material for: Piperidine Azasugars Bearing Lipophilic Chains: Stereoselective Synthesis and Biological Activity as Inhibitors of Glucocerebrosidase (GCase)
Source: J Org Chem. 2021 Sep 1;86(18):12745–61. doi: 10.1021/acs.joc.1c01308 (PMC8453635; doi:10.1021/acs.joc.1c01308)
Supplement: Supplementary file 1 — jo1c01308_si_001.pdf [file jo1c01308_si_001.pdf]

Piperidine Azasugars Bearing Lipophilic Chains: Stereoselective Synthesis and Biological Activity as Inhibitors of Glucocerebrosidase (GCase)

Francesca Clemente,[†] *Camilla Matassini,*[†] *Sara Giachetti,*[†] *Andrea Goti,*[†] *Amelia Morrone,*[#] *Macarena Martínez-Bailén,*[£,†] *Sara Orta,*[‡] *Pedro Merino*[‡*] *and Francesca Cardona*[†,*]

[†] Dipartimento di Chimica 'Ugo Schiff' (DICUS), University of Firenze, via della Lastruccia 3-13, 50019 Sesto Fiorentino (FI), Italy. E-mail: francesca.cardona@unifi.it

[#]Paediatric Neurology Unit and Laboratories, Neuroscience Department, Meyer Children's Hospital, and Department of Neurosciences, Pharmacology and Child Health, University of Florence, Viale Pieraccini n. 24, 50139 Firenze, Italy

[£]Departamento de Química Orgánica, Facultad de Química, Universidad de Sevilla, c/ Prof. García González 1, E-41012 Sevilla, Spain

‡ Unidad de Glicobiología, Instituto de Biocomputación y Física de Sistemas Complejos (BIFI), Universidad de Zaragoza, 50009 Zaragoza, Spain. E-mail: pmerino@unizar.es

Dedicated to Prof. Alberto Brandi on the occasion of his 70th birthday

SUPPORTING INFORMATION

Table of contents

^{1}H NMR spectrum of compound **10a** S03
^{1}H NMR and $^{13}C\{1H\}$ NMR spectra of compound **10b** S04-S05
^{1}H NMR and $^{13}C\{1H\}$ NMR spectra of compound **10c** S06-S07
^{1}H NMR and $^{13}C\{1H\}$ NMR spectra of compound **10d** S08-S09
^{1}H NMR and $^{13}C\{1H\}$ NMR spectra of compound **10e** S10-S11
^{1}H NMR, $^{13}C\{1H\}$ NMR and 1D NOESY spectra of compound **10f** S12-S14
^{1}H NMR and $^{13}C\{1H\}$ NMR spectra of compound **10g** S15-S16
^{1}H NMR and $^{13}C\{1H\}$ NMR spectra of compound **10h** S17-S18
^{1}H NMR spectrum of compound **16** S19
^{1}H NMR and $^{13}C\{1H\}$ NMR spectra of compound **17** S20-S21
^{1}H NMR and $^{13}C\{1H\}$ NMR spectra of compound **18** S22-S23
^{1}H NMR spectrum of compound **19** S24
^{1}H NMR spectra of compound **21** S25
^{1}H NMR spectra of compound **22** S26
^{1}H NMR and $^{13}C\{1H\}$ NMR spectra of compound **23** S27-S28
^{1}H NMR and $^{13}C\{1H\}$ NMR spectra of compound **24** S29-S30
^{1}H NMR and $^{13}C\{1H\}$ NMR and 1D NOESY spectra of compound **25** S31-S33
^{1}H NMR and $^{13}C\{1H\}$ NMR and 1D NOESY spectra of compound **26** S34-S36
^{1}H NMR and $^{13}C\{1H\}$ NMR spectra of compound **14** S37-S38
^{1}H NMR and $^{13}C\{1H\}$ NMR spectra of compound **15** S39-S40
^{1}H NMR and $^{13}C\{1H\}$ NMR spectra of compound **31** S41-S42
^{1}H NMR and $^{13}C\{1H\}$ NMR spectra of compound **32** S43-S44
^{1}H NMR and $^{13}C\{1H\}$ NMR spectra of compound **33·HCl** S45-S46
Biological screening towards commercial glycosidases of compounds **14** and **15** S47
Percentage of GCase inhibition of compounds **14, 15** and **33·HCl** towards human GCase S48
IC_{50} determination and graphs of compounds **14**, **15** and **33·HCl** S48-S50
Selectivity of compounds **14, 15** and **33·HCl** towards lysosomal enzymes S50-S51
Cytotoxicity test of compound **33·HCl** S52
Chaperoning activity assays for compounds **14, 15** and **33·HCl** in human fibroblasts derived from GD patients bearing N370S/RecNcil mutations S53-S54
Chaperoning activity assays for compounds **14** and **15** in human fibroblasts derived from GD patients bearing L444P/L444P mutations S55
Molecular Docking S56-S59
Molecular Dynamics (MD) simulations S59-S62
References S63

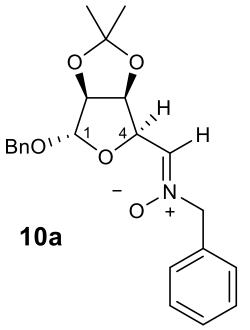

10a

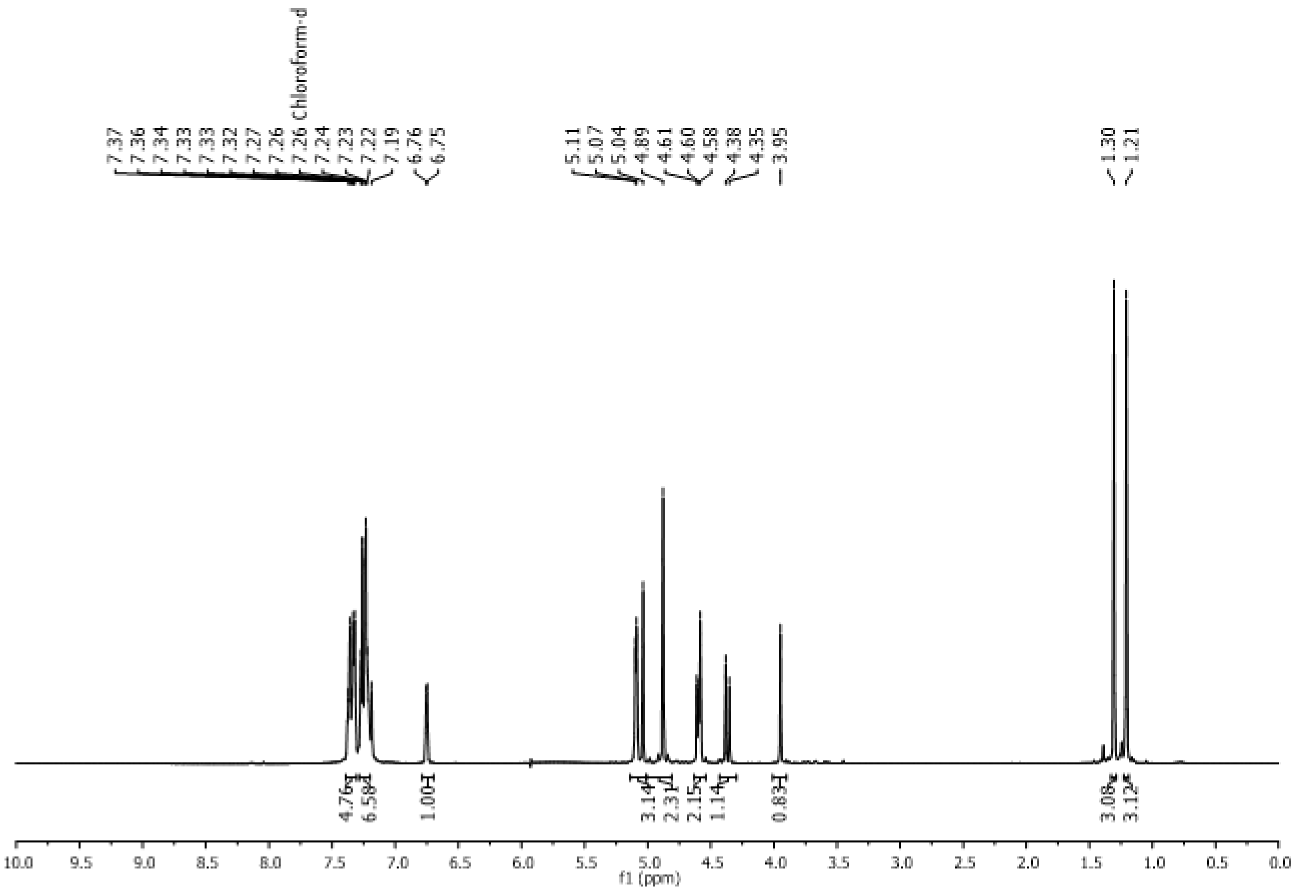

Figure S01. ^{1}H NMR spectrum of compound **10a** (400 MHz, $CDCl_3$).

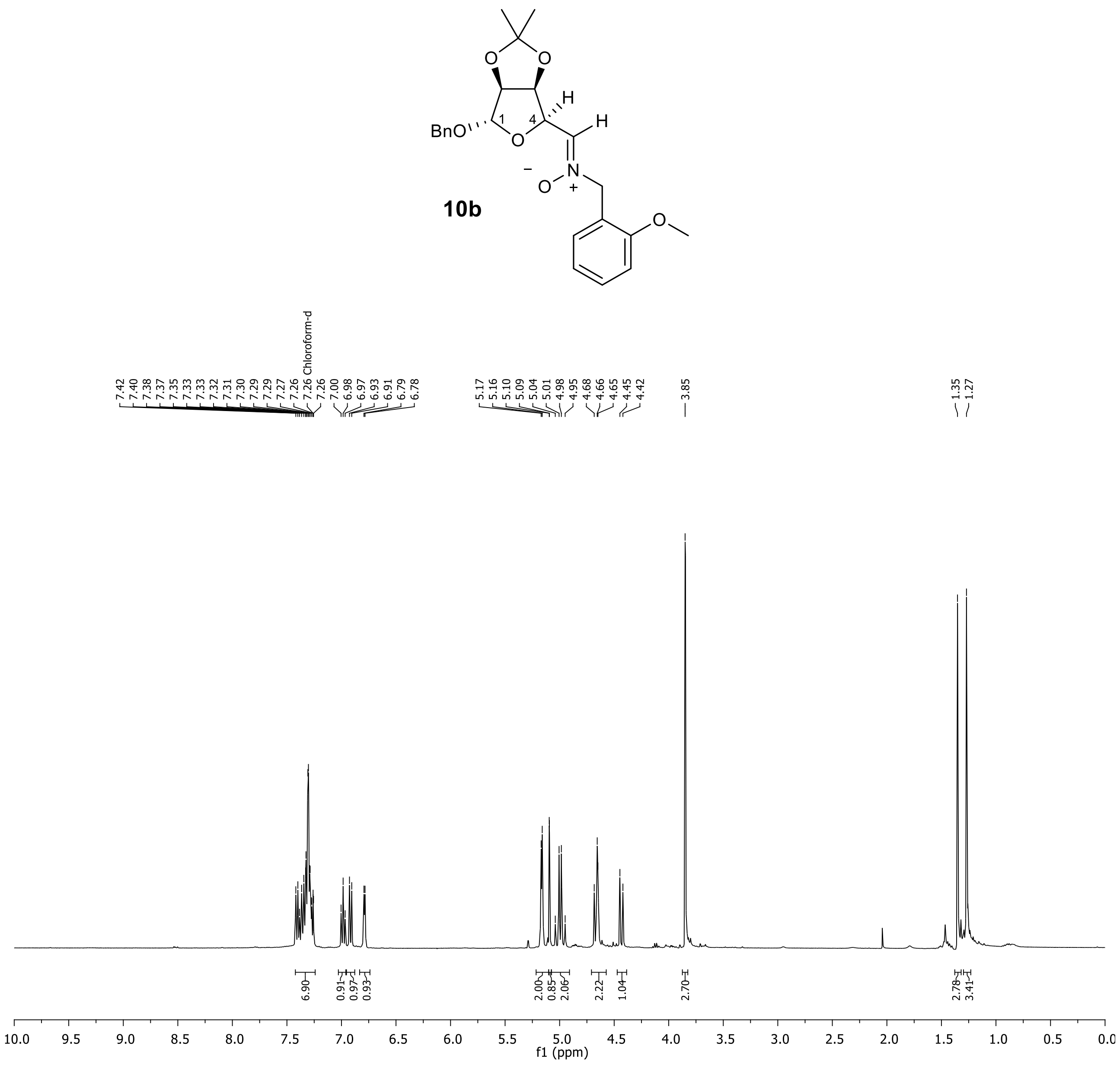

Figure S02. ^{1}H NMR spectrum of compound **10b** (400 MHz, $CDCl_3$).

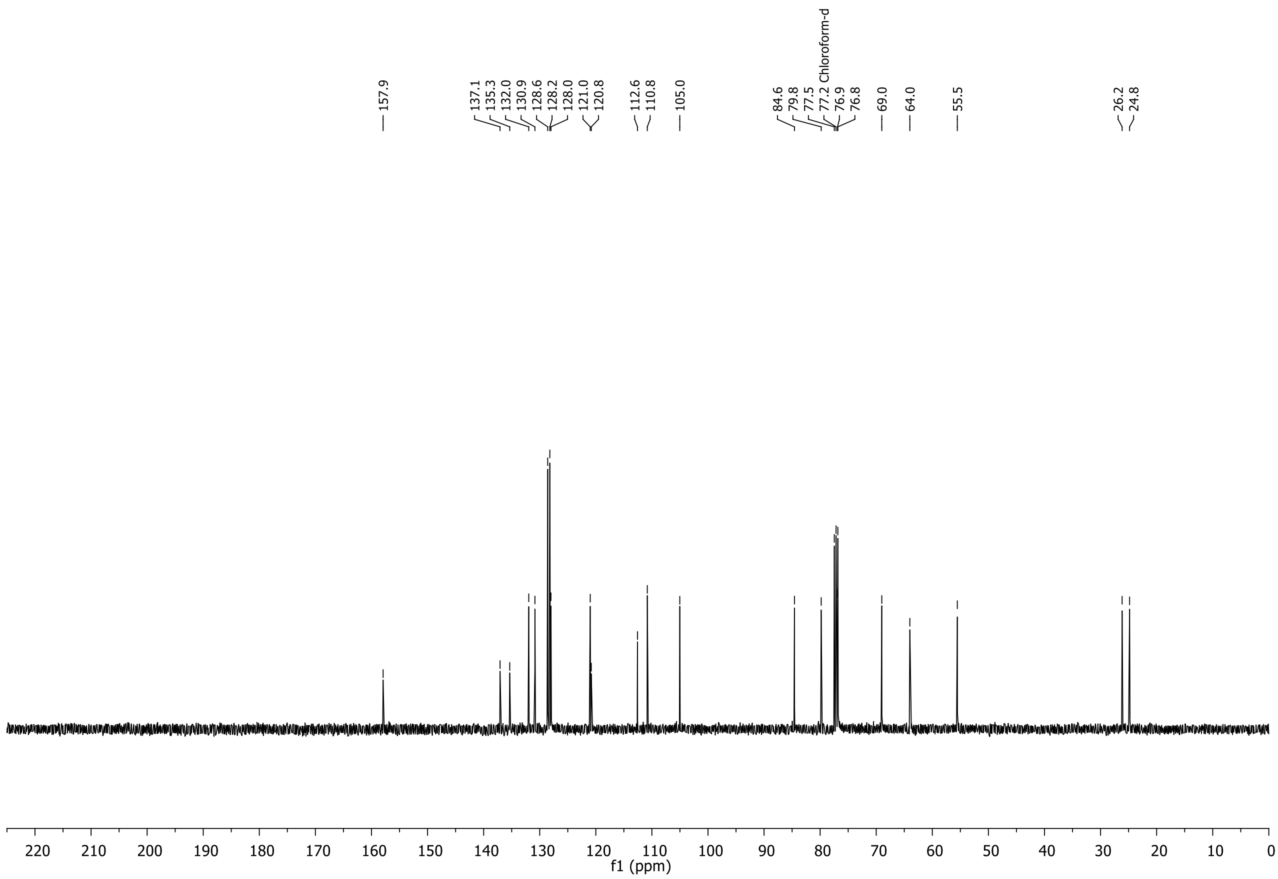

Figure S03. $^{13}C\{1H\}$ NMR spectrum of compound **10b** (100 MHz, $CDCl_3$).

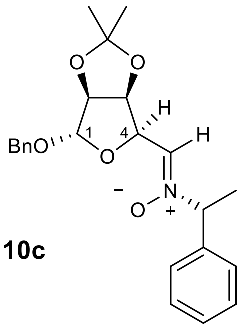

10c

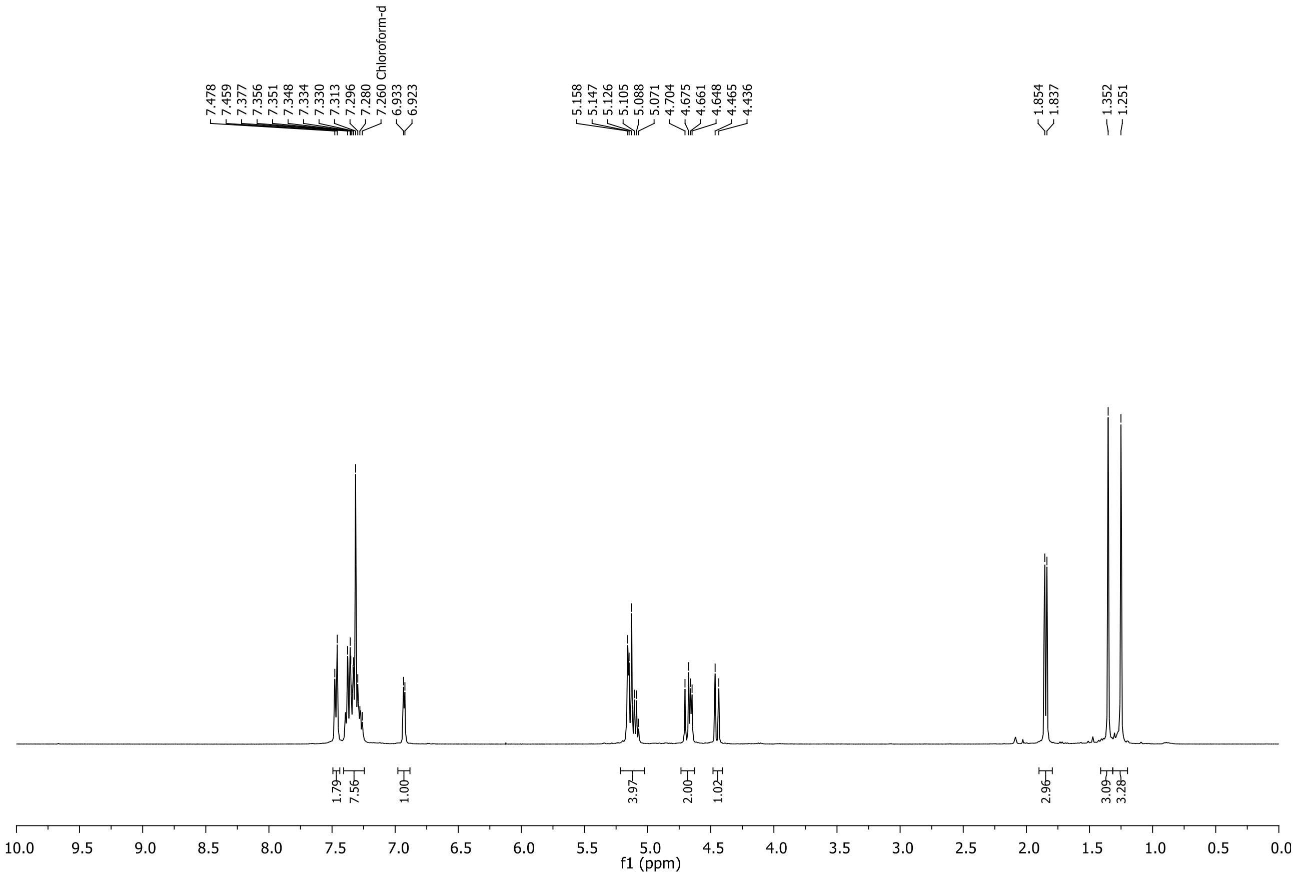

Figure S04. ^{1}H NMR spectrum of compound **10c** (400 MHz, $CDCl_3$).

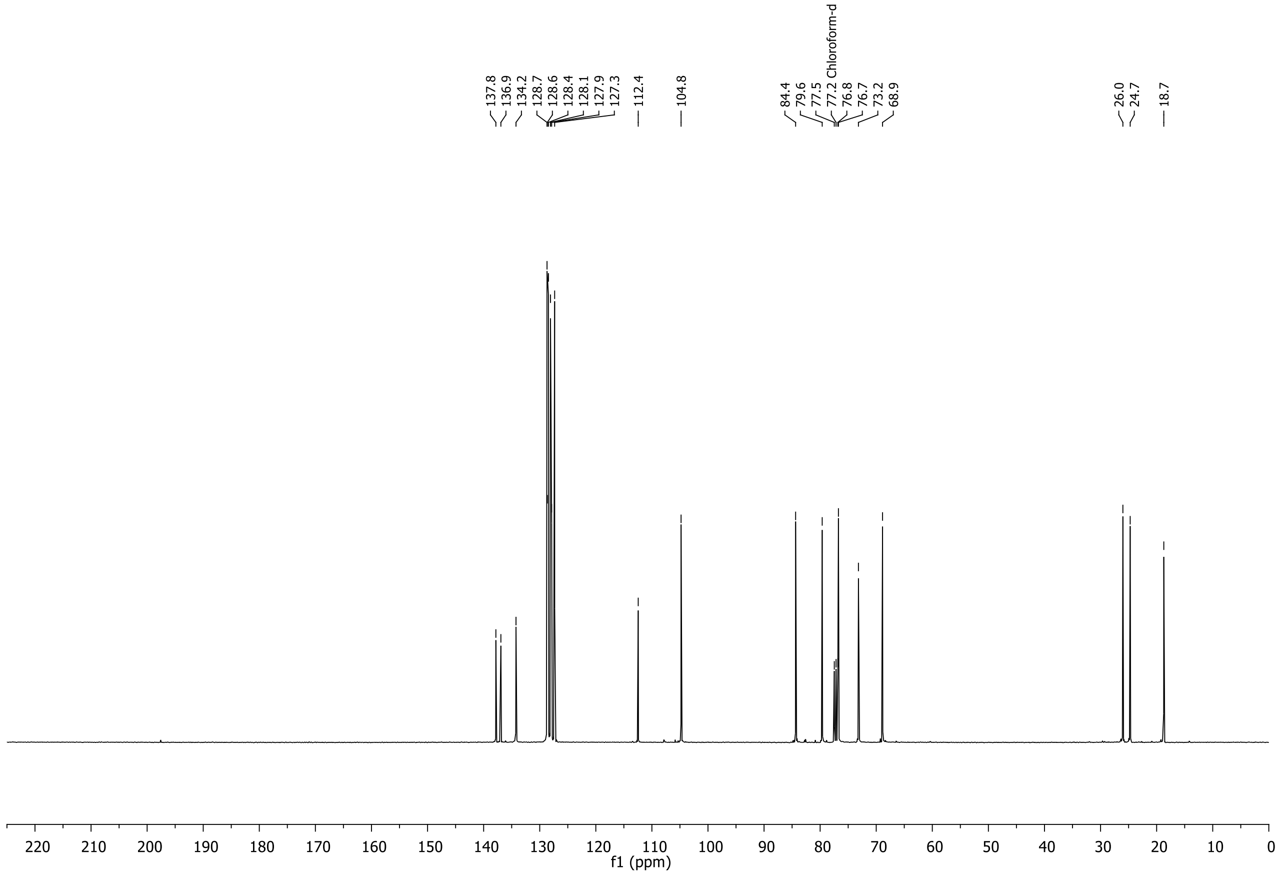

Figure S05. $^{13}C\{^1H\}$ NMR spectrum of compound **10c** (100 MHz, $CDCl_3$).

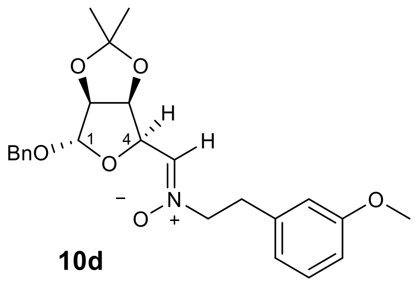

10d

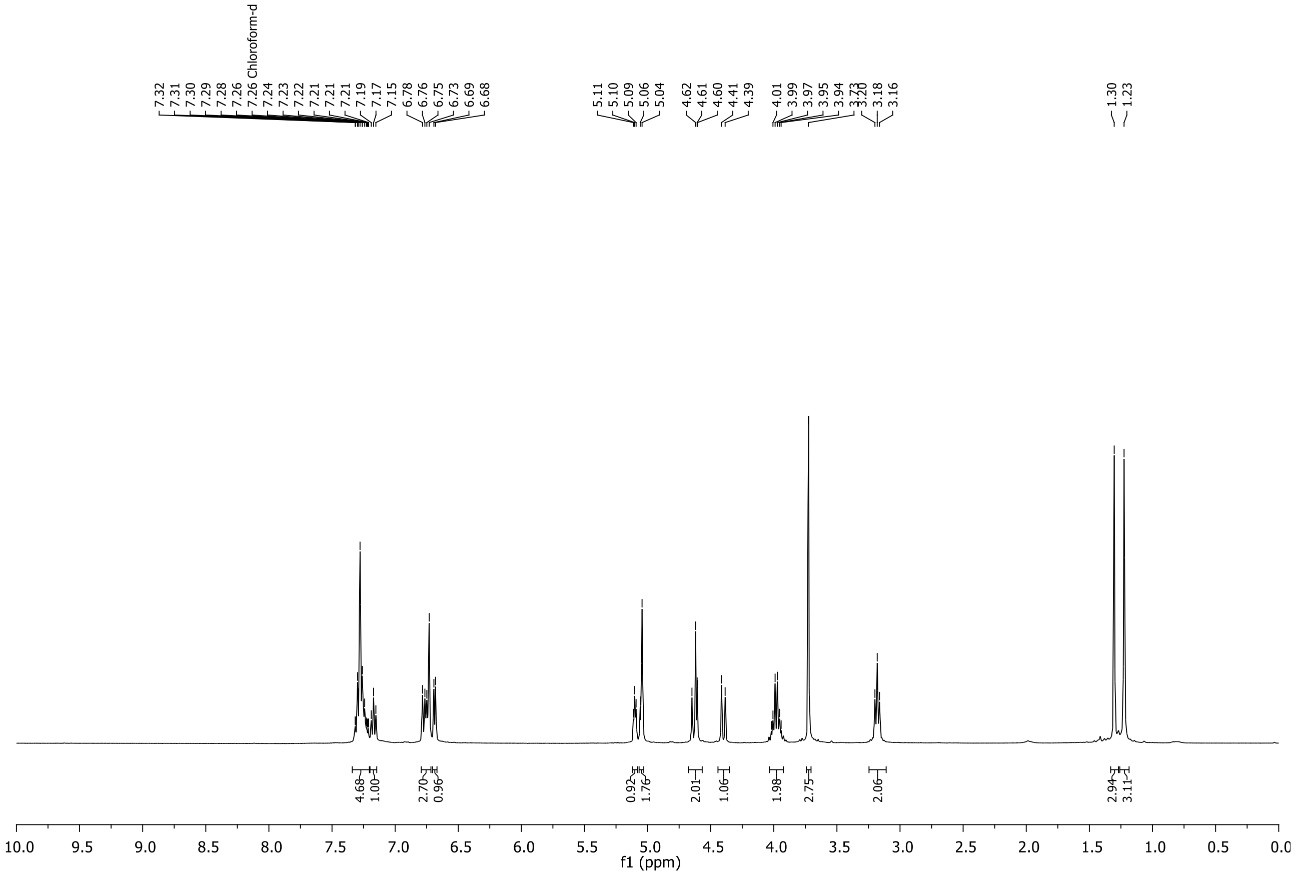

Figure S06. ^{1}H NMR spectrum of compound **10d** (400 MHz, $CDCl_3$).

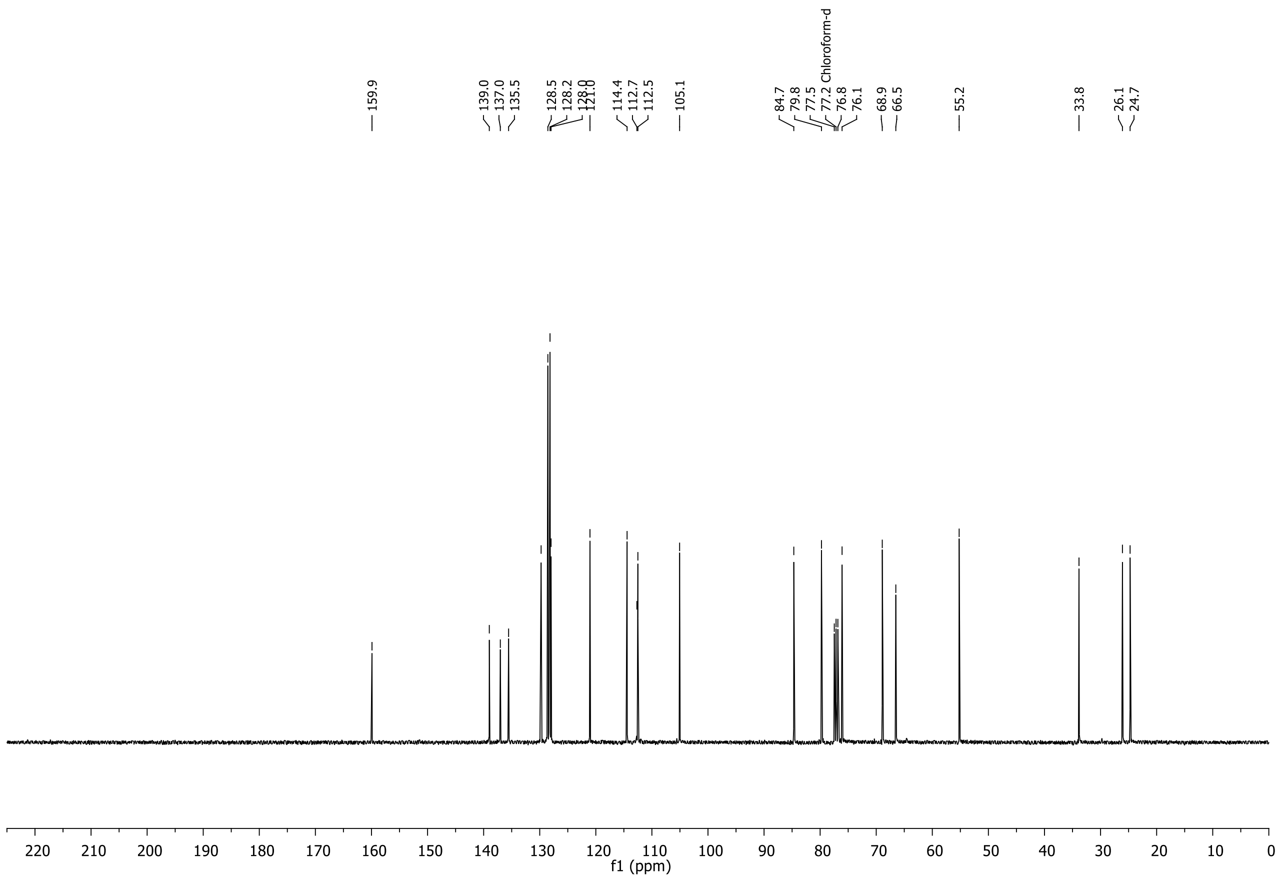

Figure S07. $^{13}C\{1H\}$ NMR spectrum of compound **10d** (100 MHz, $CDCl_3$).

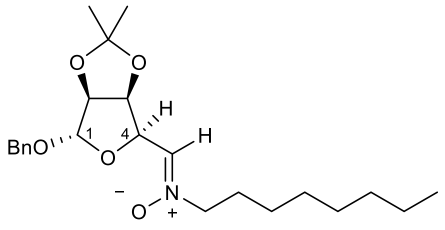

10e

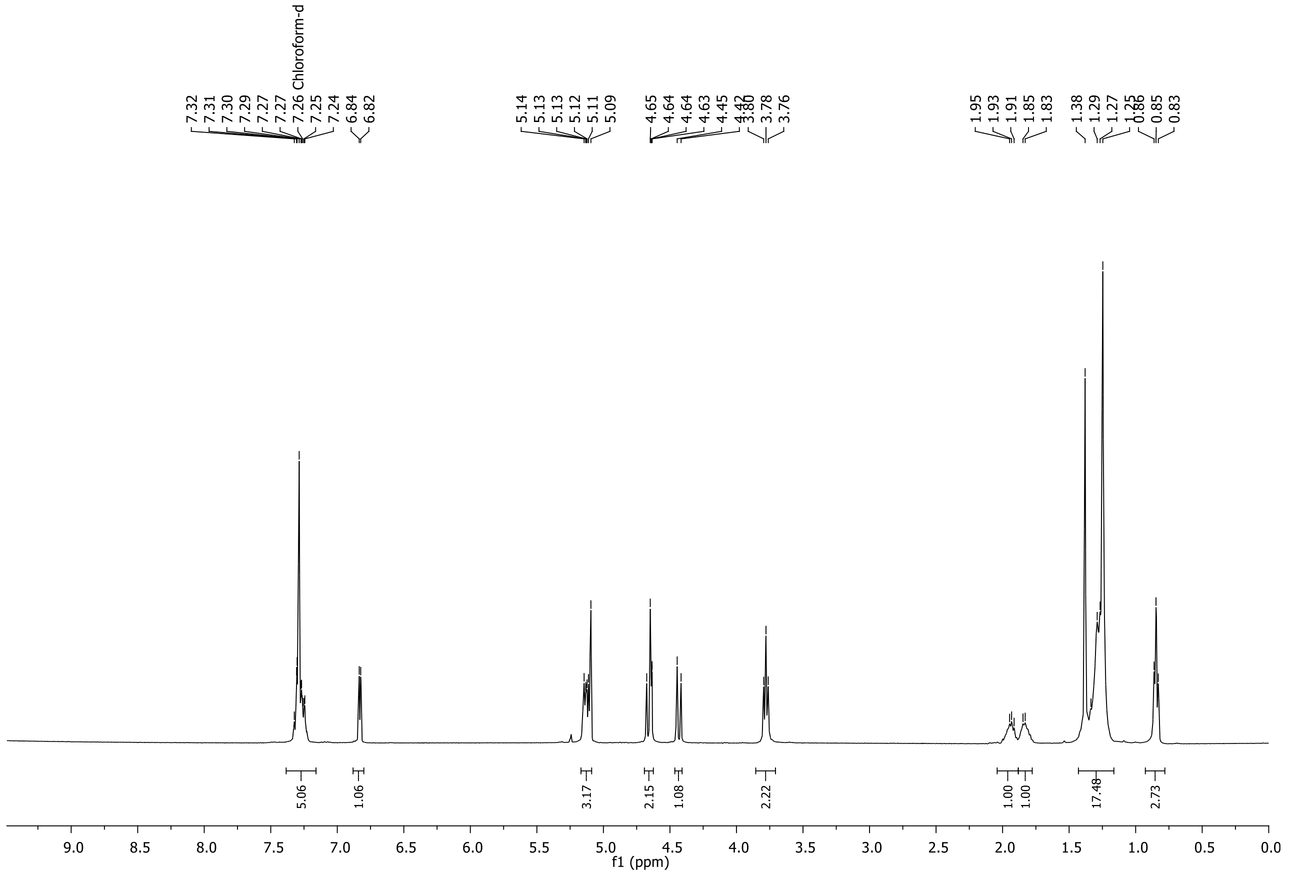

Figure S08. ^{1}H NMR spectrum of compound **10e** (400 MHz, $CDCl_3$).

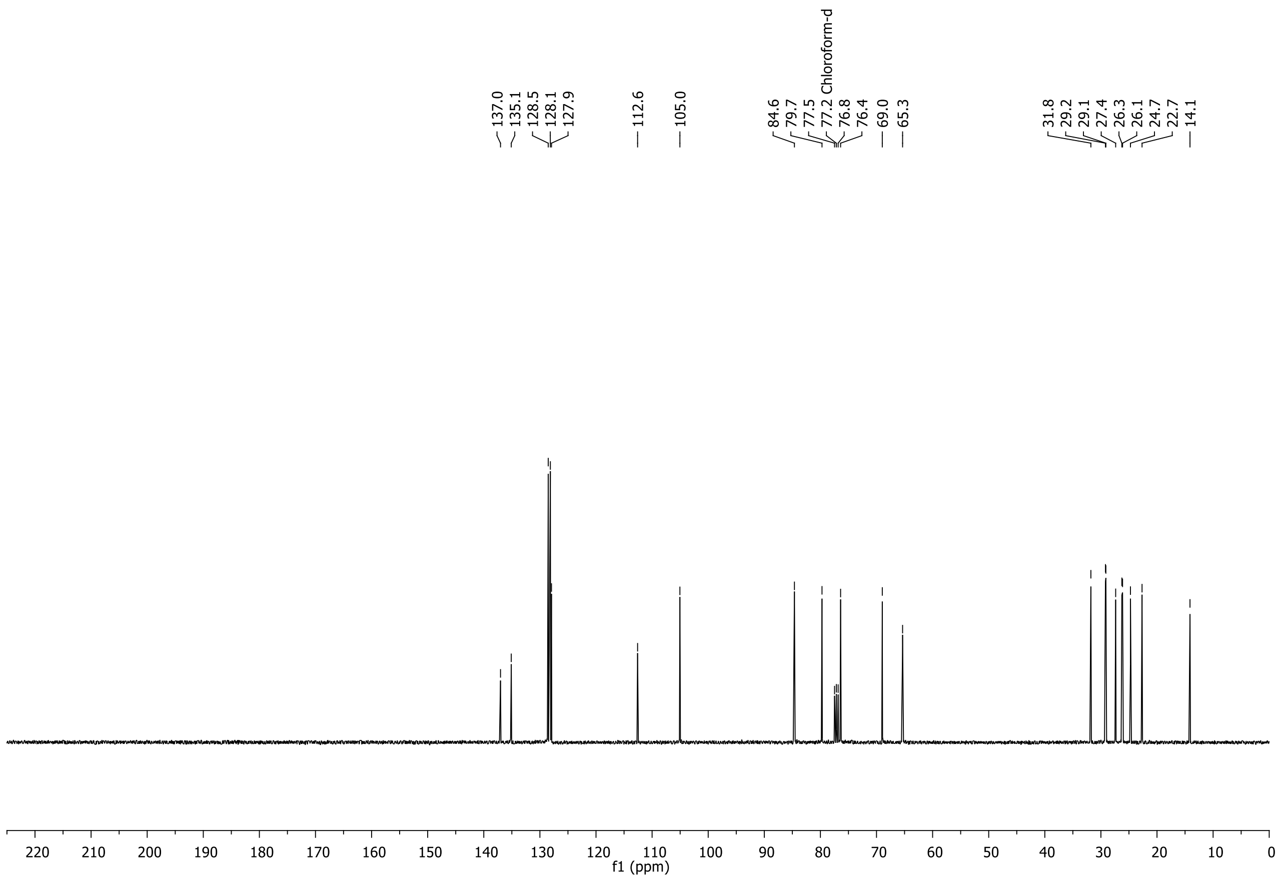

Figure S09. $^{13}C\{1H\}$ NMR spectrum of compound **10e** (100 MHz, $CDCl_3$).

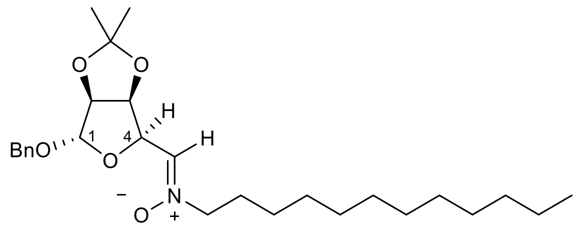

10f

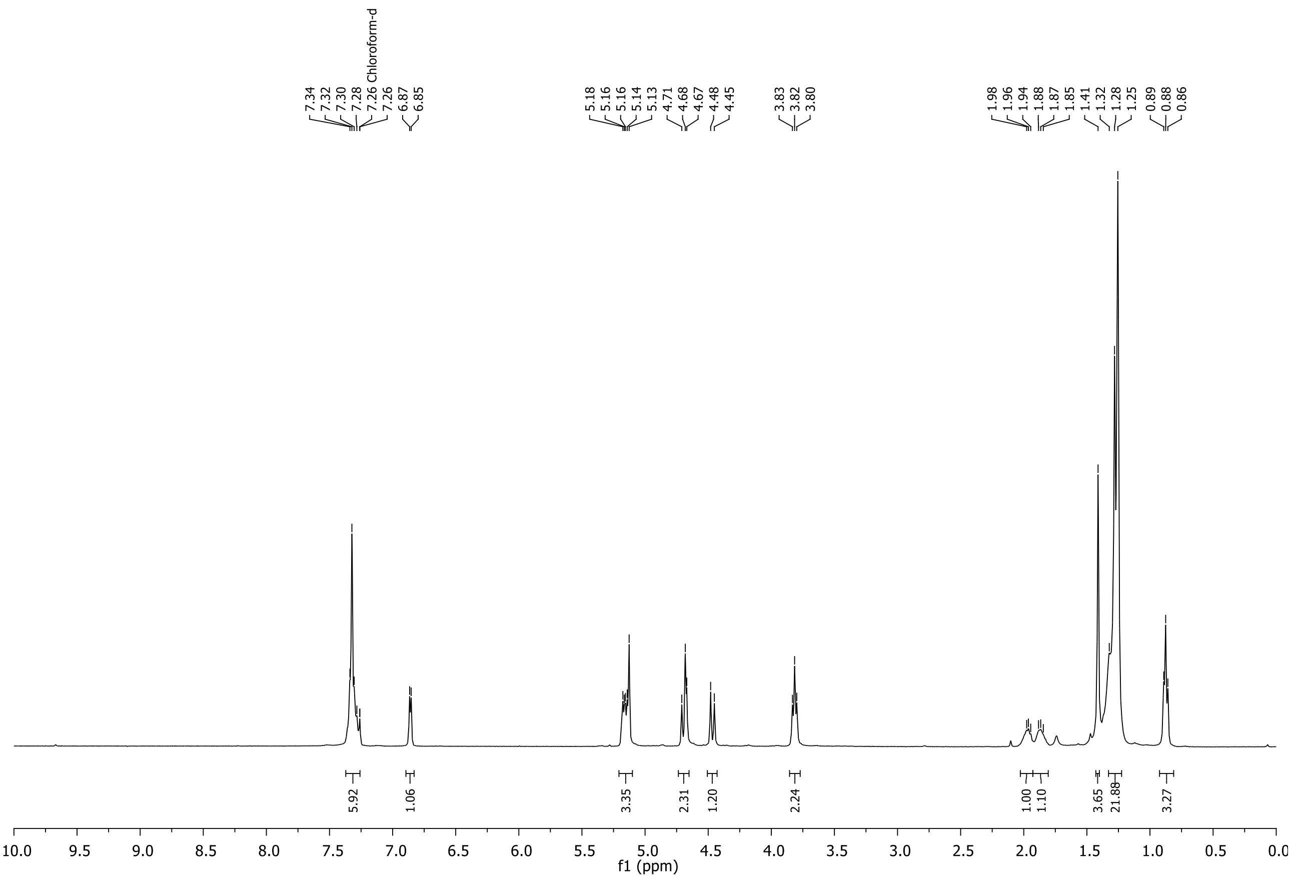

Figure S10. ^{1}H NMR spectrum of compound **10f** (100 MHz, $CDCl_3$).

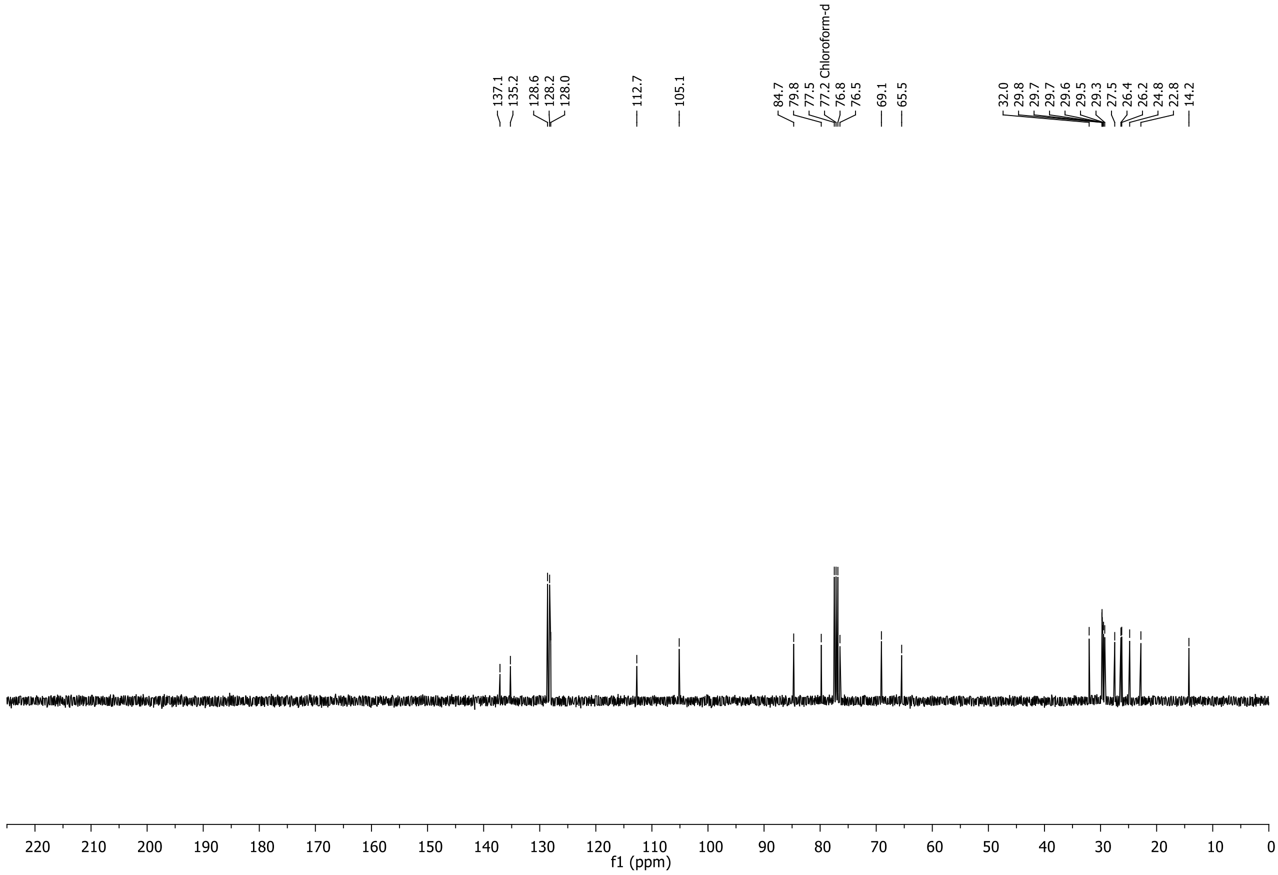

Figure S11. $^{13}C\{1H\}$ NMR spectrum of compound **10f** (100 MHz, $CDCl_3$).

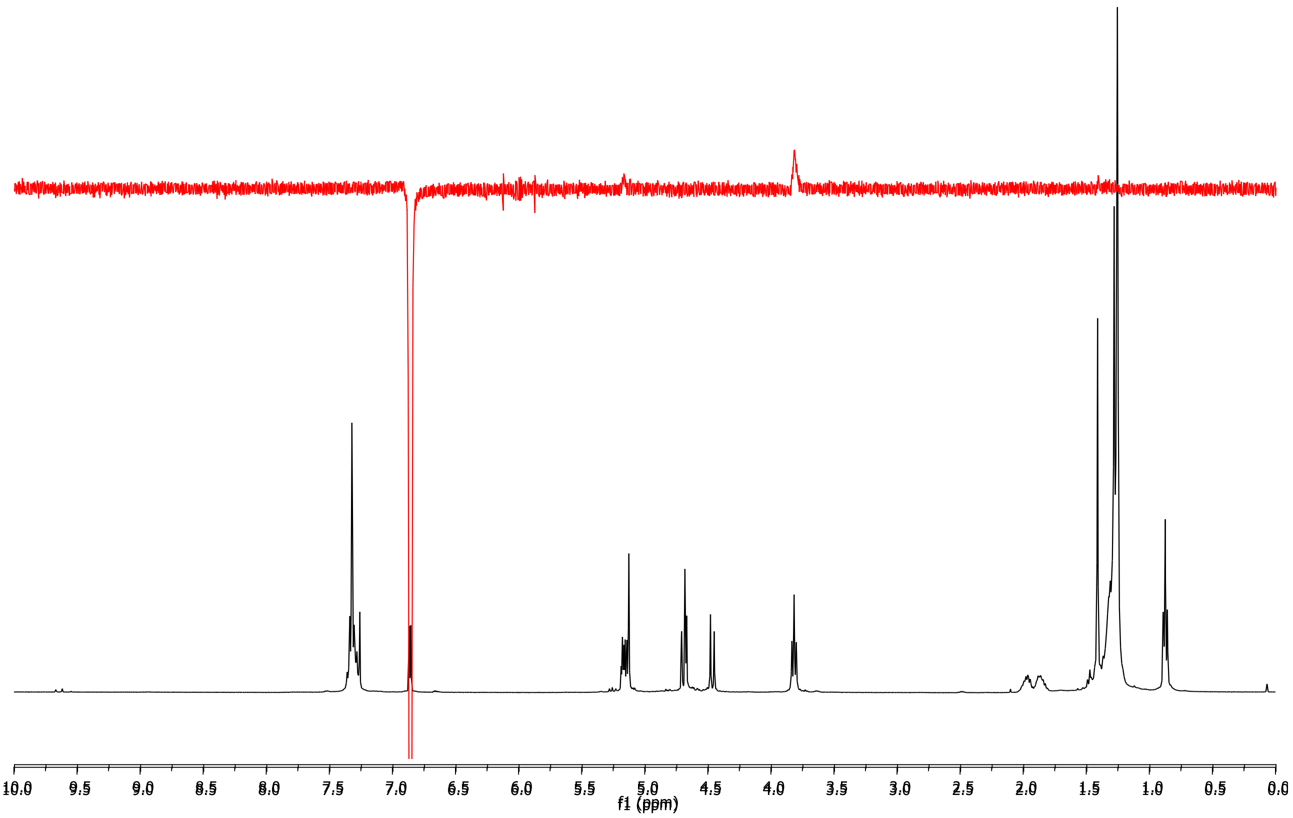

Figure S12. 1D NOESY spectra performed on compound **10f**. Irradiation of *H*C=N gave a NOE at CH_2N.

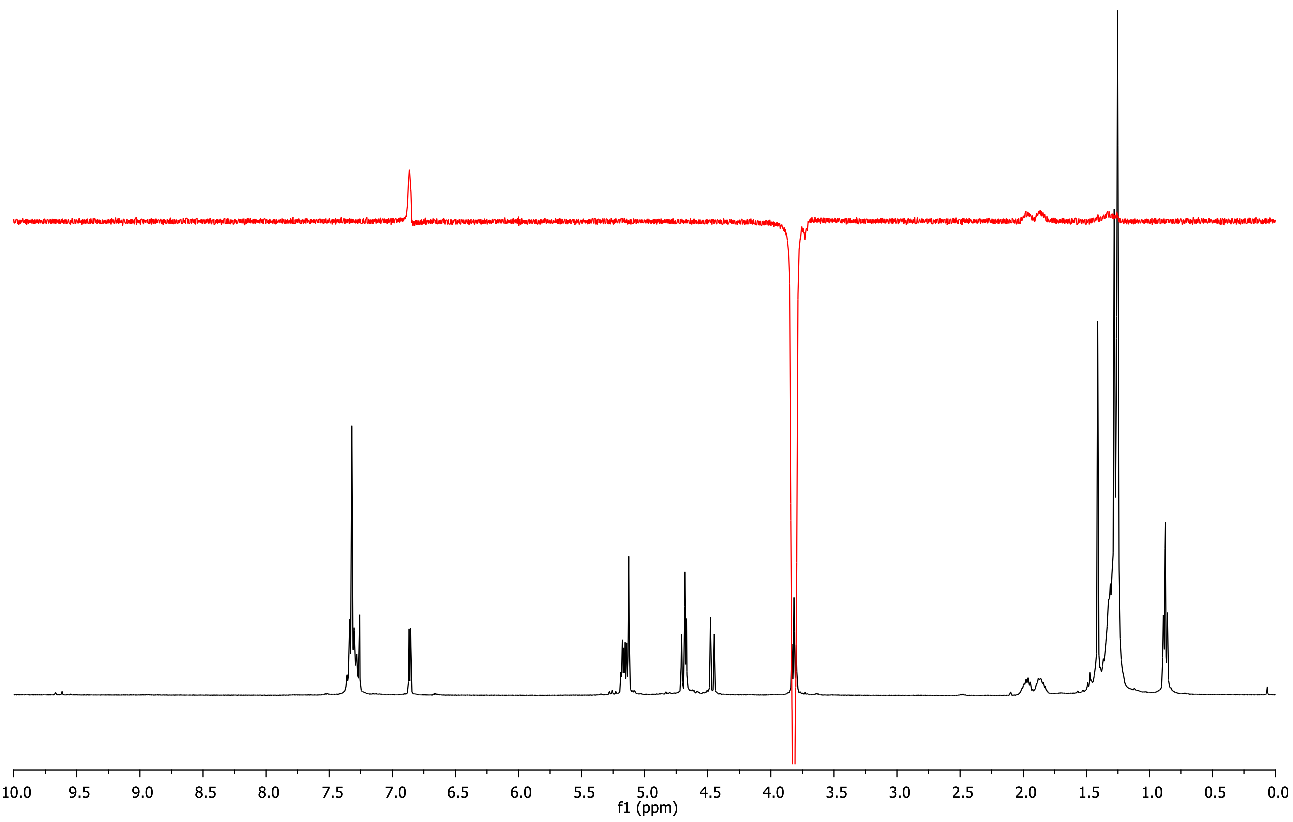

Figure S13. 1D NOESY spectra performed on compound **10f**. Irradiation of CH_2N gave a NOE at *H*C=N.

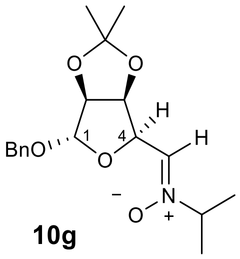

10g

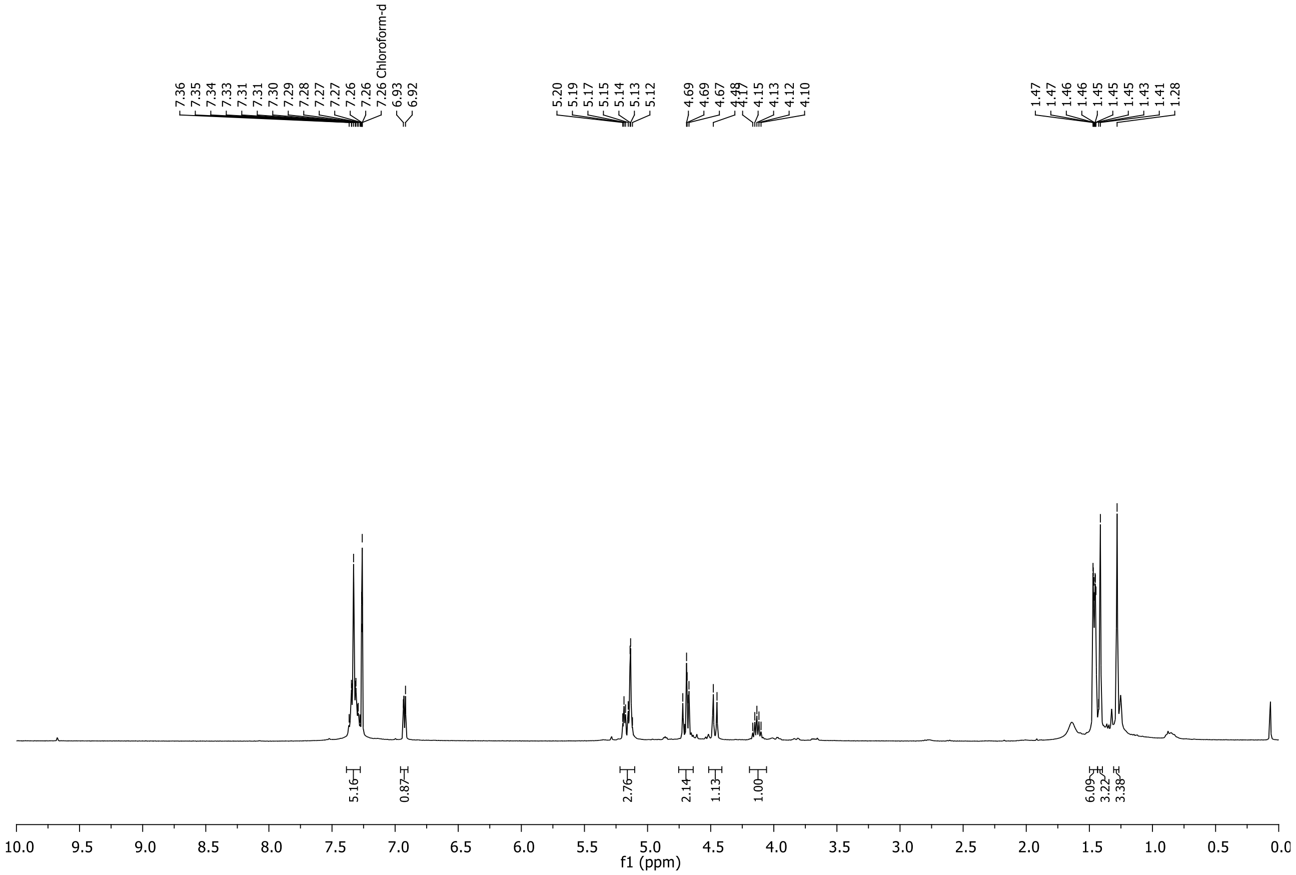

Figure S14. ^{1}H NMR spectrum of compound **10g** (400 MHz, $CDCl_3$).

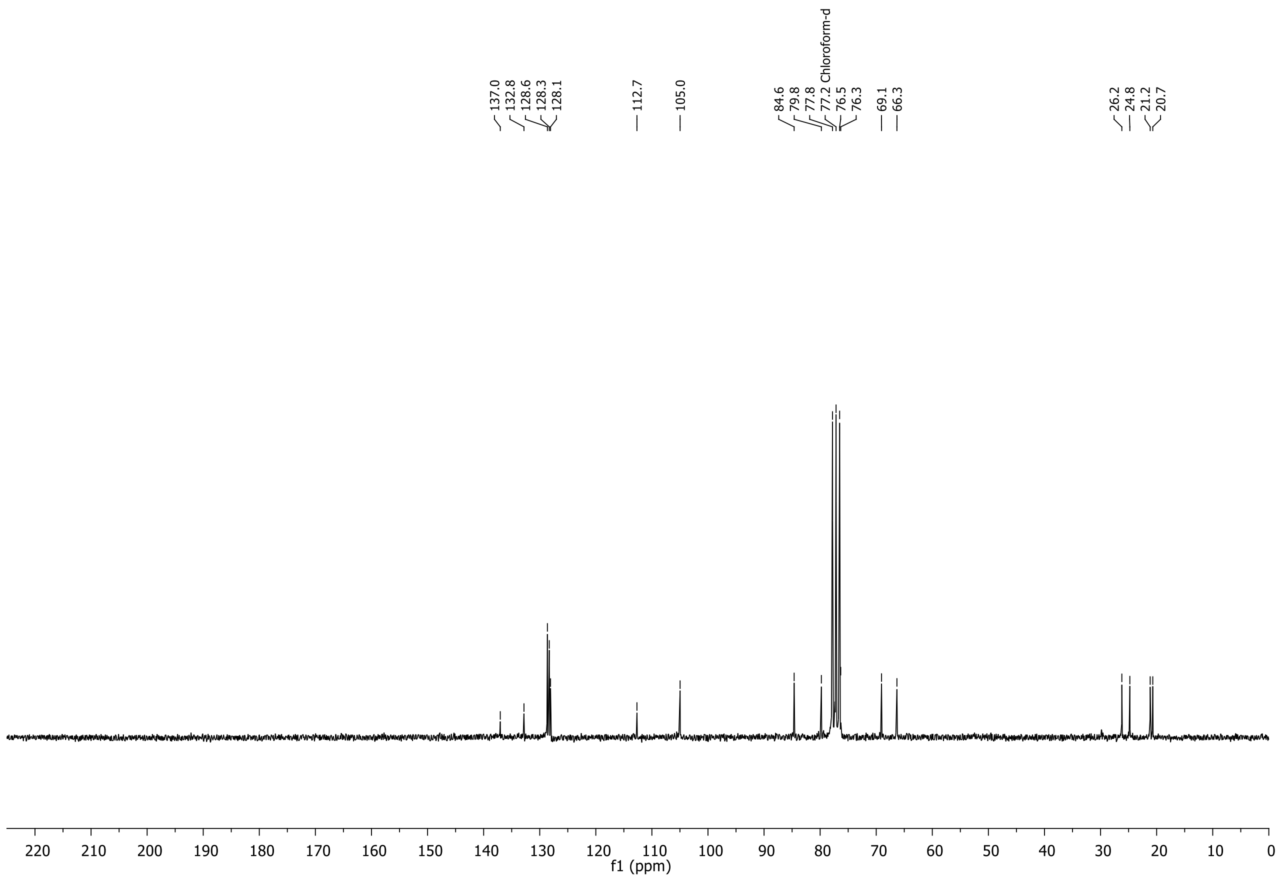

Figure S15. $^{13}C\{^1H\}$ NMR spectrum of compound **10g** (50 MHz, $CDCl_3$).

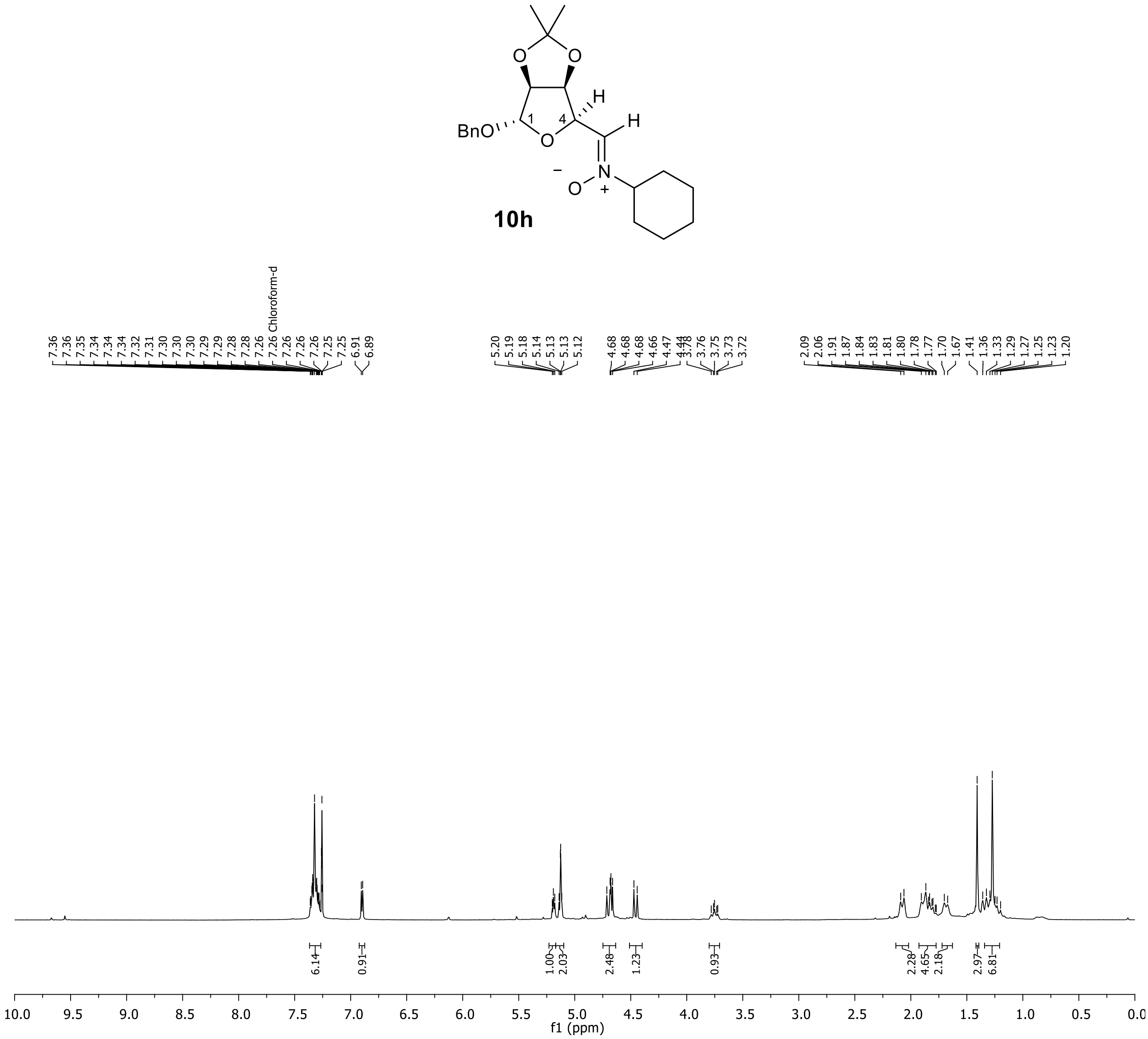

Figure S16. ^{1}H NMR spectrum of compound **10h** (400 MHz, $CDCl_3$).

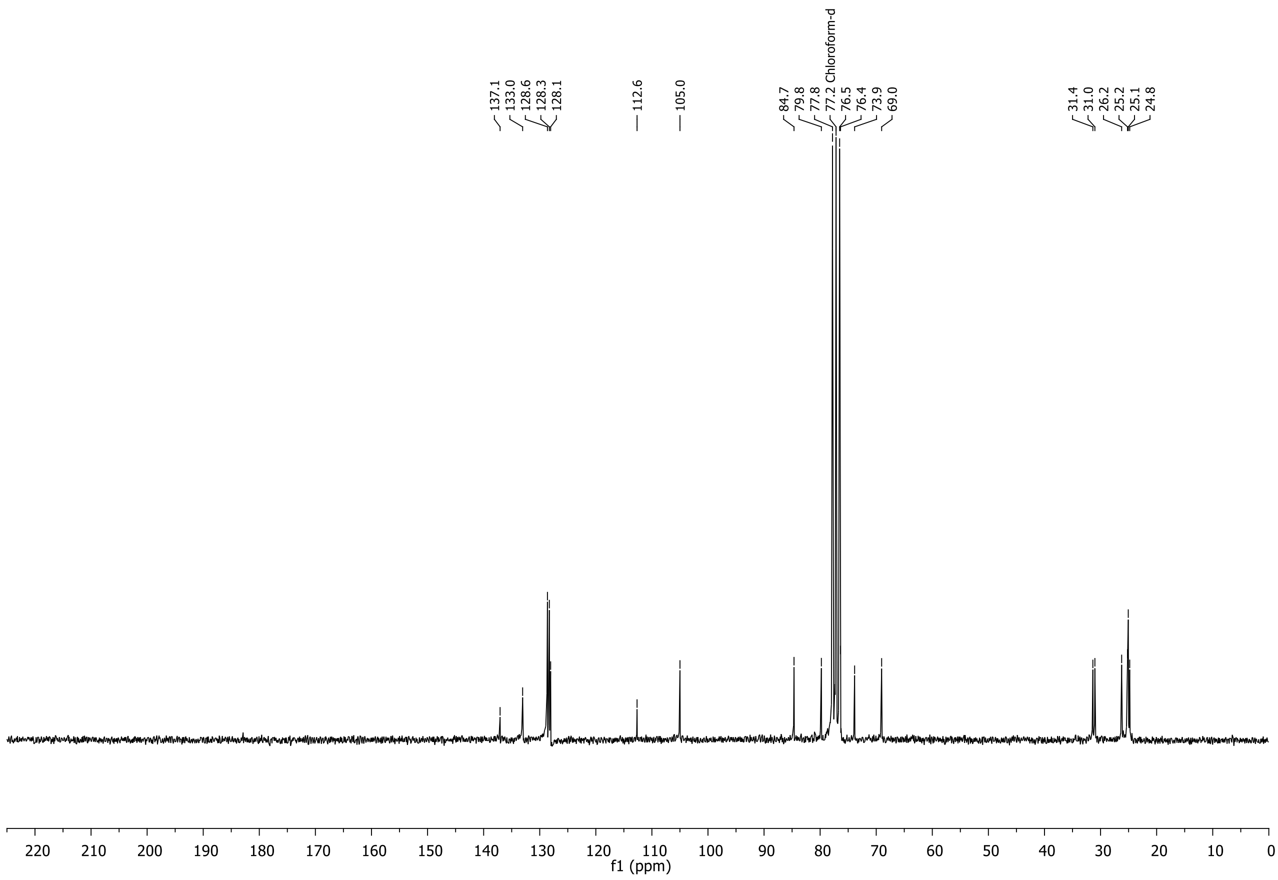

Figure S17. $^{13}C\{1H\}$ NMR spectrum of compound **10h** (50 MHz, $CDCl_3$).

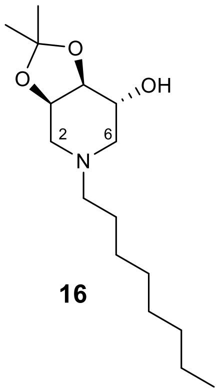

16

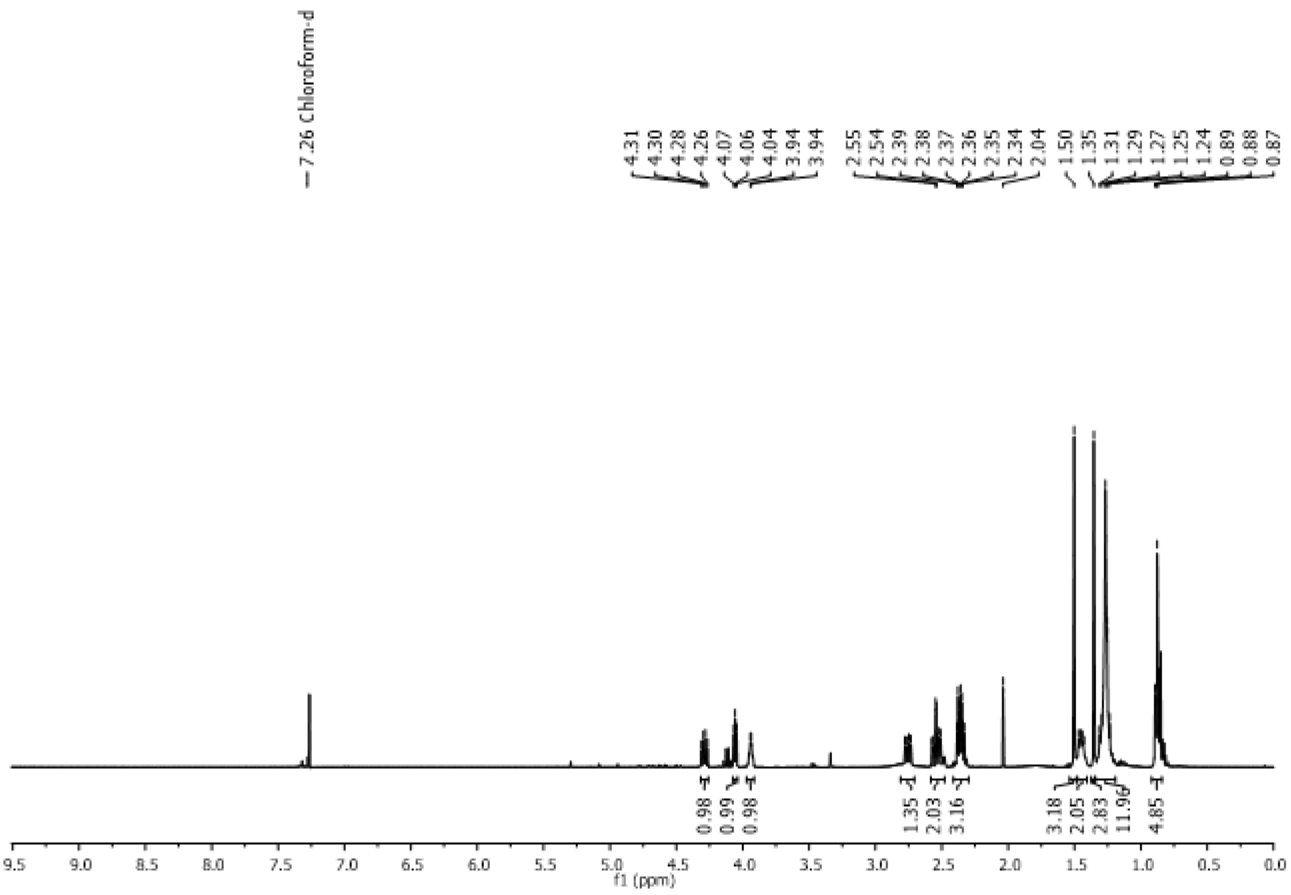

Figure S18. ^{1}H NMR spectrum of compound **16** (400 MHz, $CDCl_3$).

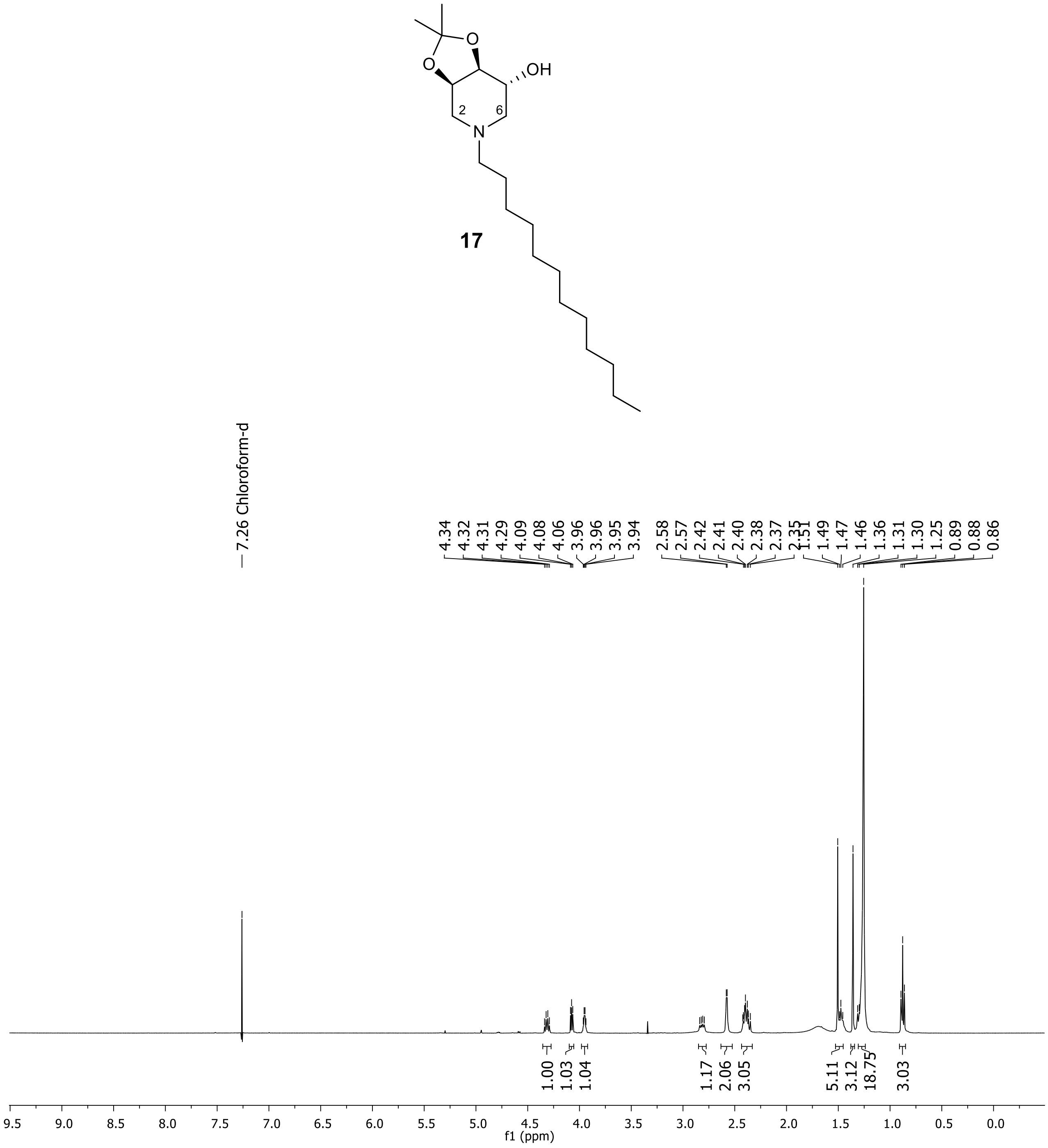

Figure S19. ^{1}H NMR spectrum of compound **17** (400 MHz, $CDCl_3$).

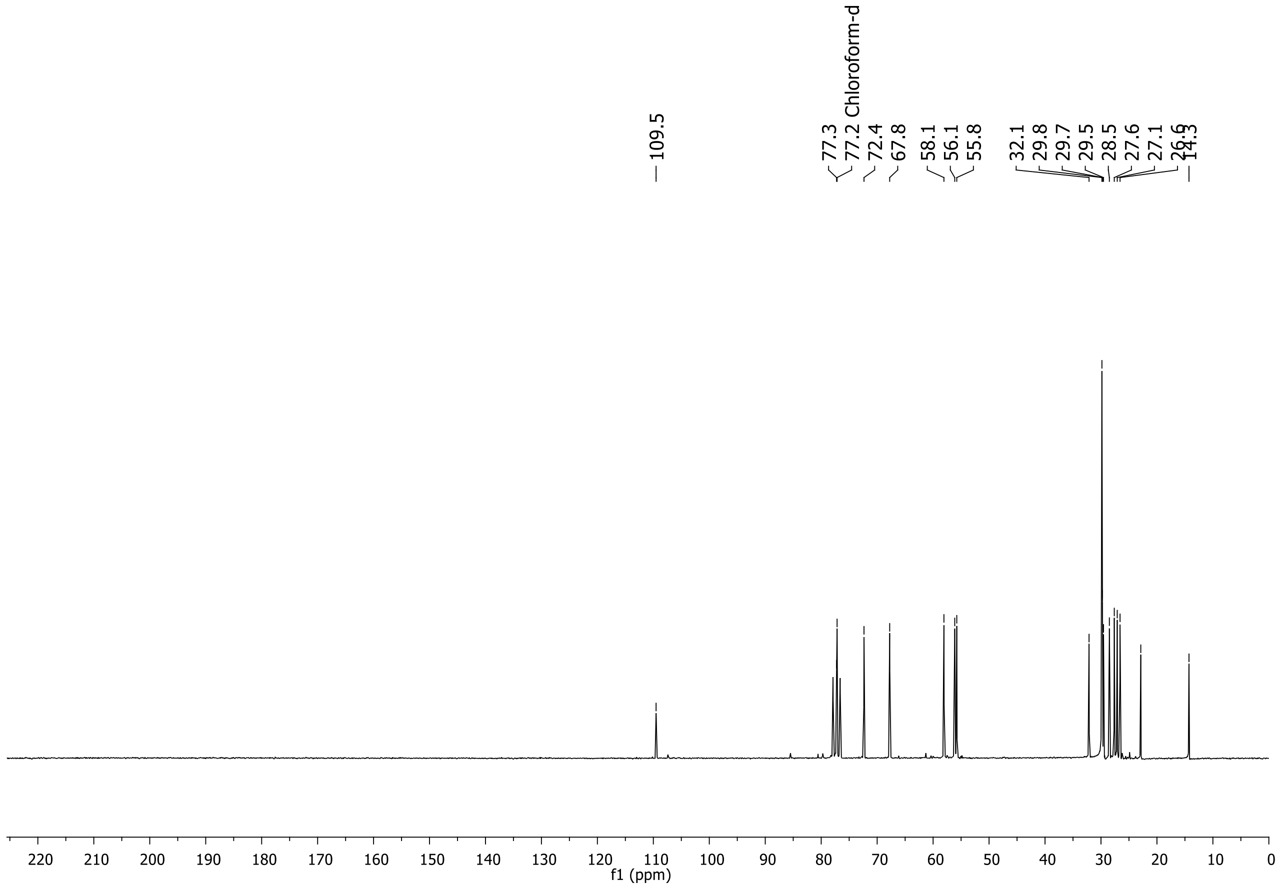

Figure S20. $^{13}C\{1H\}$ NMR spectrum of compound **17** (50 MHz, $CDCl_3$).

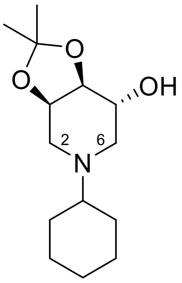

18

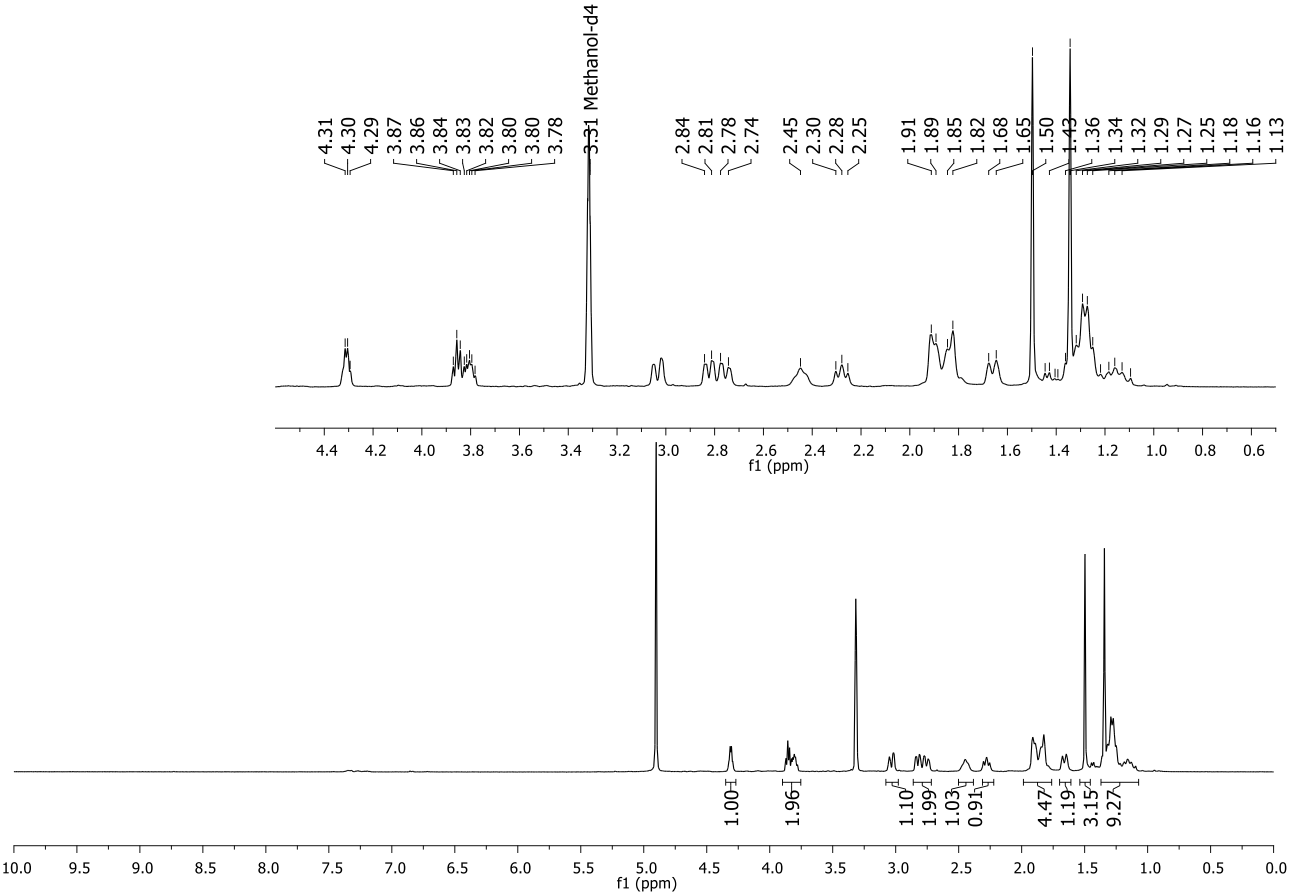

Figure S21. ^{1}H NMR spectrum of compound **18** (400 MHz, CD_3OD).

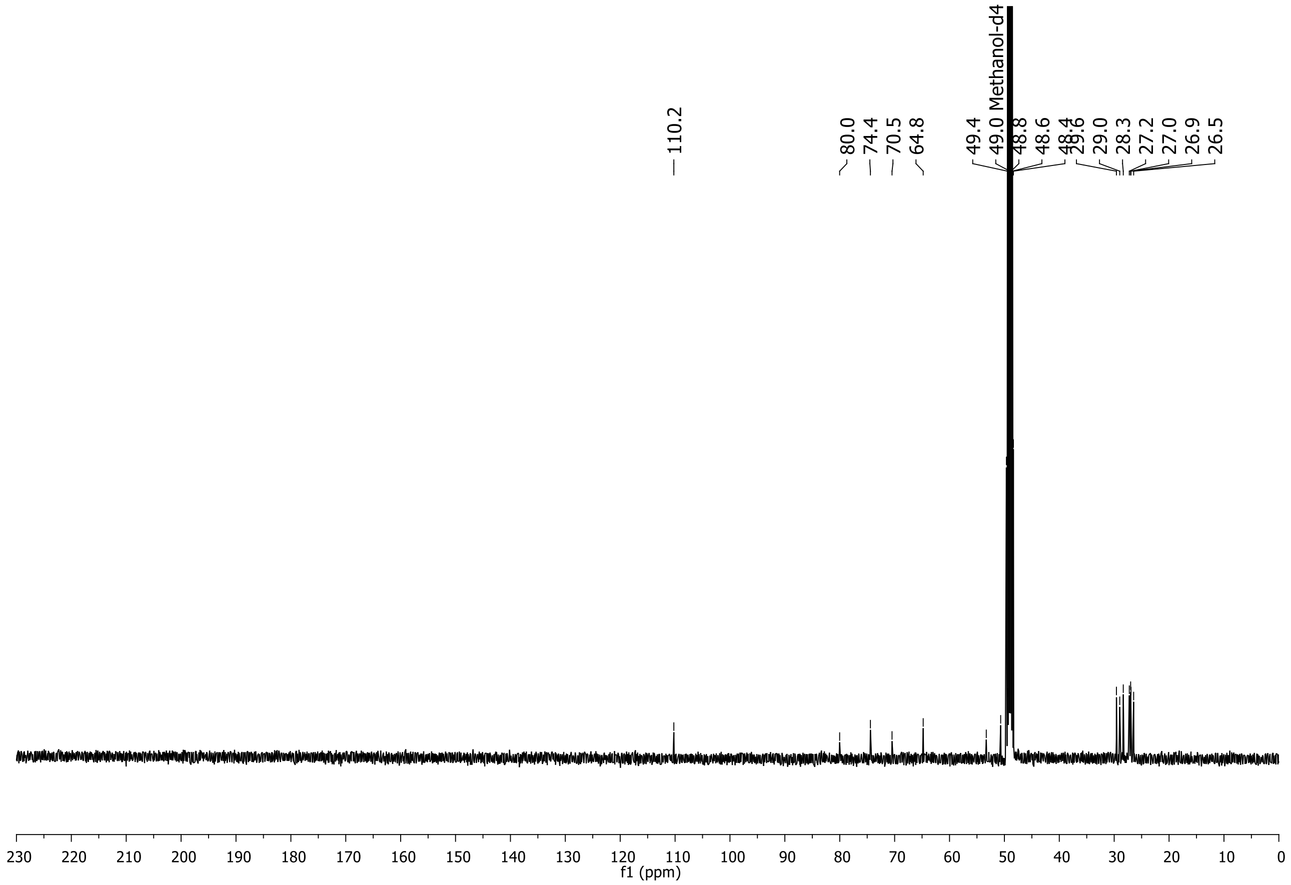

Figure S22. $^{13}C\{1H\}$ NMR spectrum of compound **18** (100 MHz, CD_3OD).

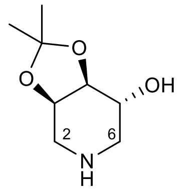

19

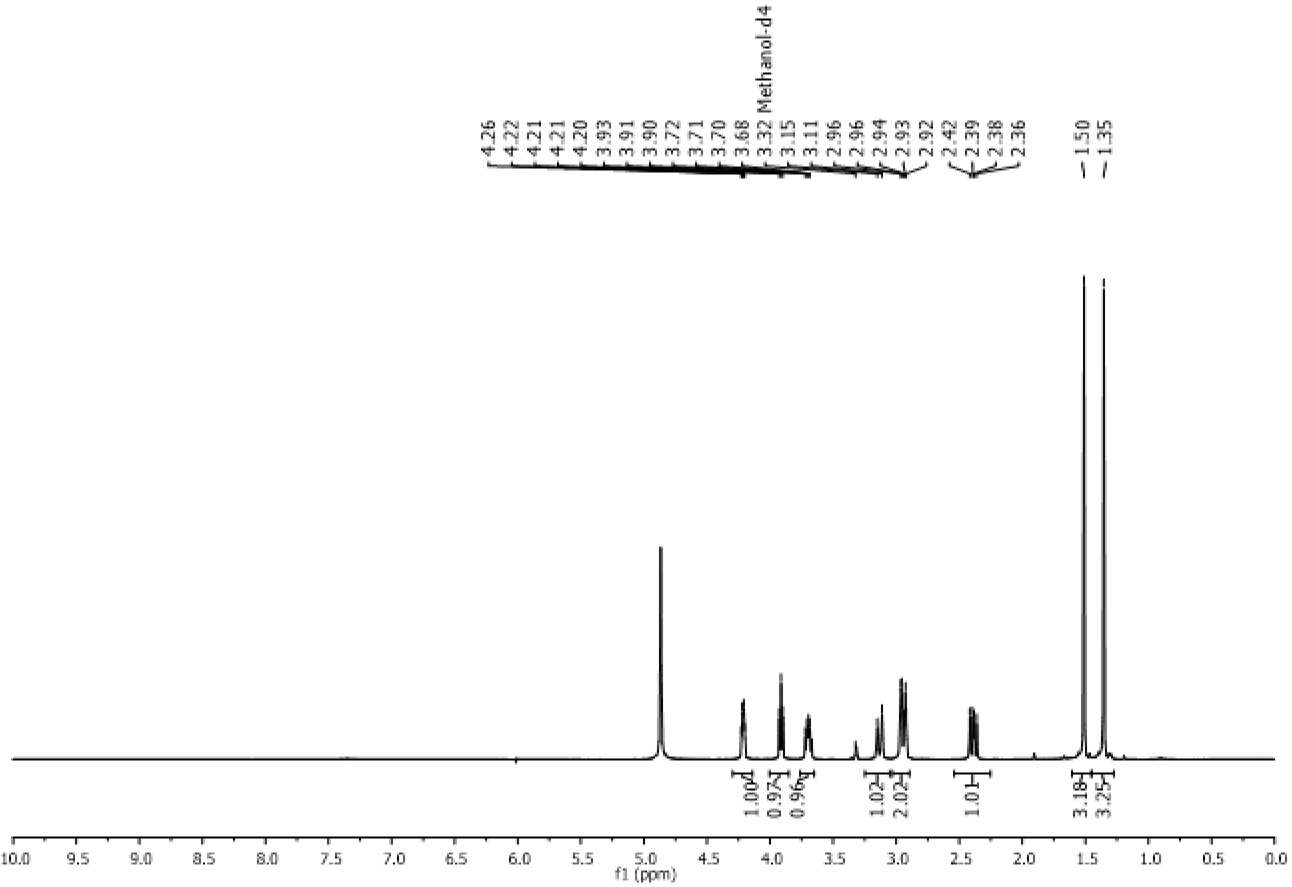

Figure S23. ^{1}H NMR spectrum of compound **19** (400 MHz, CD_3OD).

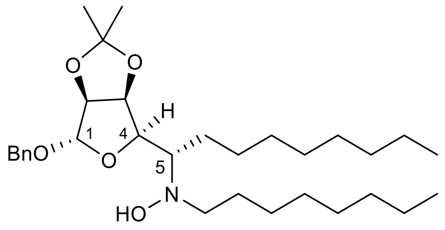

21

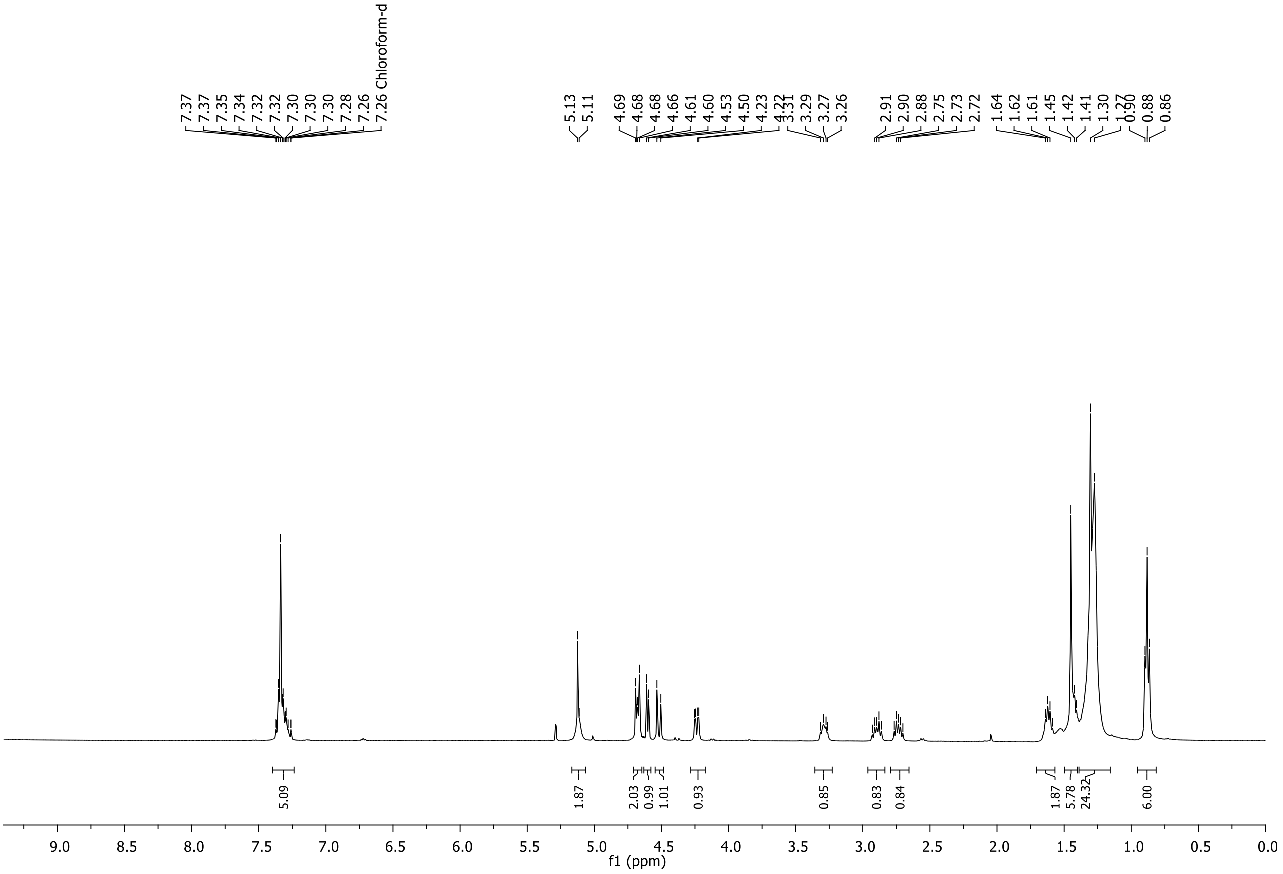

Figure S24. ^{1}H NMR spectrum of compound **21** (400 MHz, $CDCl_3$).

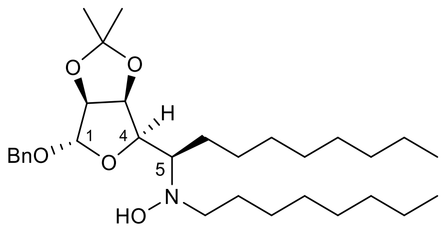

22

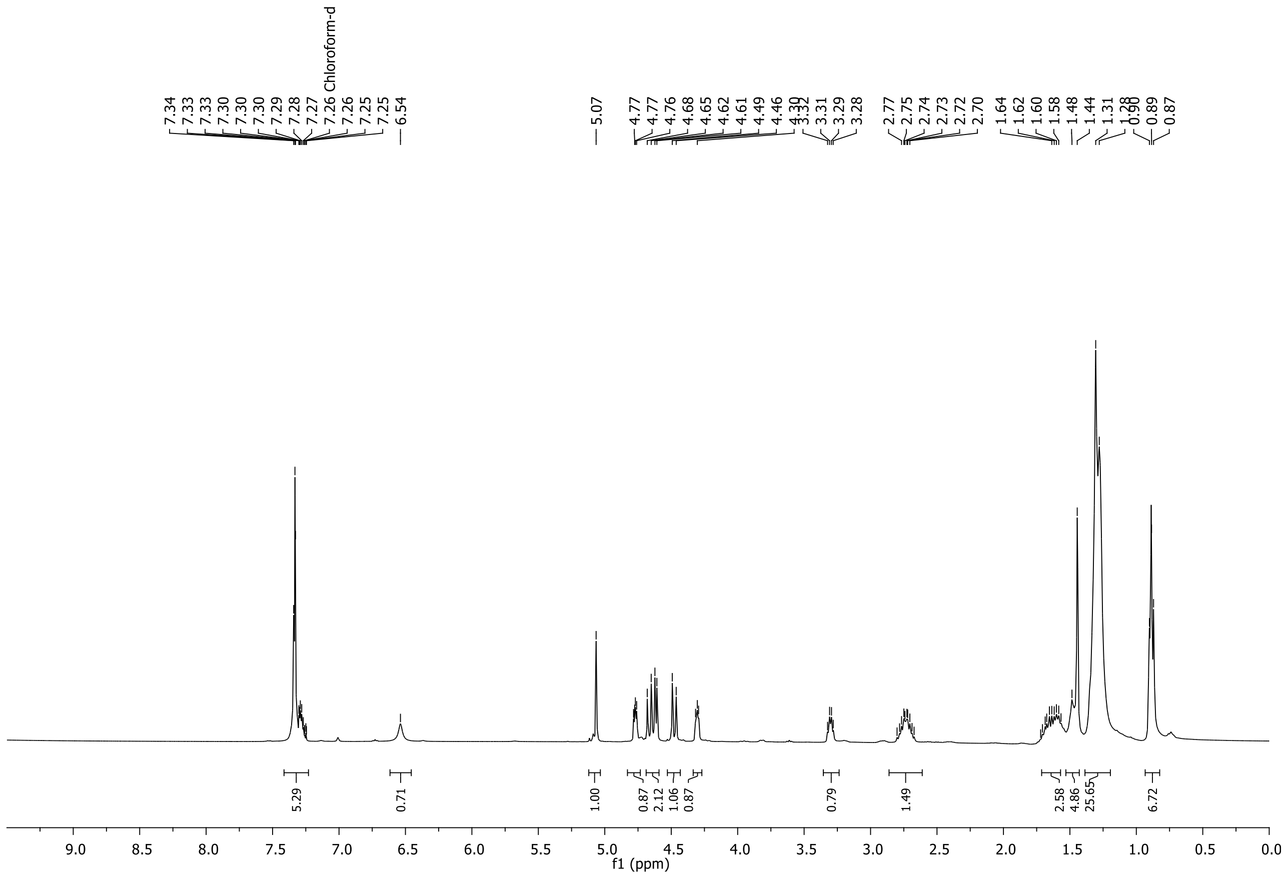

Figure S25. ^{1}H NMR spectrum of compound **22** (400 MHz, $CDCl_3$).

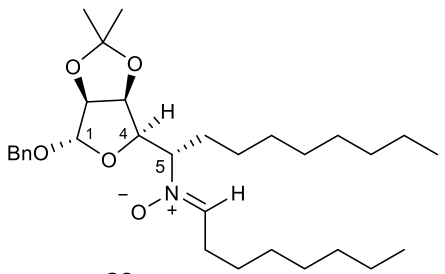

23

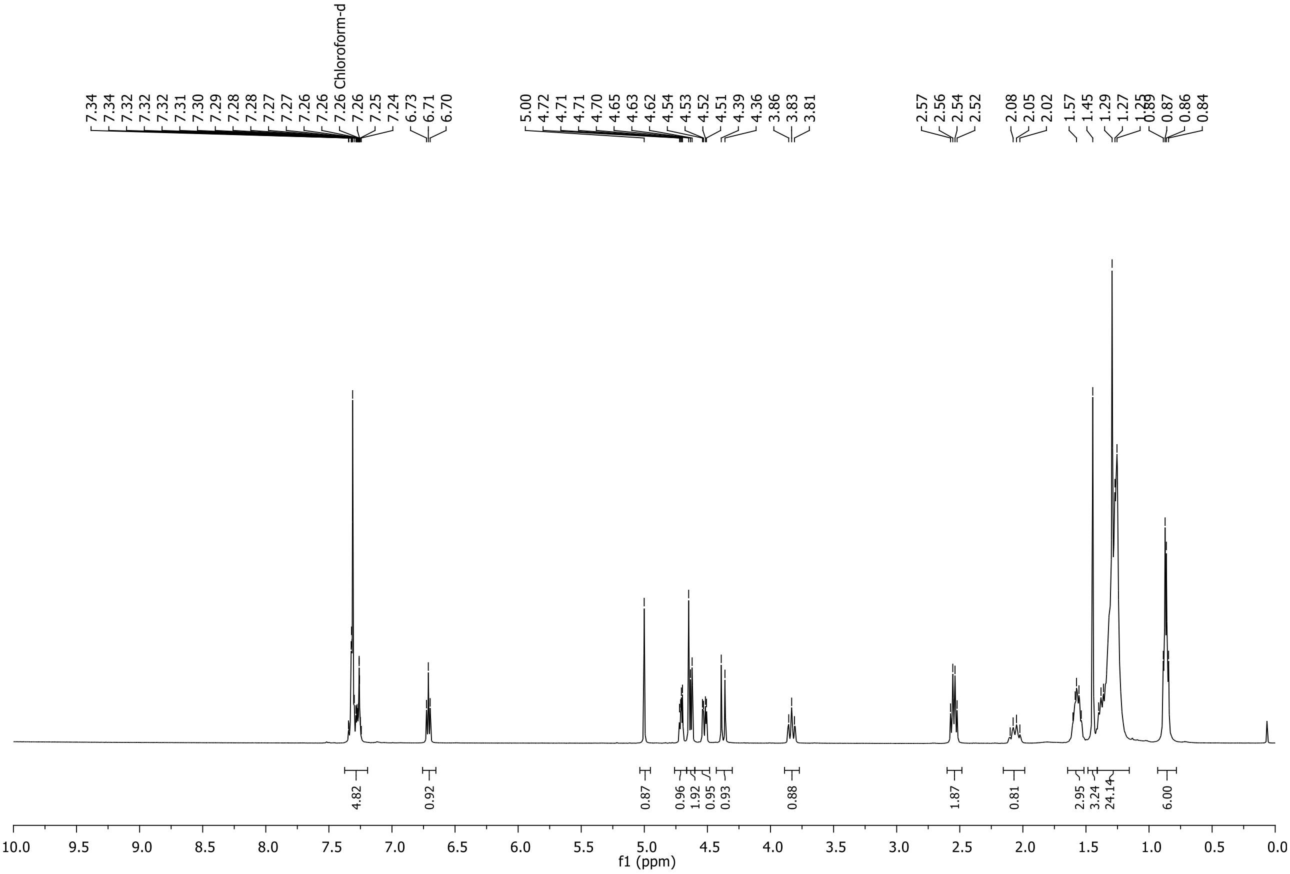

Figure S26. ^{1}H NMR spectrum of compound **23** (400 MHz, $CDCl_3$).

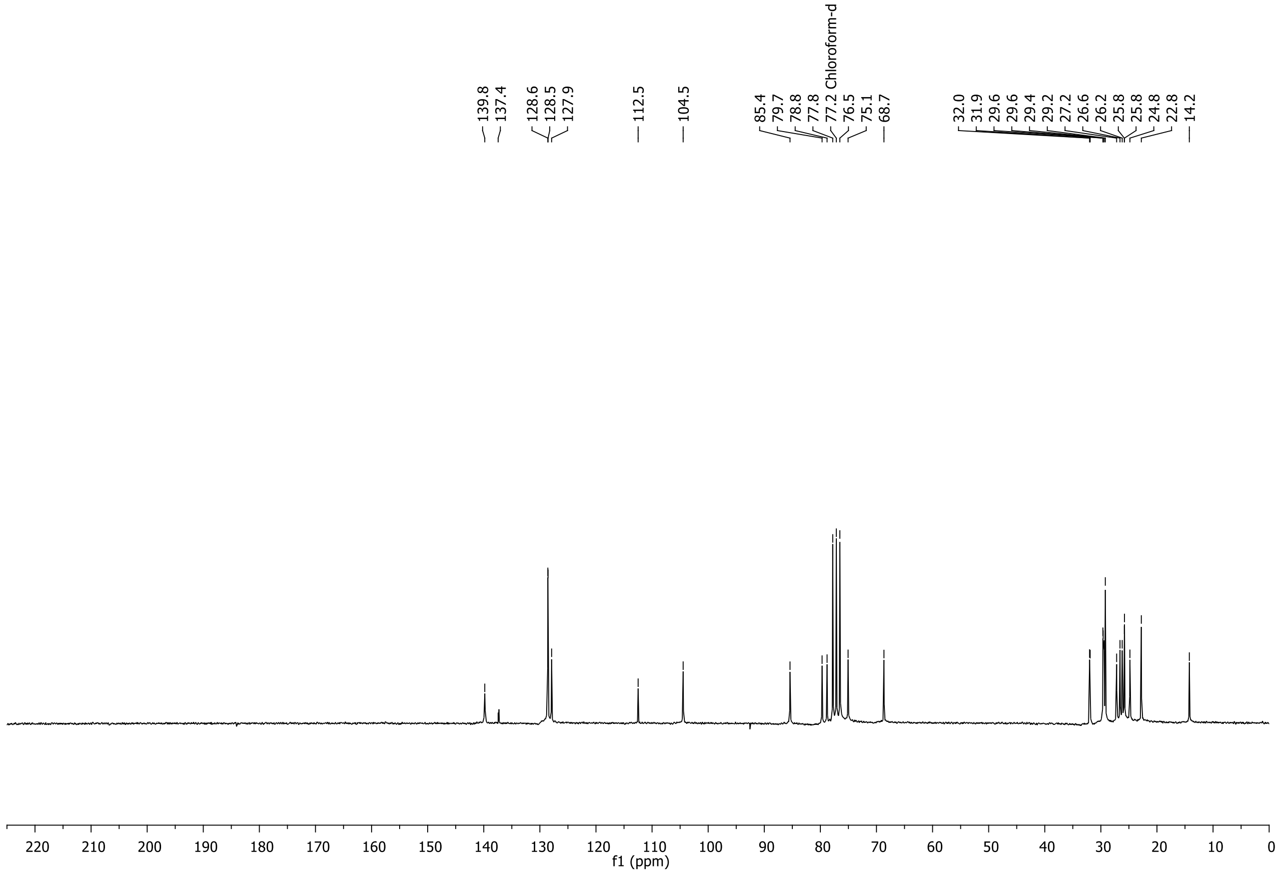

Figure S27. $^{13}C\{^1H\}$ NMR spectrum of compound **23** (50 MHz, $CDCl_3$).

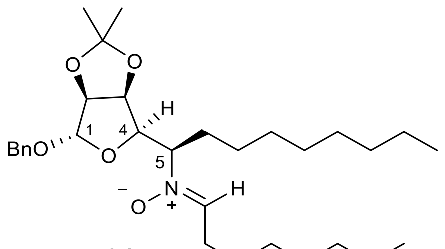

24

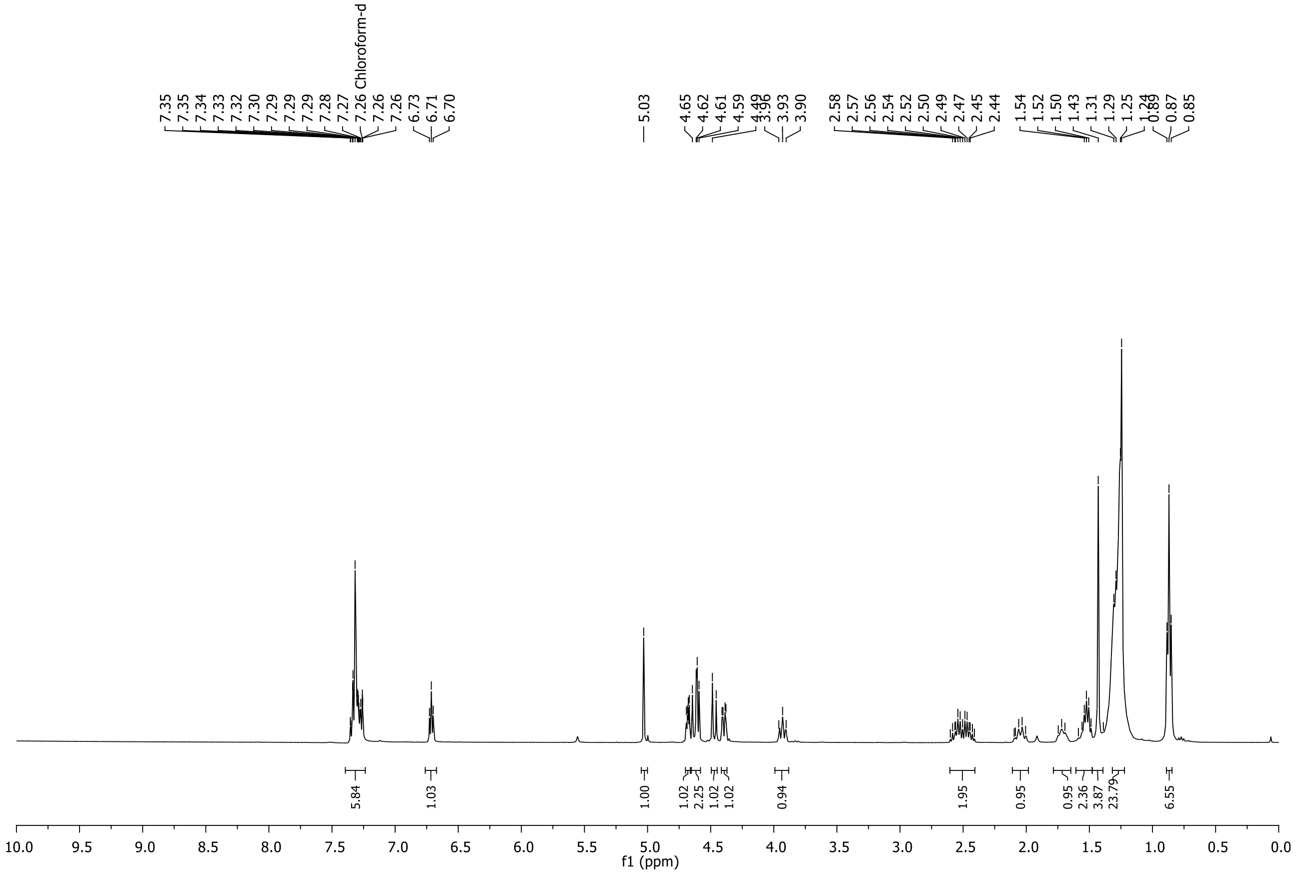

Figure S28. ^{1}H NMR spectrum of compound **24** (400 MHz, $CDCl_3$).

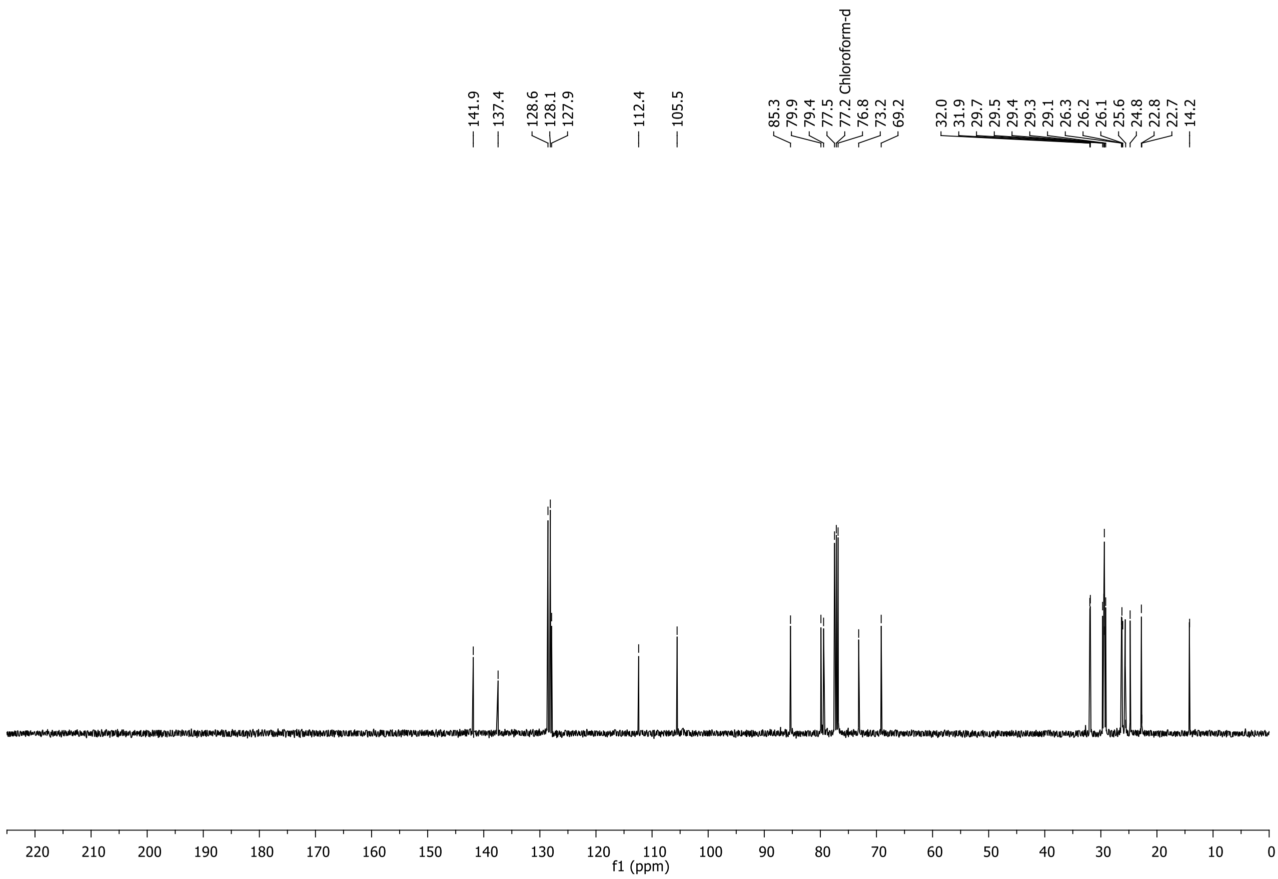

Figure S29. $^{13}C\{1H\}$ NMR spectrum of compound **24** (100 MHz, $CDCl_3$).

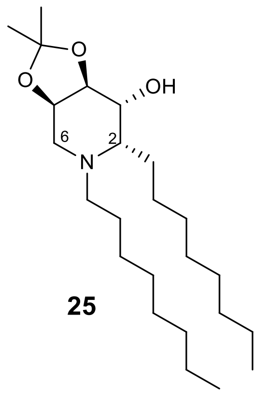

25

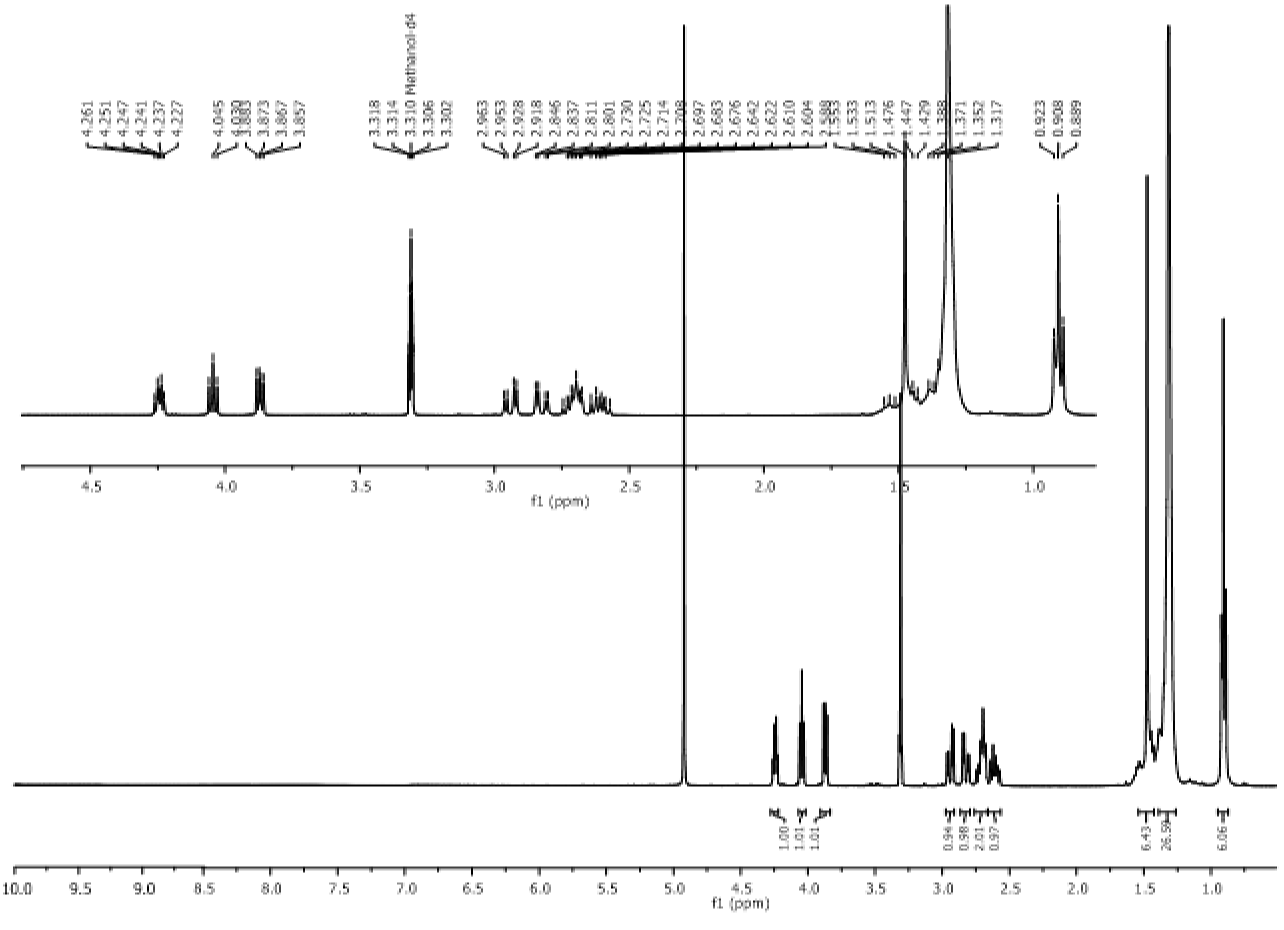

Figure S30. ^{1}H NMR spectrum of compound **25** (400 MHz, CD_3OD).

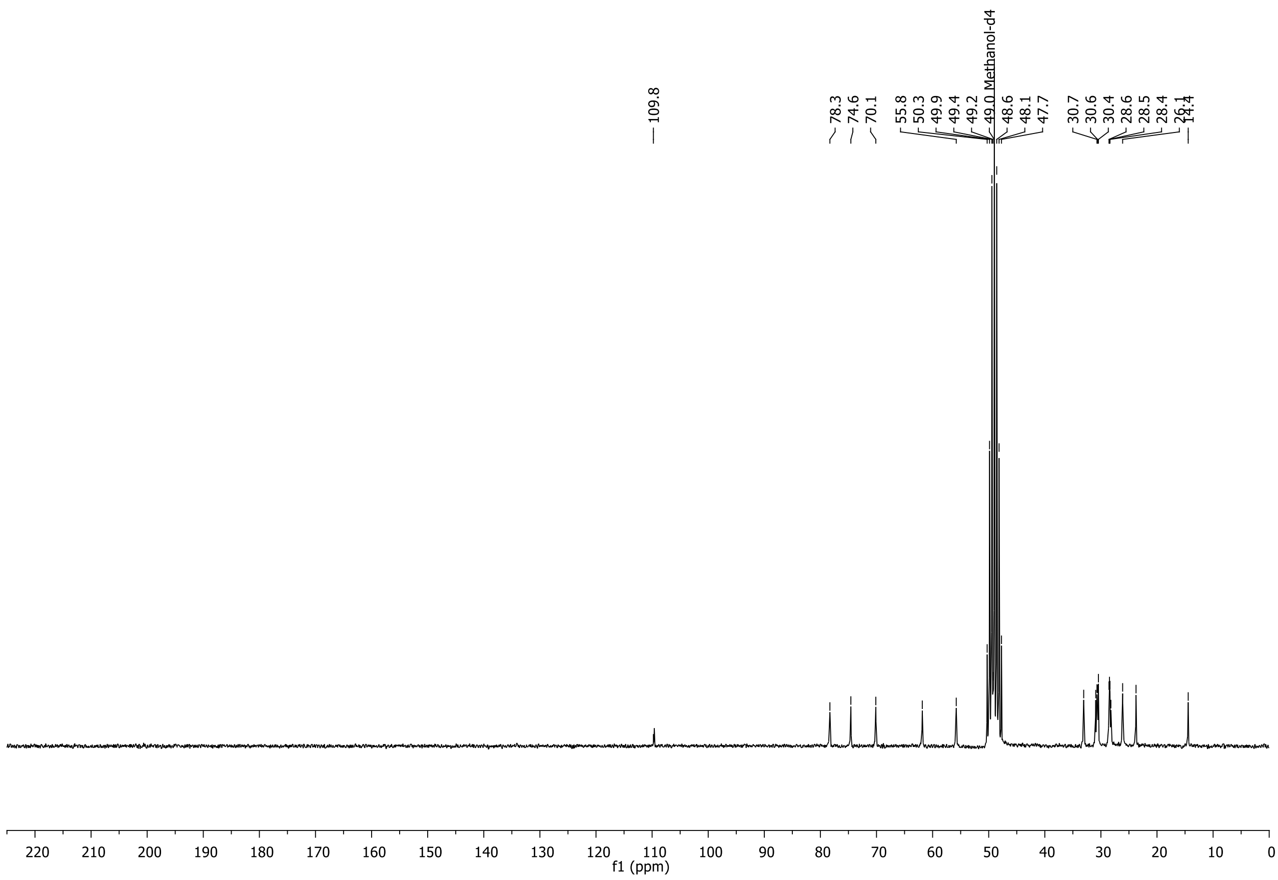

Figure S31. $^{13}C\{^1H\}$ NMR spectrum of compound **25** (50 MHz, CD_3OD).

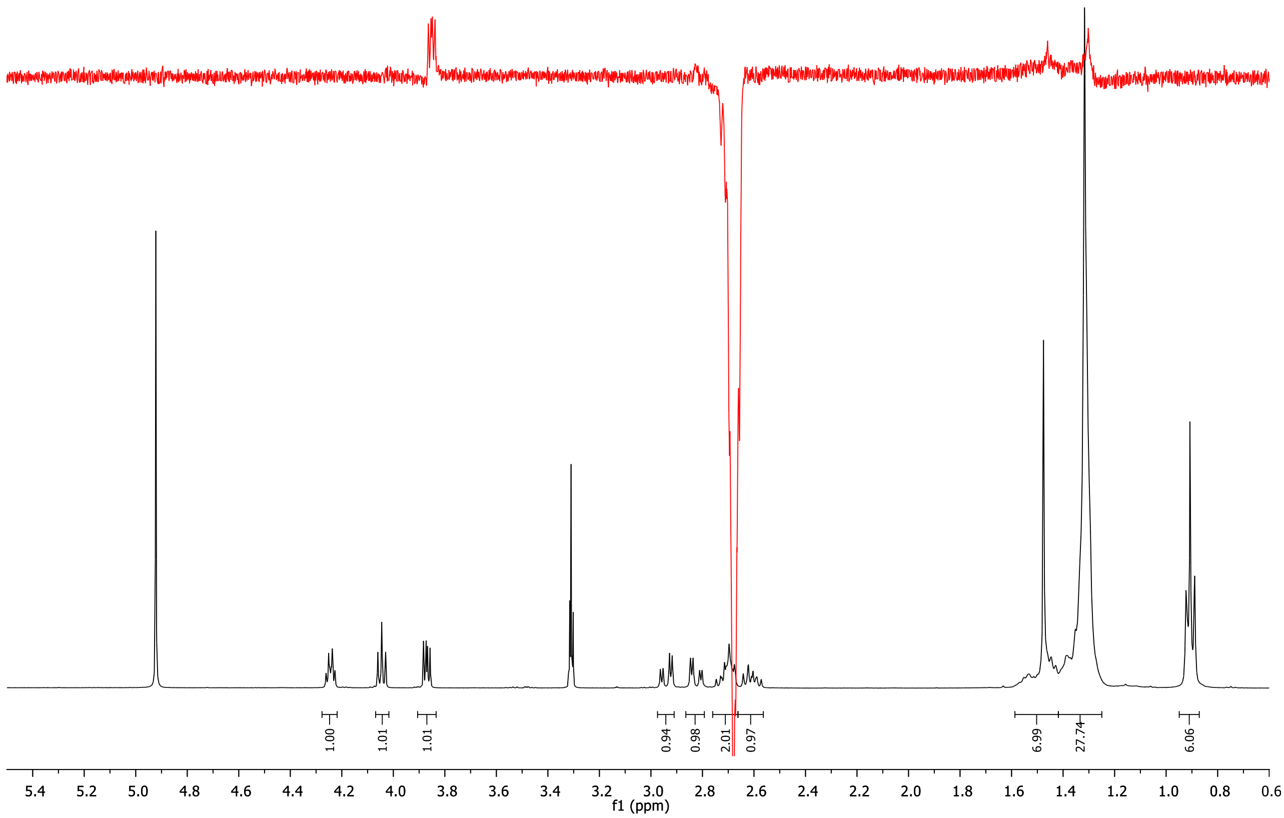

Figure S32. 1D NOESY spectra performed on compound **25**. Irradiation of 2-H.

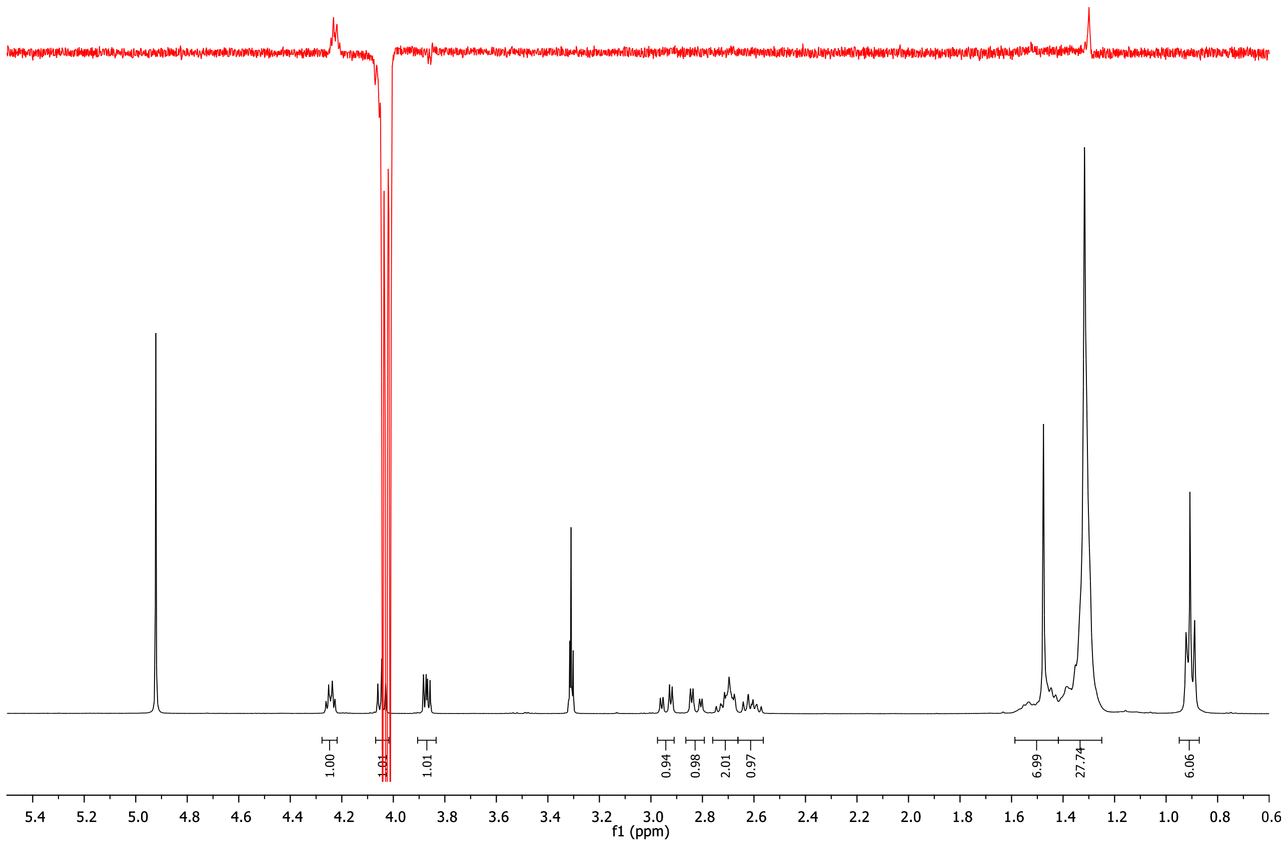

Figure S33. 1D NOESY spectra performed on compounds **25**. Irradiation of 4-H.

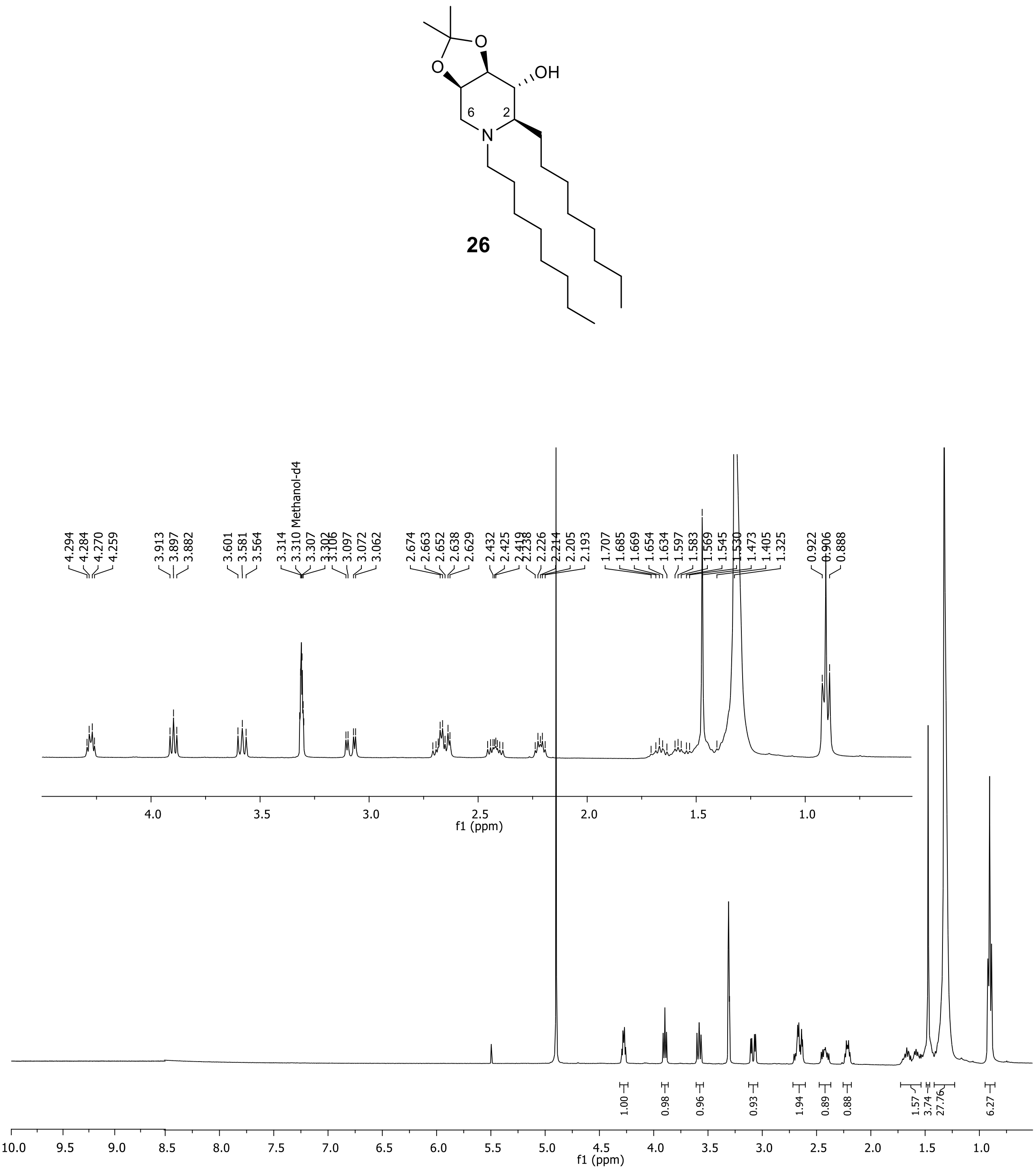

Figure S34. ^{1}H NMR spectrum of compound **26** (400 MHz, CD_3OD).

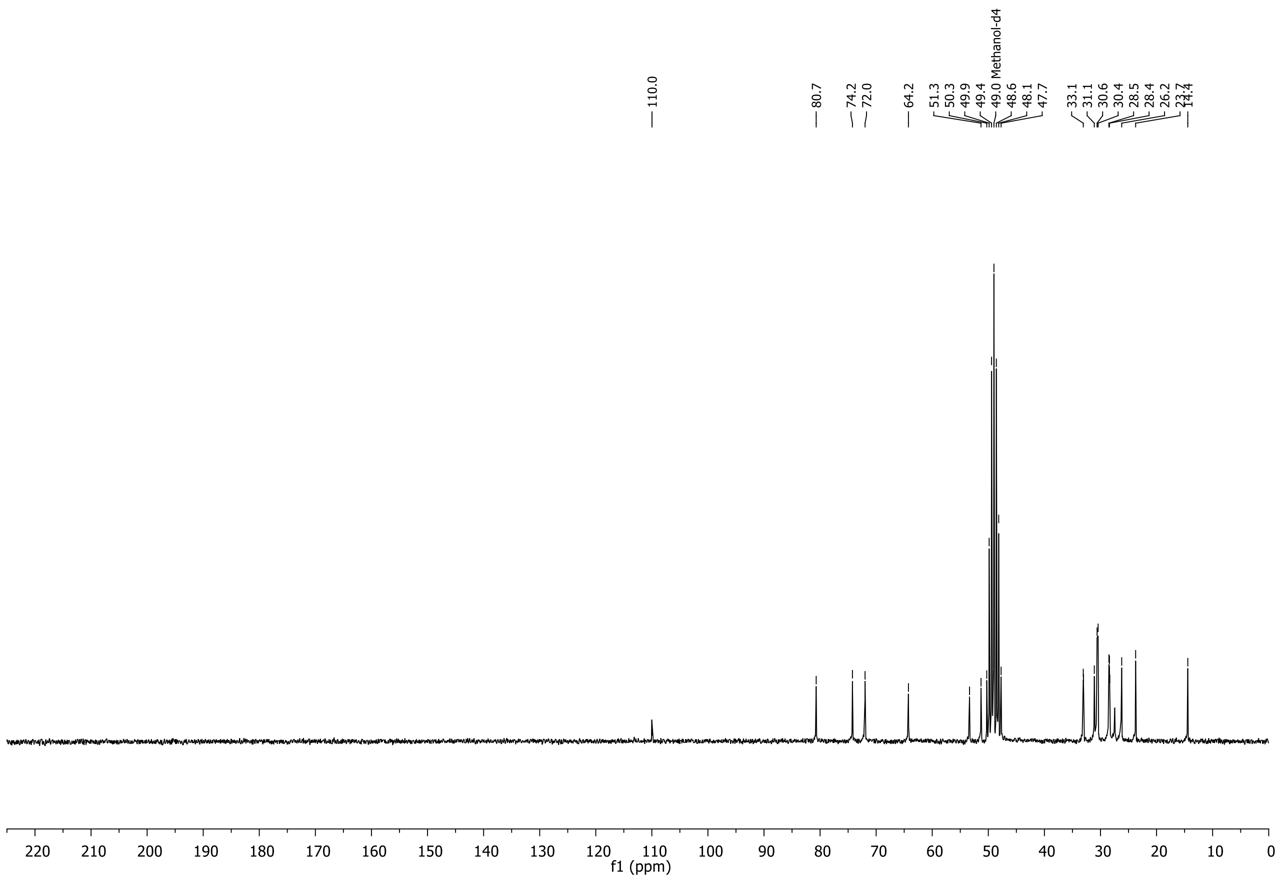

Figure S35. $^{13}C\{1H\}$ NMR spectrum of compound **26** (50 MHz, CD_3OD).

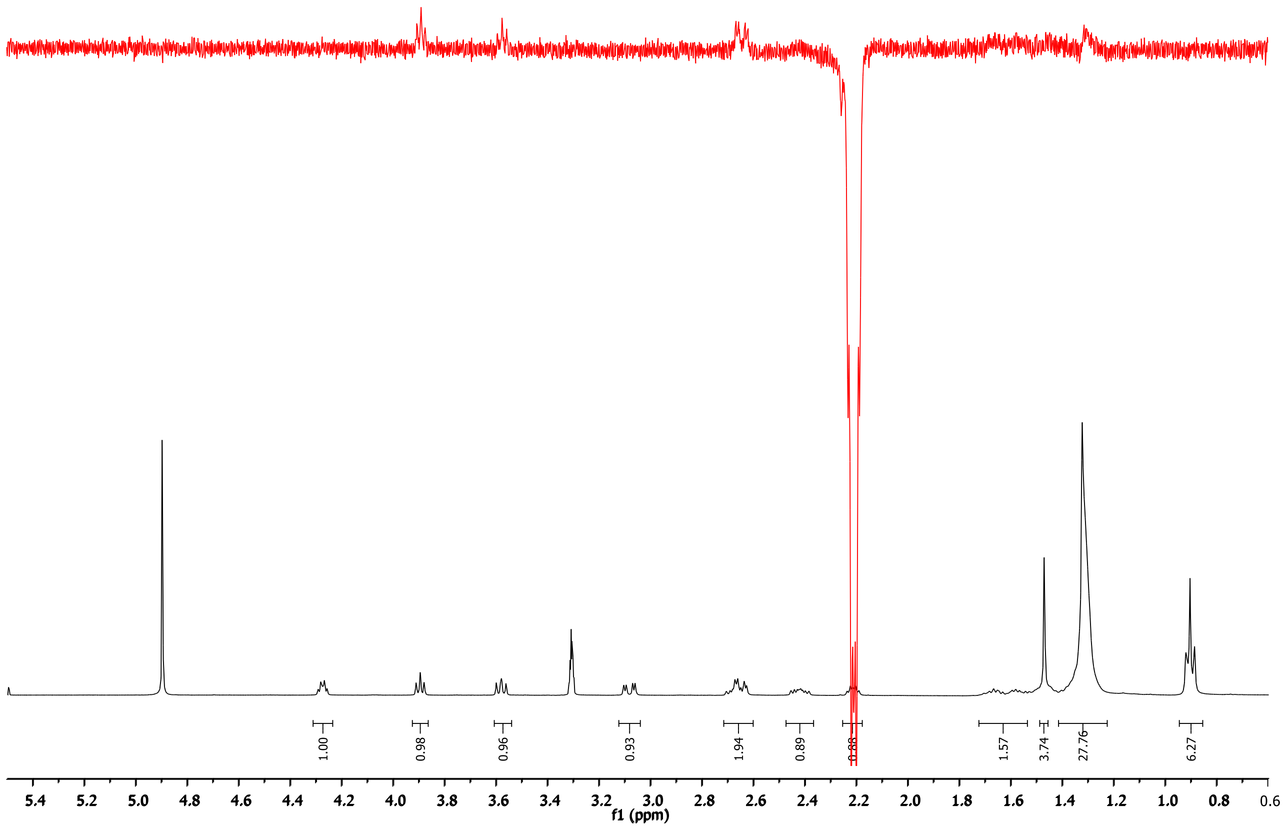

Figure S36. 1D NOESY spectra performed on compound **26**. Irradiation of 2-H gave a NOE at 4-H and 6-H_b.

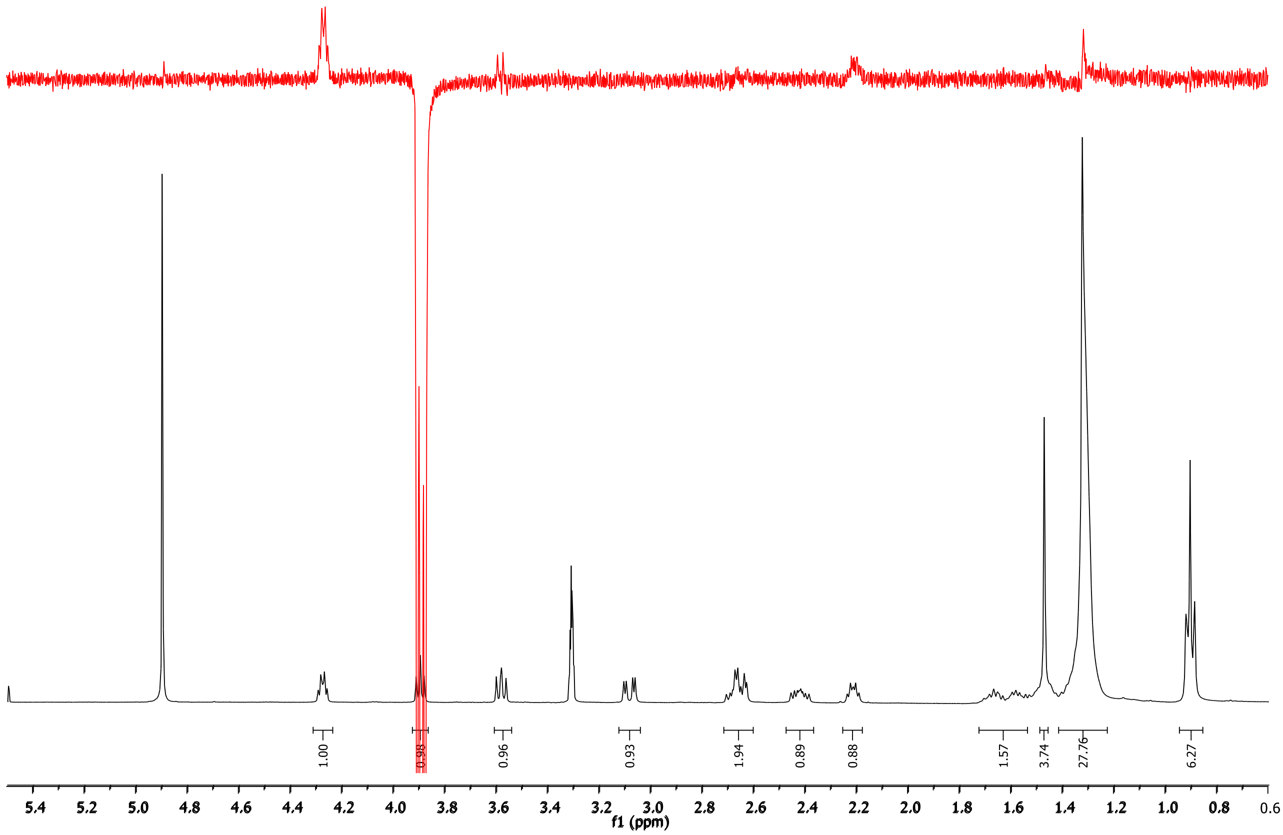

Figure S37. 1D NOESY spectra performed on compounds **26**. Irradiation of 4-H gave a NOE at 2-H and 6-H_b.

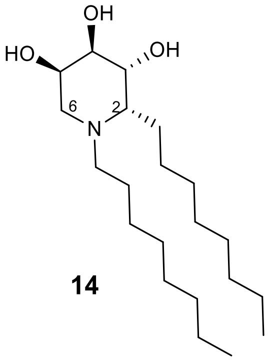

14

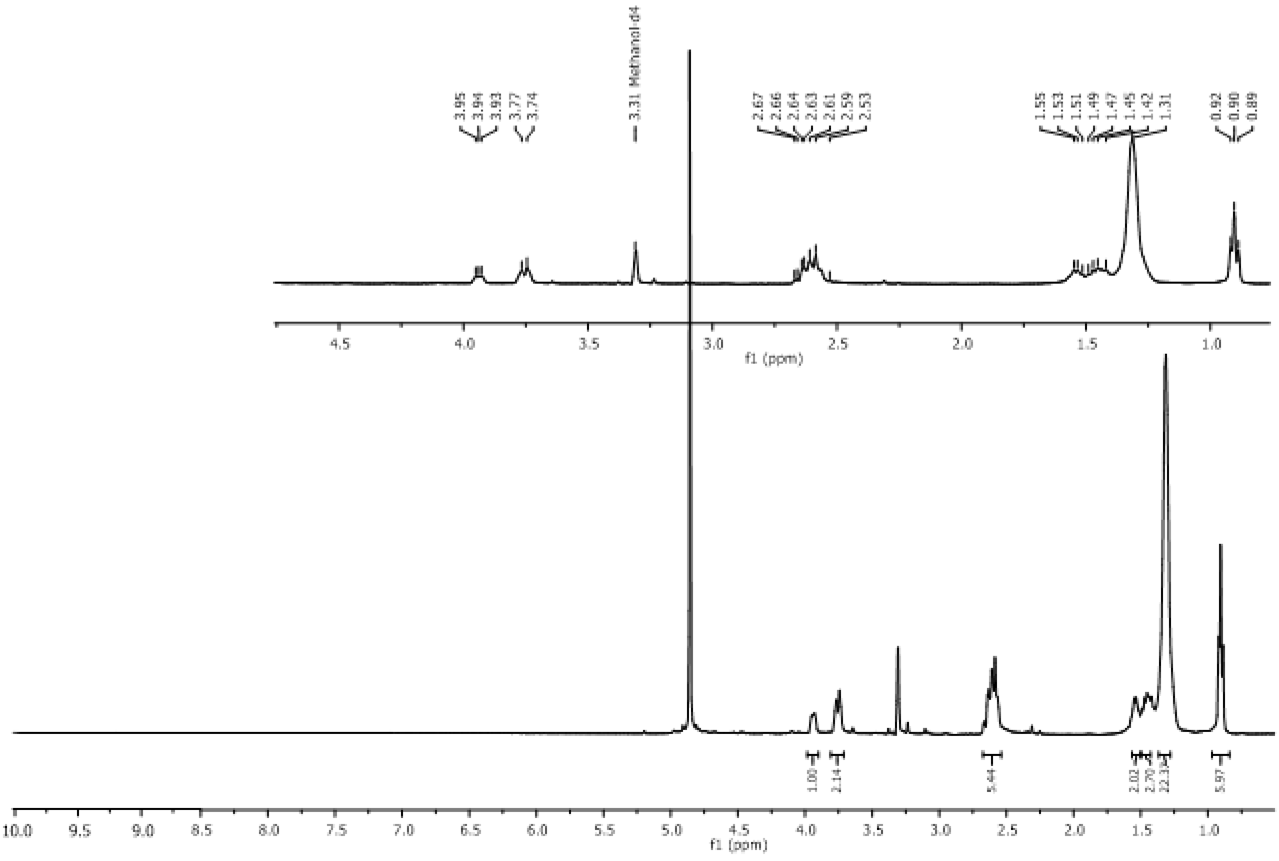

Figure S38. ^{1}H NMR spectrum of compound **14** (400 MHz, CD_3OD).

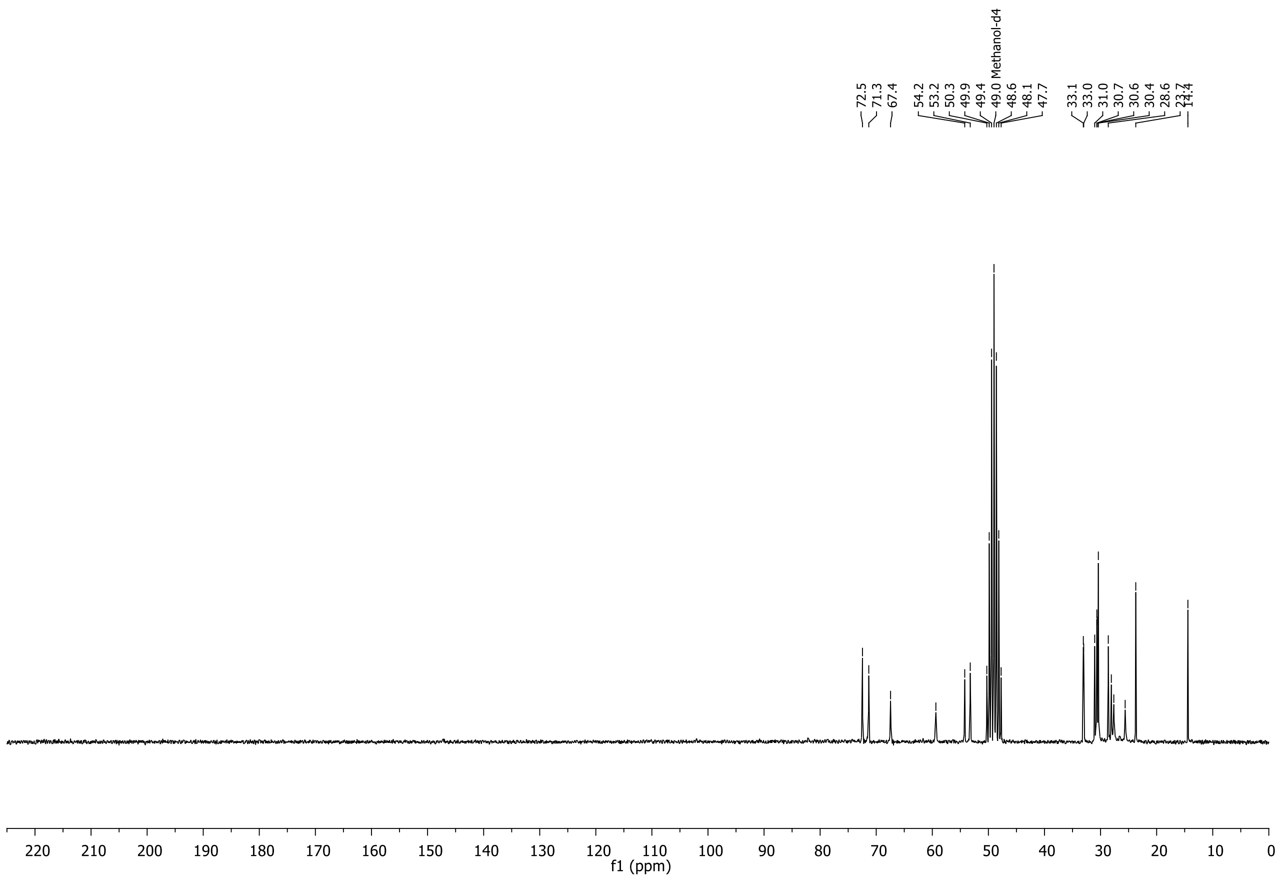

Figure S39. $^{13}C\{1H\}$ NMR spectrum of compound **14** (50 MHz, CD_3OD).

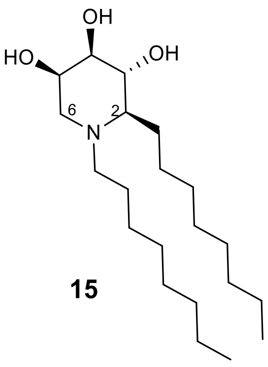

15

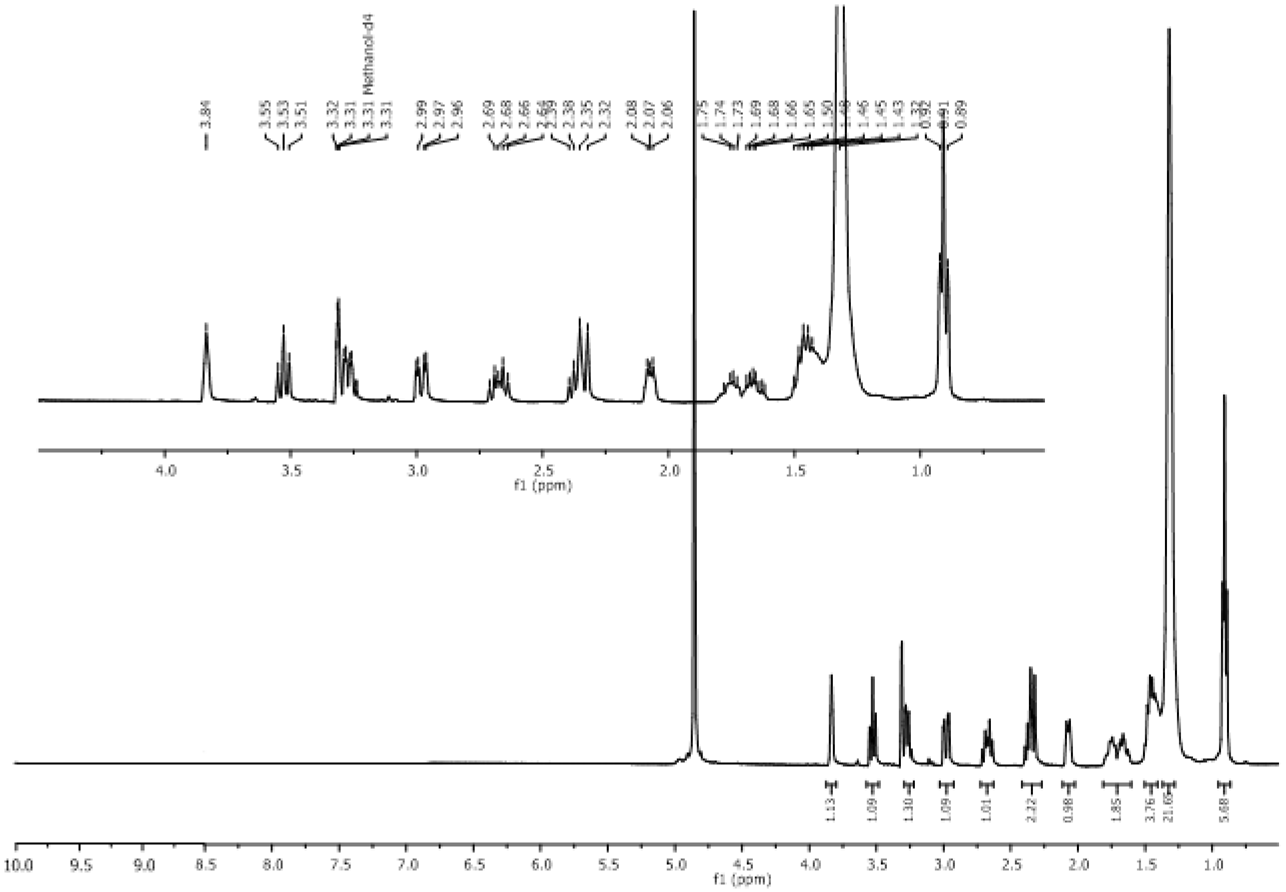

Figure S40. ^{1}H NMR spectrum of compound **15** (400 MHz, CD_3OD).

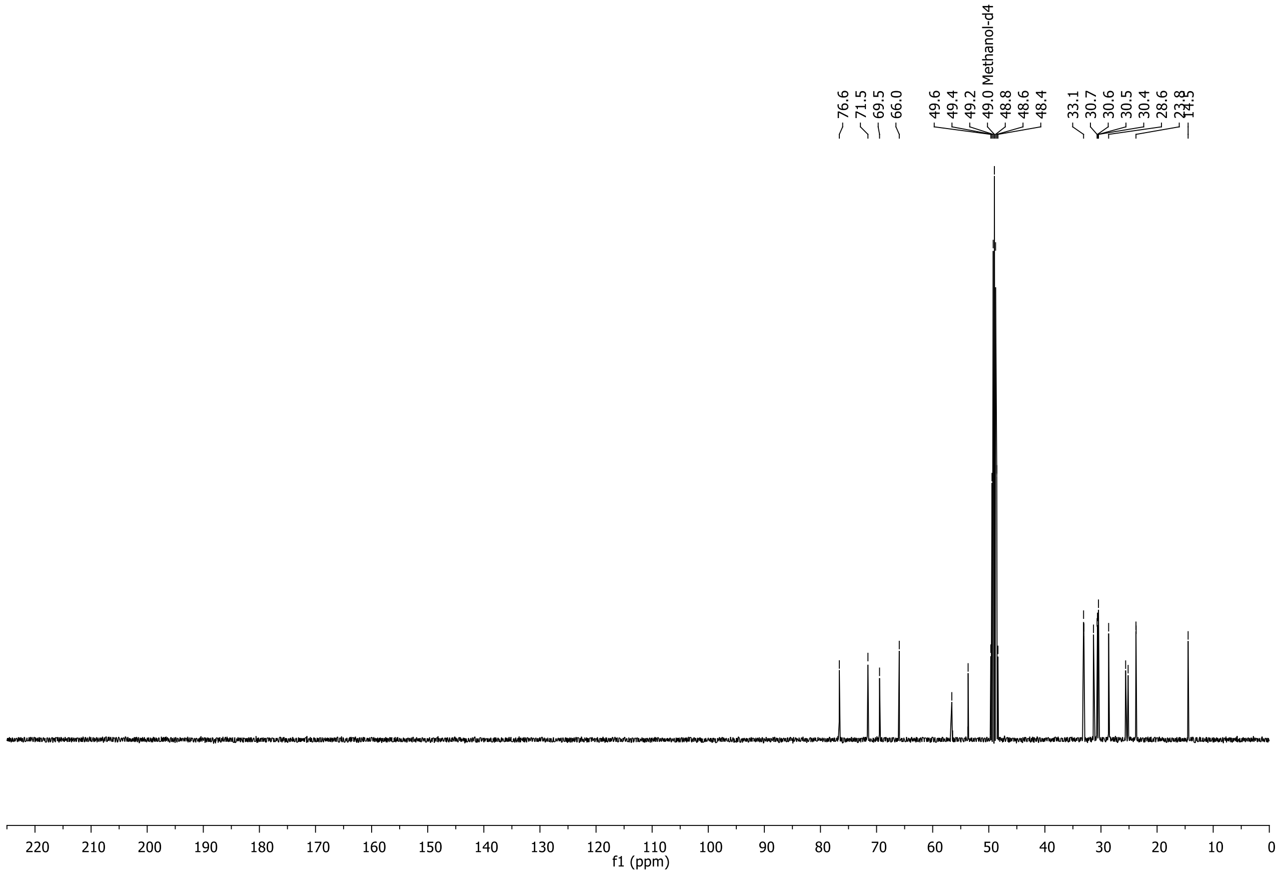

Figure S41. $^{13}C\{^1H\}$ NMR spectrum of compound **15** (100 MHz, CD_3OD).

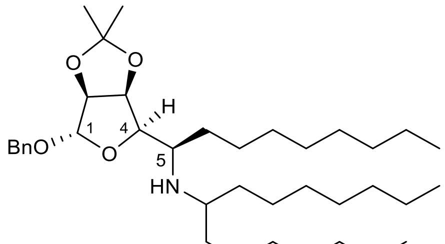

31

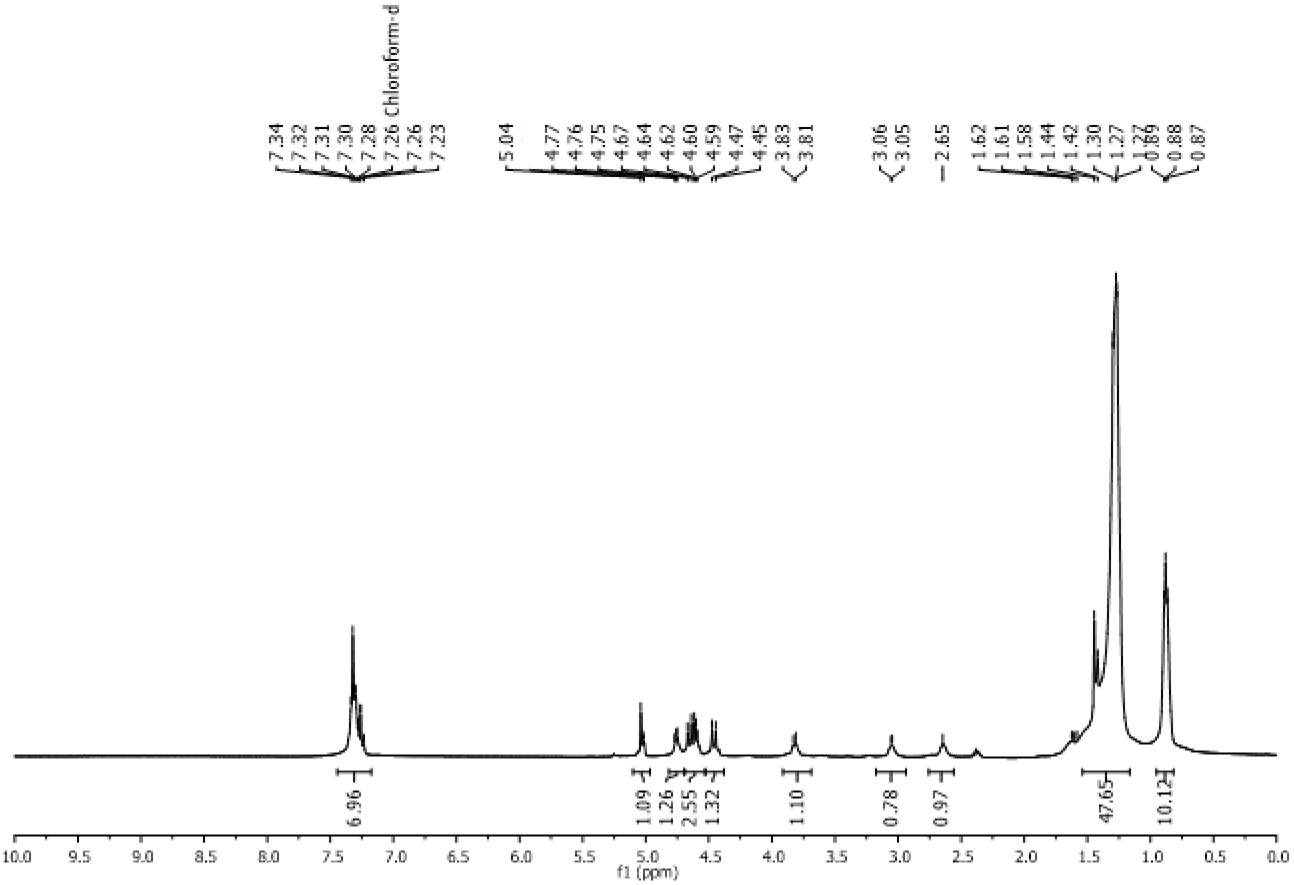

Figure S42. ^{1}H NMR spectrum of compound **31** (400 MHz, $CDCl_3$).

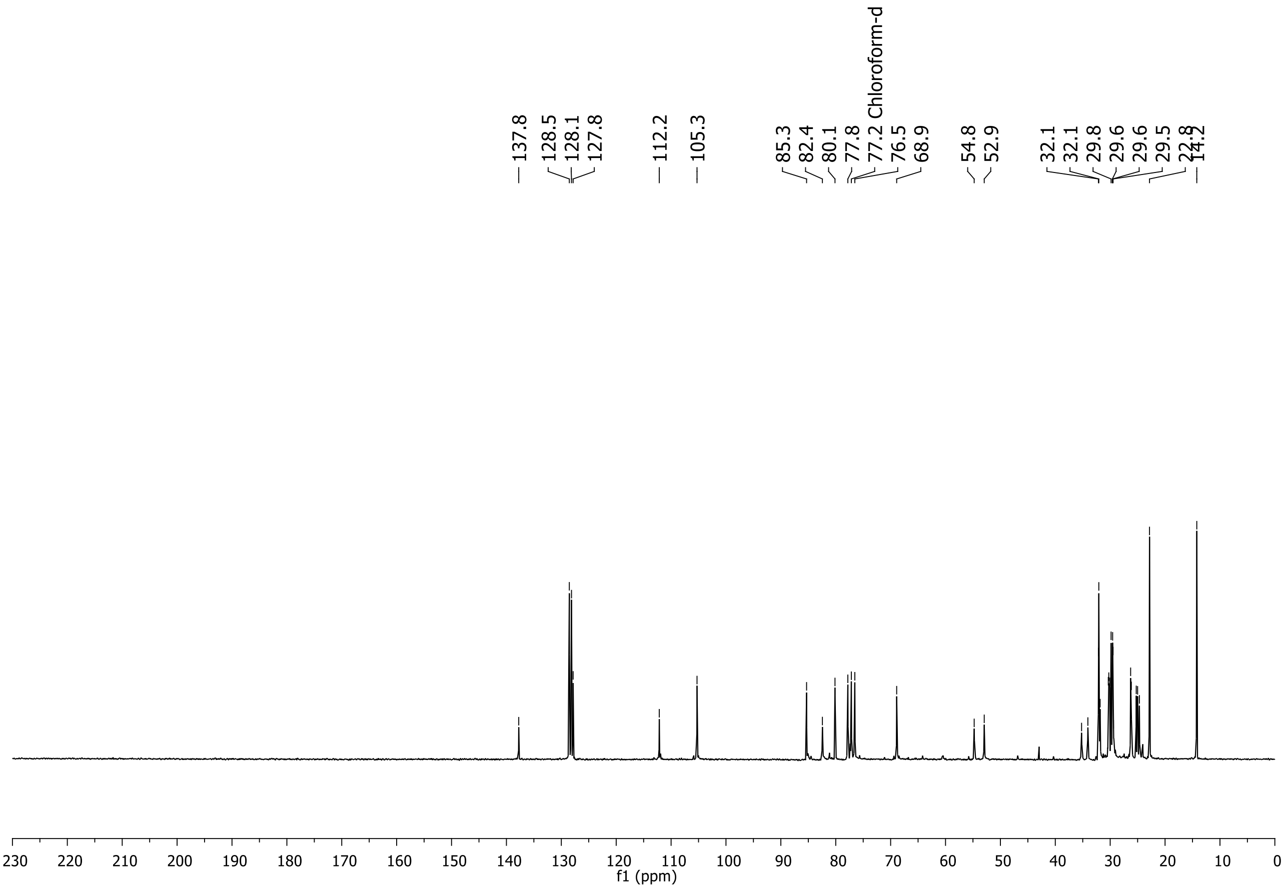

Figure S43. $^{13}C\{1H\}$ NMR spectrum of compound **31** (50 MHz, $CDCl_3$).

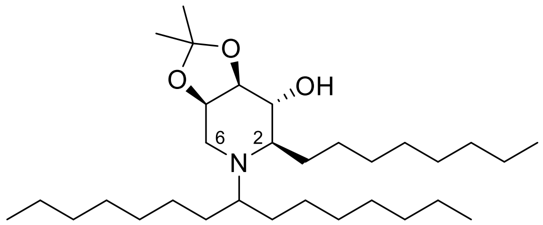

32

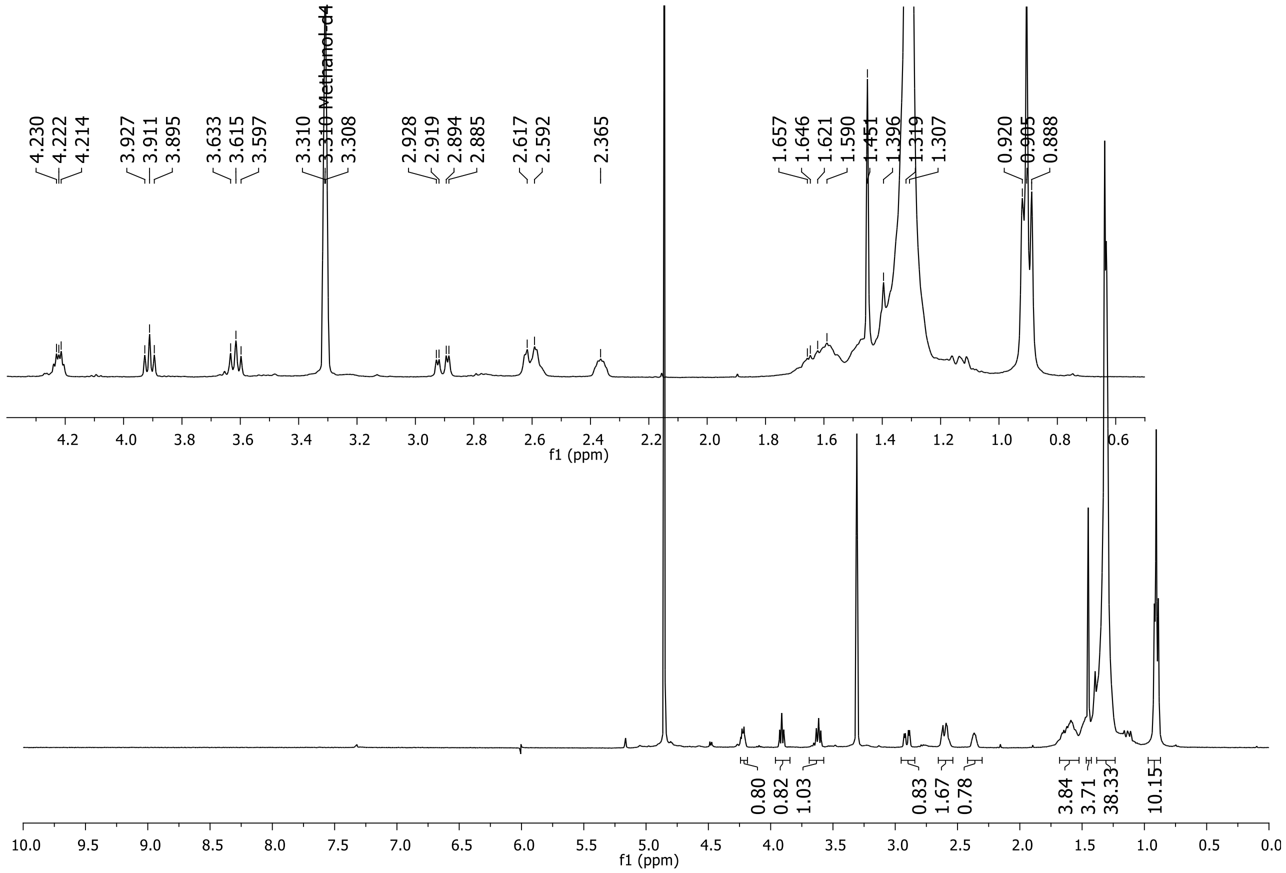

Figure S44. ^{1}H NMR spectrum of compound **32** (400 MHz, CD_3OD).

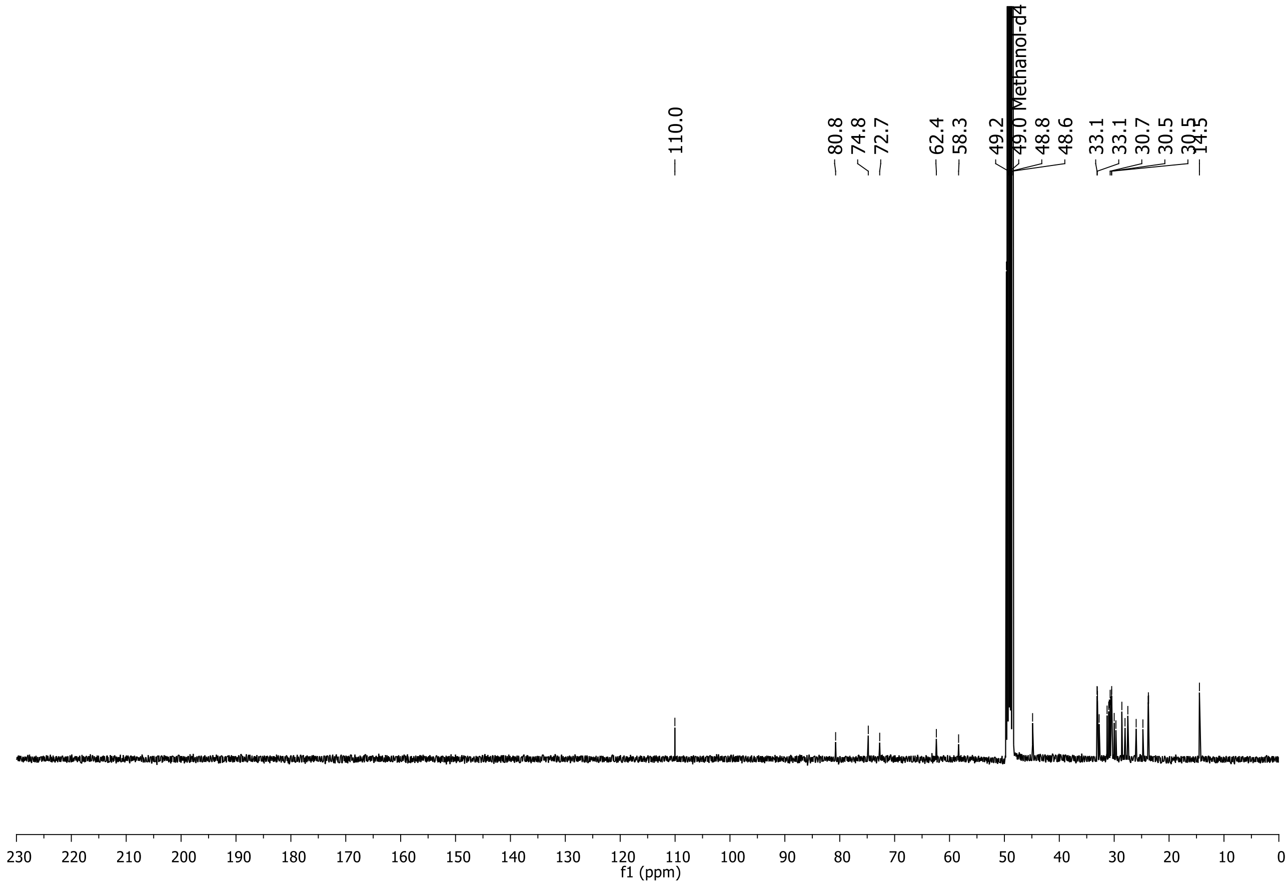

Figure S45. $^{13}C\{^{1}H\}$ NMR spectrum of compound **32** (100 MHz, CD_3OD).

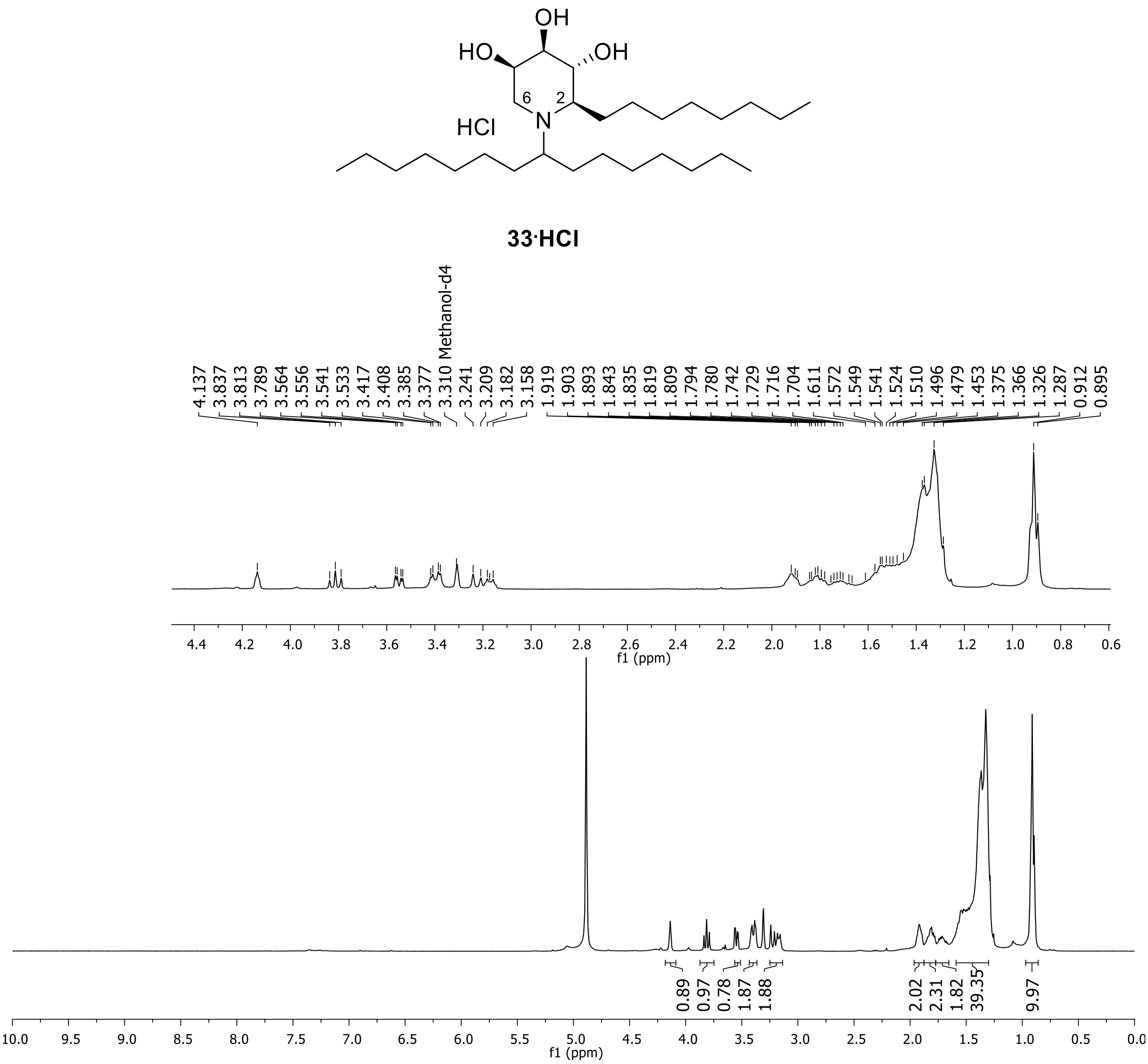

Figure S46. ^{1}H NMR spectrum of compound **33·HCl** (400 MHz, CD_3OD).

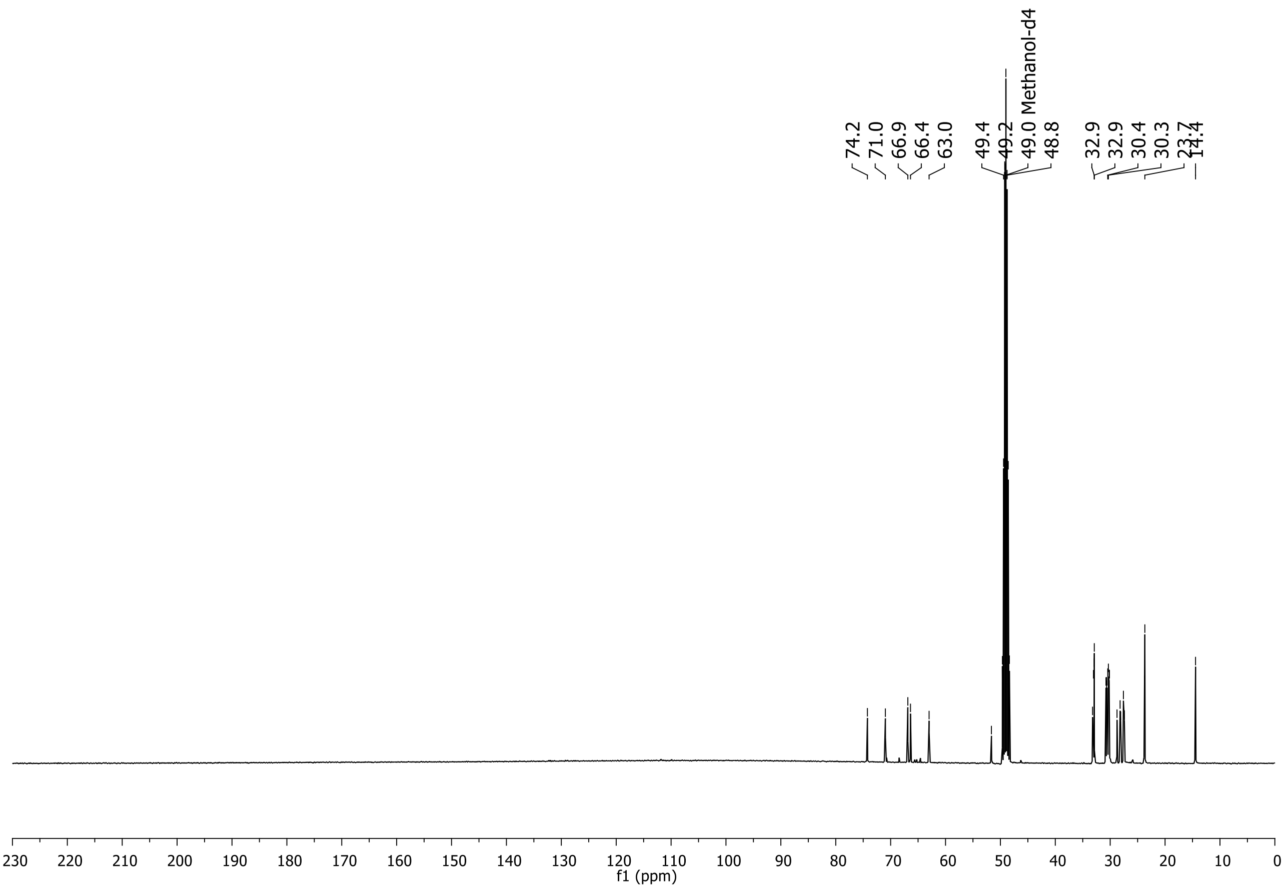

Figure S47. $^{13}C\{1H\}$ NMR spectrum of compound **33·HCl** (100 MHz, CD_3OD).

Biological screening towards commercial glycosidases

	% Inhibition at 0.1 mM	
	**14**	**15**
α-L-fucosidase EC 3.2.1.51<br>Homo sapiens	-	-
α-galactosidase EC 3.2.1.22<br>coffee beans	-	-
β-galactosidase EC 3.2.1.23<br>*Escherichia coli*	-	-
Aspergillus oryzae	-	-
α-glucosidase EC 3.2.1.20<br>yeast	-	-
rice	-	-
amyloglucosidase EC 3.2.1.3<br>*Aspergillus niger*	-	-
β-glucosidase EC 3.2.1.21<br>almonds	30±2	-
α-mannosidase EC 3.2.1.24<br>Jack beans	-	-
β-mannosidase EC 3.2.1.25<br>snail	-	-
β-*N*-acetylglucosaminidase EC 3.2.1.52<br>Jack beans	-	-
bovine kidney	-	-

"-": no inhibition (or less than 15%) detected.

Percentage of GCase inhibition of compounds 14, 15 and 33·HCl towards human GCase

Percentage of GCase inhibition of the whole collection of compounds in human leukocytes extracts incubated with iminosugars at 1 mM concentration.

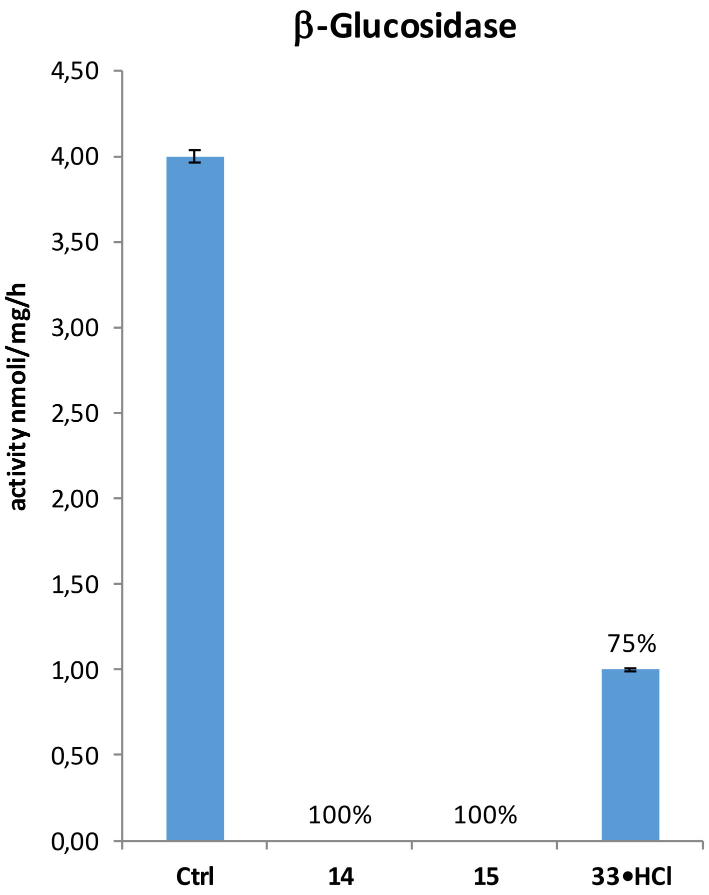

IC_{50} determination: The IC_{50} values of inhibitors against GCase were determined by measuring the initial hydrolysis rate with 4-methylumbelliferyl-β-D-glucoside (3.33 mM). Data obtained were fitted to the following equation using the Origin Microcal program.

$$\frac{Vi}{Vo} = \frac{Max - Min}{1 + \left(\frac{x}{IC_{50}}\right)^{slope}} + Min$$

where V_i/V_o, represent the ratio between the activity measured in the presence of the inhibitor (V_i) and the activity of the control without the inhibitor (V_0), "x" the inhibitor concentration, Max and Min, the maximal and minimal enzymatic activity observed, respectively.

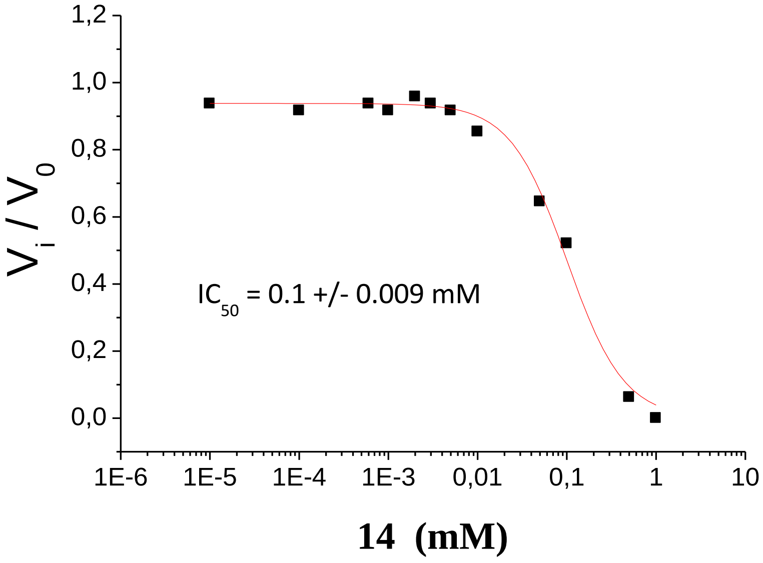
1,2
1,0
0,8
0,6
0,4
0,2
0,0
Vi / V0
IC50 = 0.1 +/- 0.009 mM
1E-6
1E-5
1E-4
1E-3
0,01
0,1
1
10
14 (mM)

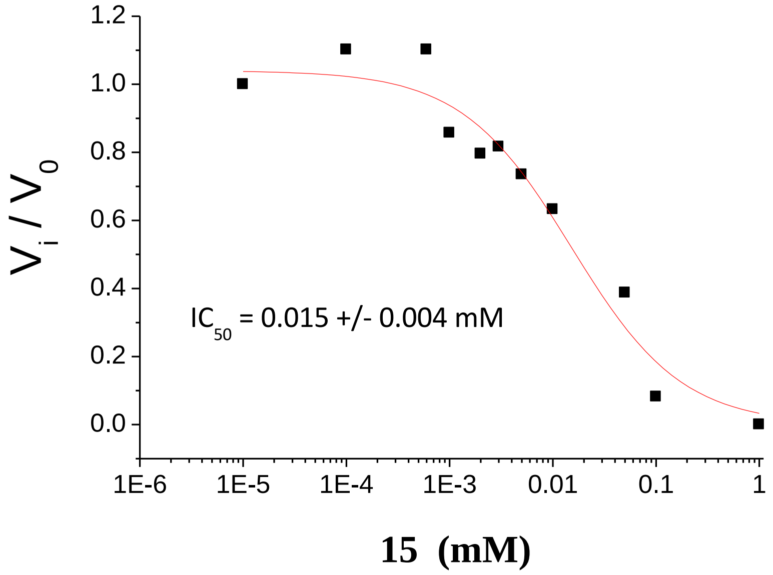
1.2
1.0
0.8
0.6
0.4
0.2
0.0
Vi / V0
IC50 = 0.015 +/- 0.004 mM
1E-6
1E-5
1E-4
1E-3
0.01
0.1
1
15 (mM)

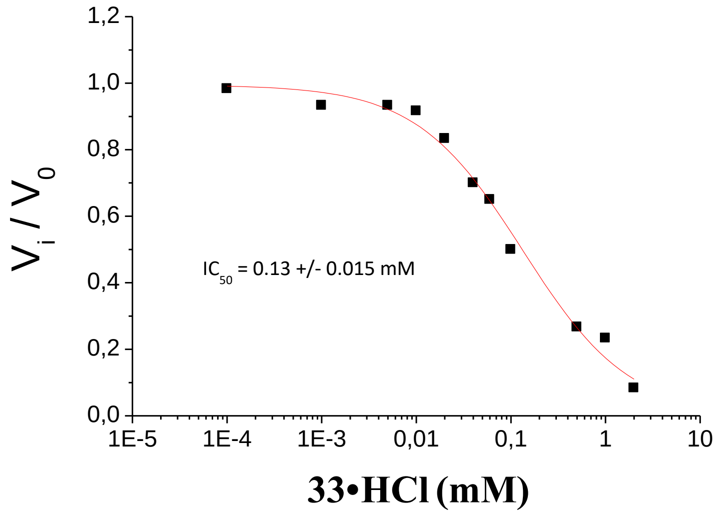

Selectivity of compounds 14, 15 and 33·HCl towards lysosomal enzymes.

The effect of 1 mM concentration of **14**, **15** and **33·HCl** was assayed towards six lysosomal glycosidases other than GCase, namely: α-mannosidase, β-mannosidase, α-galactosidase, β-galactosidase, α-fucosidase from leukocytes isolated from healthy donors (controls) and α-glucosidase from lymphocytes isolated from healthy donors' flesh blood (controls). Isolated leukocytes or lymphocytes were disrupted by sonication, and a micro BCA protein assay kit (Sigma–Aldrich) was used to determine the total protein amount for the enzymatic assay, according to the manufacturer instructions.

α-Mannosidase activity was measured in a flat-bottomed 96-well plate. Azasugar solution (3 μL), 4.29 μg/μL leukocytes homogenate 1:10 (7 μL), and substrate 4-methylumbelliferyl-α-D-mannopyranoside (2.67 mM, 20 μL, Sigma–Aldrich) in Na phosphate/citrate buffer (0.2:0.1, M/M, pH 4.0) containing sodium azide (0.02%) were incubated at 37 °C for 1 h. The reaction was stopped by addition of sodium carbonate (200 μL; 0.5M, pH 10.7) containing Triton X-100 (0.0025 %), and the fluorescence 4-methylumbelliferone released by α-mannosidase activity was measured in SpectraMax M2 microplate reader (λex=365 nm, λem=435 nm; Molecular Devices). Inhibition is given with respect to the control (without azasugar). Data are mean SD (n=3).

β-Mannosidase activity was measured in a flat-bottomed 96-well plate. Azasugar solution (3 μL), 4.29 μg/μL leukocytes homogenate 1:10 (7 μL), and substrate 4-methylumbelliferyl-β-D-mannopyranoside (1.33 mM, 20 μL, Sigma–Aldrich) in Na phosphate/citrate buffer (0.2:0.1, M/M, pH 4.0) containing sodium azide (0.02%) were incubated at 37 °C for 1h. The reaction was stopped by addition of sodium carbonate (200 μL; 0.5M, pH 10.7) containing Triton X-100 (0.0025 %), and the fluorescence of 4-methylumbelliferone released by β-mannosidase activity was measured in SpectraMax M2 microplate reader (λex=365 nm, λem=435 nm; Molecular Devices). Inhibition is given with respect to the control (without azasugar). Data are mean SD (n=3).

α-Galactosidase activity was measured in a flat-bottomed 96-well plate. Azasugar solution (3 μL), 4.29 μg/μL leukocytes homogenate 1:3 (7 μL), and substrate 4-methylumbelliferyl α-D-galactopyranoside (1.47 mM, 20 μL, Sigma–Aldrich) in acetate buffer (0.1 M, pH 4.5) containing sodium azide (0.02%) were incubated at 37 °C for 1 h. The reaction was stopped by addition of sodium carbonate (200 μL; 0.5M, pH 10.7) containing Triton X-100 (0.0025 %), and the fluorescence 4-methylumbelliferone released by α-galactosidase activity was measured in SpectraMax M2 microplate reader (λex=365 nm, λem=435 nm; Molecular Devices). Inhibition is given with respect to the control (without azasugar). Data are mean SD (n=3).

β- Galactosidase activity was measured in a flat-bottomed 96-well plate. Azasugar solution (3 μL), 4.29 μg/μL leukocytes homogenate 1:10 (7 μL), and substrate 4-methylumbelliferyl β-D-galactopyranoside (1.47 mM, 20

μL, Sigma–Aldrich) in acetate buffer (0.1M, pH 4.3) containing NaCl (0.1M) and sodium azide (0.02%) were incubated at 37 °C for 1 h. The reaction was stopped by addition of sodium carbonate (200 μL; 0.5M, pH 10.7) containing Triton X-100 (0.0025 %), and the fluorescence 4-methylumbelliferone released by β-galactosidase activity was measured in SpectraMax M2 microplate reader (λex=365 nm, λem=435 nm; Molecular Devices). Inhibition is given with respect to the control (without azasugar). Data are mean SD (n=3).

α-Fucosidase activity was measured in a flat-bottomed 96-well plate. Azasugar solution (3 μL), 4.29 μg/μL leukocytes homogenate 1:3 (7 μL), and substrate 4-methylumbelliferyl α-L-fucopyranoside (1.51 mM, 20 μL, Sigma–Aldrich) in Na phosphate/citrate buffer (0.2:0.1, M/M, pH 5.5) were incubated at 37 °C for 1 h. The reaction was stopped by addition of sodium carbonate (200 μL; 0.5M, pH 10.7) containing Triton X-100 (0.0025 %), and the fluorescence 4-methylumbelliferone released by α- fucosidase activity was measured in SpectraMax M2 microplate reader (λex=365 nm, λem=435 nm; Molecular Devices). Inhibition is given with respect to the control (without azasugar). Data are mean SD (n=3). S74

α-Glucosidase activity was measured in a flat–bottomed 96 well plate. Azasugar solution (3 μL), 4.29 μg/μL lymphocytes homogenate (7μL) and 20 μL of substrate solution of 4-methylumbelliferyl-α-D-glucopyranoside (Sigma-Aldrich) in Na acetate buffer (0.2 M, pH 4.0) were incubated for 1 h at 37 °C. The reaction was stopped by the addition of a solution of sodium carbonate (200 μL; 0.5M, pH 10.7) containing Triton X-100 (0.0025 %), and the fluorescence of 4-methylumbelliferone released by α-glucosidase activity was measured in SpectraMax M2 microplate reader (λex=365 nm, λem=435 nm; Molecular Devices). Inhibition is given with respect to the control (without azasugar). Data are means of 3 values.

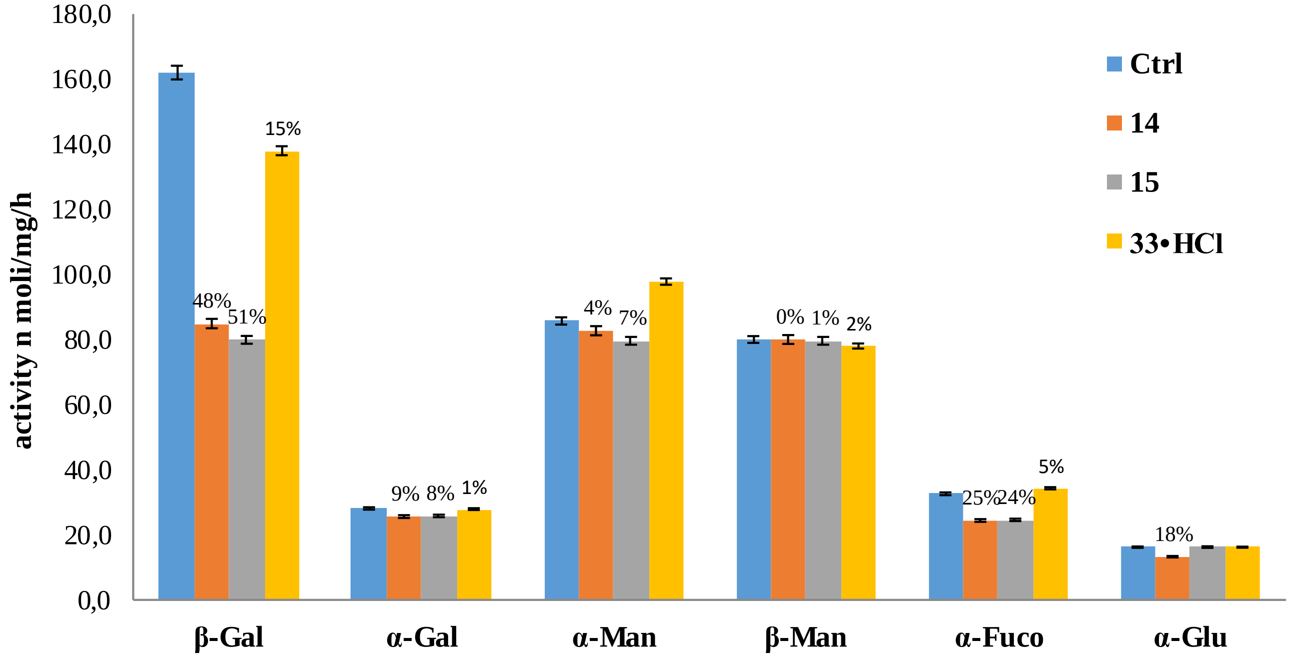

Figure S48. Activity of a panel of 6 human lysosomal glycosidases in the presence of compounds **14, 15** and **33·HCl** (1 mM). The corresponding calculated percentage of inhibition is indicated above each bar.

Cytotoxicity test of compound 33·HCl

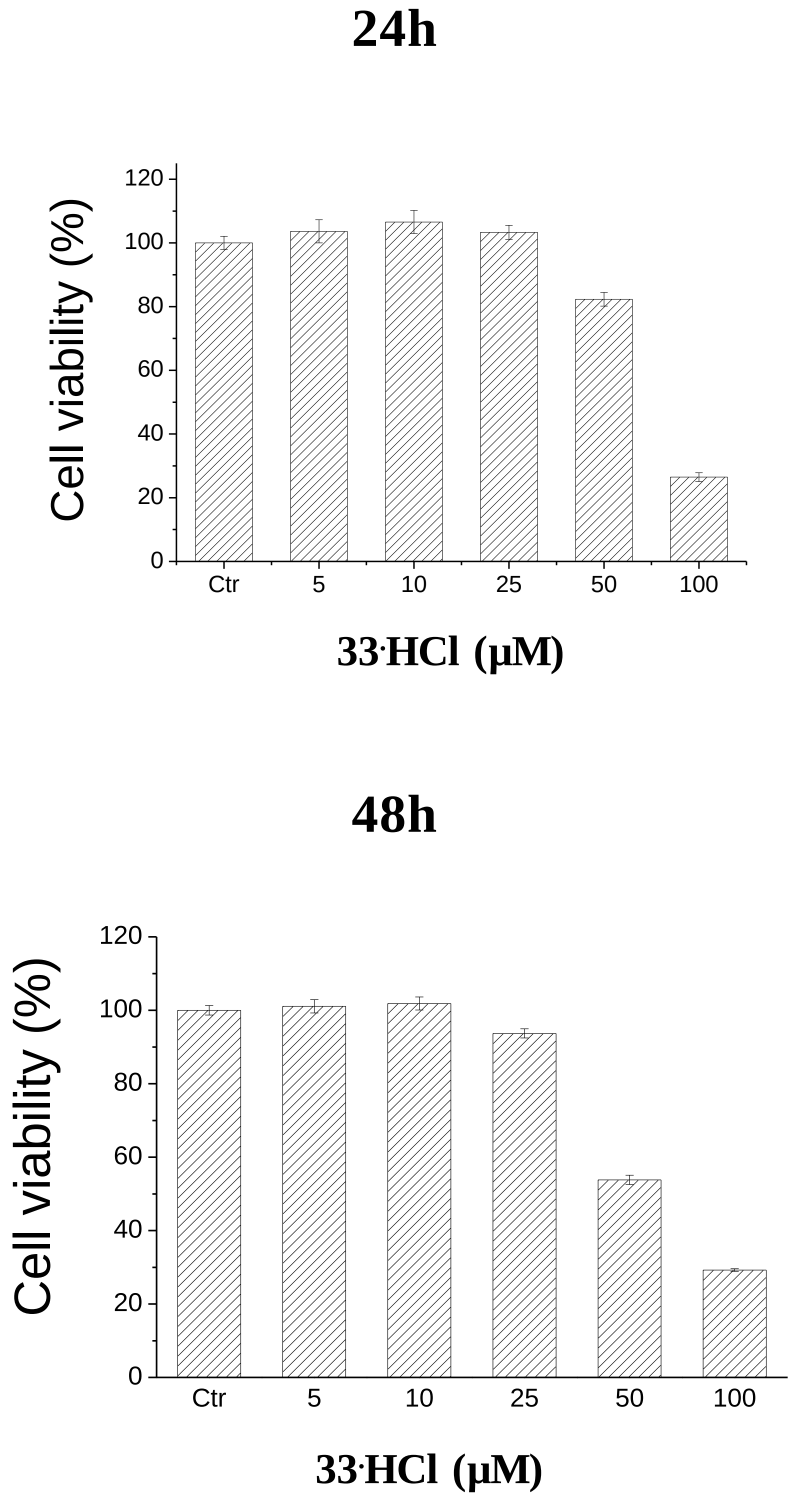

Figure S49. Viability assay. Wild-type fibroblasts were incubated for 24 and 48 h in the presence of compound **33·HCl** at different concentrations. After this time, the viability of cells was evaluated using MMT assay. Obtained values were normalized with respect to control experiments. Data reported represent the mean value ± S.E.M. (n = 8).

Pharmacological chaperoning activity

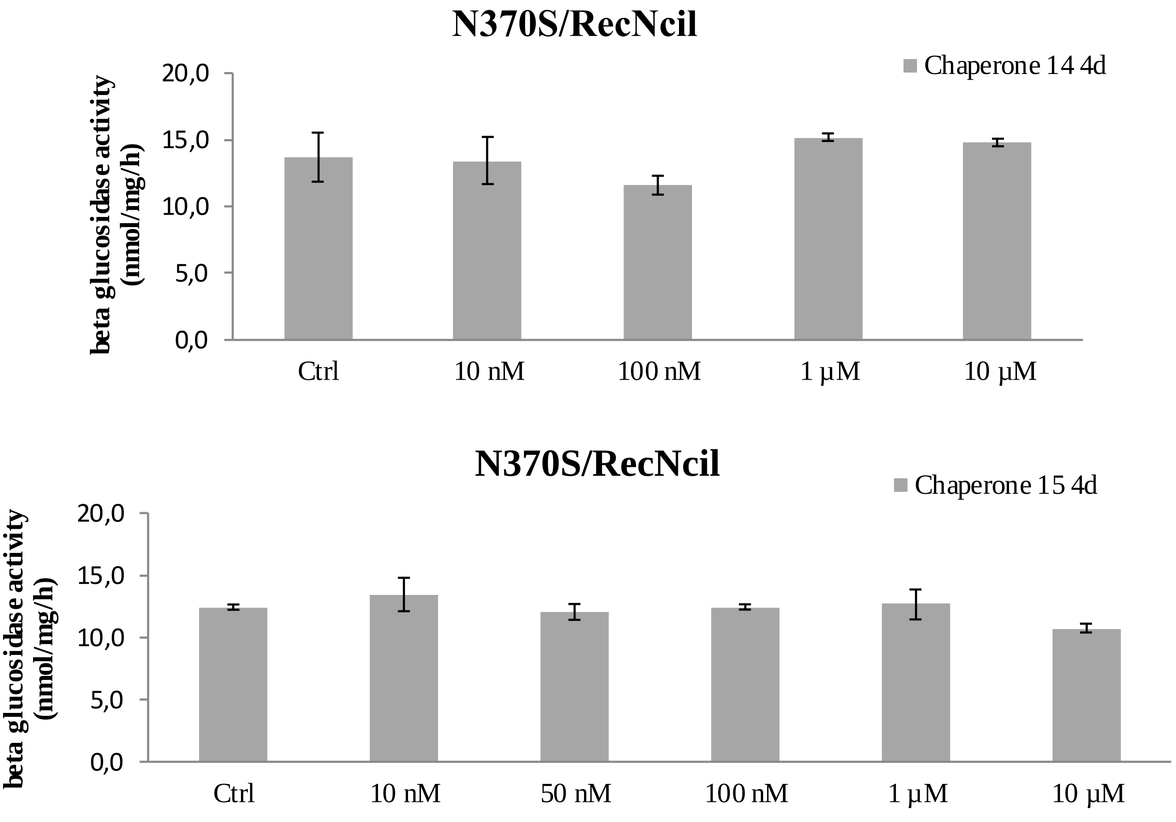

Figure S50. Fibroblasts derived from GD patients bearing N370S/RecNcil mutations were incubated without (control, ctrl) or with 7 different concentrations (10 nM, 50 nM, 100 nM, 1 µM, 10 µM, 50 µM, 100 µM) of compounds **14** and **15**. After 4 days, the flasks containing cells incubated with 50 µM and 100 µM concentrations of **14** or **15** showed low cell viability that hampered to proceed with the assay. For the other concentrations, the GCase activity was determined in lysates from treated fibroblasts. Reported data are mean S.D. (n=2).

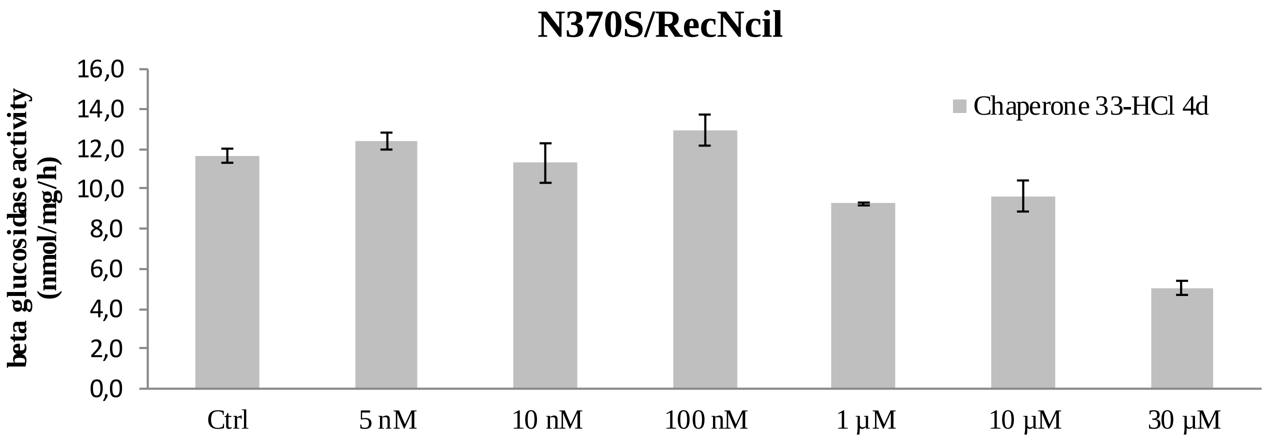

Figure S51. Fibroblasts derived from GD patients bearing N370S/RecNcil mutations were incubated without (control, ctrl) or with 7 different concentrations (5 nM, 10 nM, 100 nM, 1 µM, 10 µM, 30 µM) of compound **33·HCl**. After 4 days, the GCase activity was determined in lysates from treated fibroblasts. Reported data are mean S.D. (n=2).

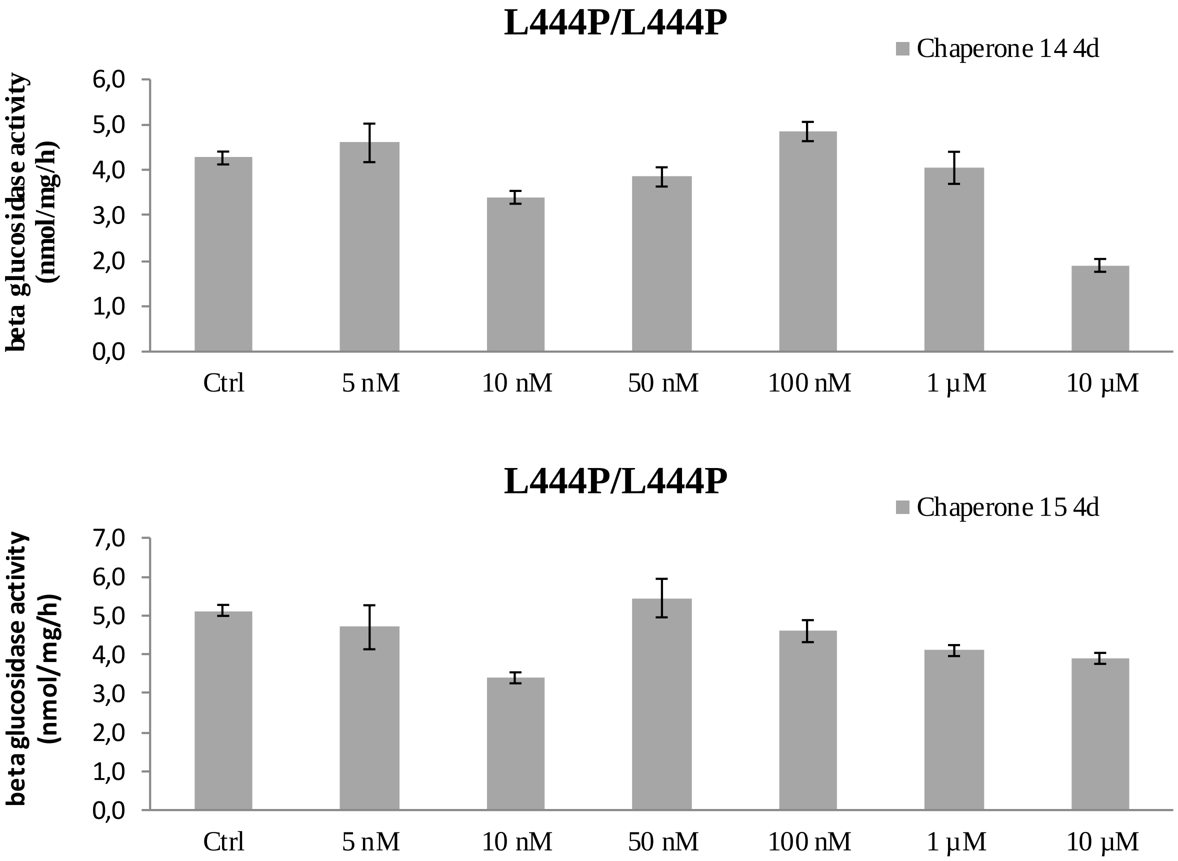

Figure S52. Fibroblasts derived from GD patients bearing L444P/L444P mutations were incubated without (control, ctrl) or with 7 different concentrations (5 nM, 10 nM, 50 nM, 100 nM, 1 µM, 10 µM, 50 µM) of compounds **14** or **15**. After 4 days, the flasks containing cells incubated with 50 µM concentrations of **14** or **15** showed low cell viability that hampered to proceed with the assay. For the other concentrations, the GCase activity was determined in lysates from treated fibroblasts. Reported data are mean S.D. (n=2).

Molecular Docking

Molecular docking was carried out with acid-beta-glucosidase (PDB ID 2NSX)[1] using Maestro and GLIDE software as implemented in Schrödinger suite.[2] The D chain was selected. Following the classical protocol, water molecules, sulfate ions, *N*-acetylglucosamine and the chaperone 5-hydroxymethyl-3,4-dihydroxypiperidine (Isofagomine, IFG) were removed from the model. The protein was preprocessed by assigning bond orders, adding hydrogens, converting selenomethionine into methionine, creating disulfide bonds and adding cap termini. The orientations of the hydroxyl groups from the Ser, Thr and Tyr, the sulfhydryl protons of Cys and methyl protons of Met were optimized. The positions of the hydrogen atoms on the histidine, asparagine and glutamine residues were assigned to ensure the correct ionization states using Epik (pH 7.0 ±2.0). Then, the protein was optimized with OPLS2005e force field. Grid was prepared with a box size of 25x25x25Å, centred on the ligand at the active site, using OPLS2005 force field. Glide was run on XP mode (Extra Precision) using as input the structures restraining nitrogen inversion and conformations to keep the chair orientation. Schrödinger Suite 2018-1 was used for all computational calculations. The binding models for all the compounds were constructed using reference ligand in the binding site. We considered compounds **14** and **15** protonated at the nitrogen atom so, two diastereoisomers were evaluated for each compound. Conformational preparation of ligands started from two different chairs in each case so, a total of eight docked structures were evaluated (Figure S53). In all cases, values of GLIDE score within the same range were obtained (Figures S54 and S55) with some preference for compound **15**, in particular for the $^{1}C_{4}$ chair. This preference is parallel to the conformational stability since the preferred pose corresponds to the structures with the two aliphatic chains at C-2 in an equatorial orientation and only one hydroxyl group in an axial orientation. However, in those cases corresponding to the best values of docking score, the piperidine ring is oriented opposite as it is for IFG. Indeed, MMP(G)BSA binding energy data obtained from more reliable MD studies lead to a different result (see below), evidencing that preliminary results derived from molecular docking should be managed with caution. 5-Hydroxymethyl-3,4-dihydroxipiperidine (IFG), the ligand reported as a chaperone initially present in the studied structure,[1] showed a higher GLIDE score. This ligand is completely inserted in the binding site (Figure S56) whereas in the case of **14** and **15** the hydrophobic chains are mostly oriented towards the external part of the site. Only, the best pose, found for (*S*)-**15H**$^{+}$ $^{1}C_{4}$, maintains the aliphatic chains inside the binding site (Figure S55), thus justifying the higher GLIDE score obtained.

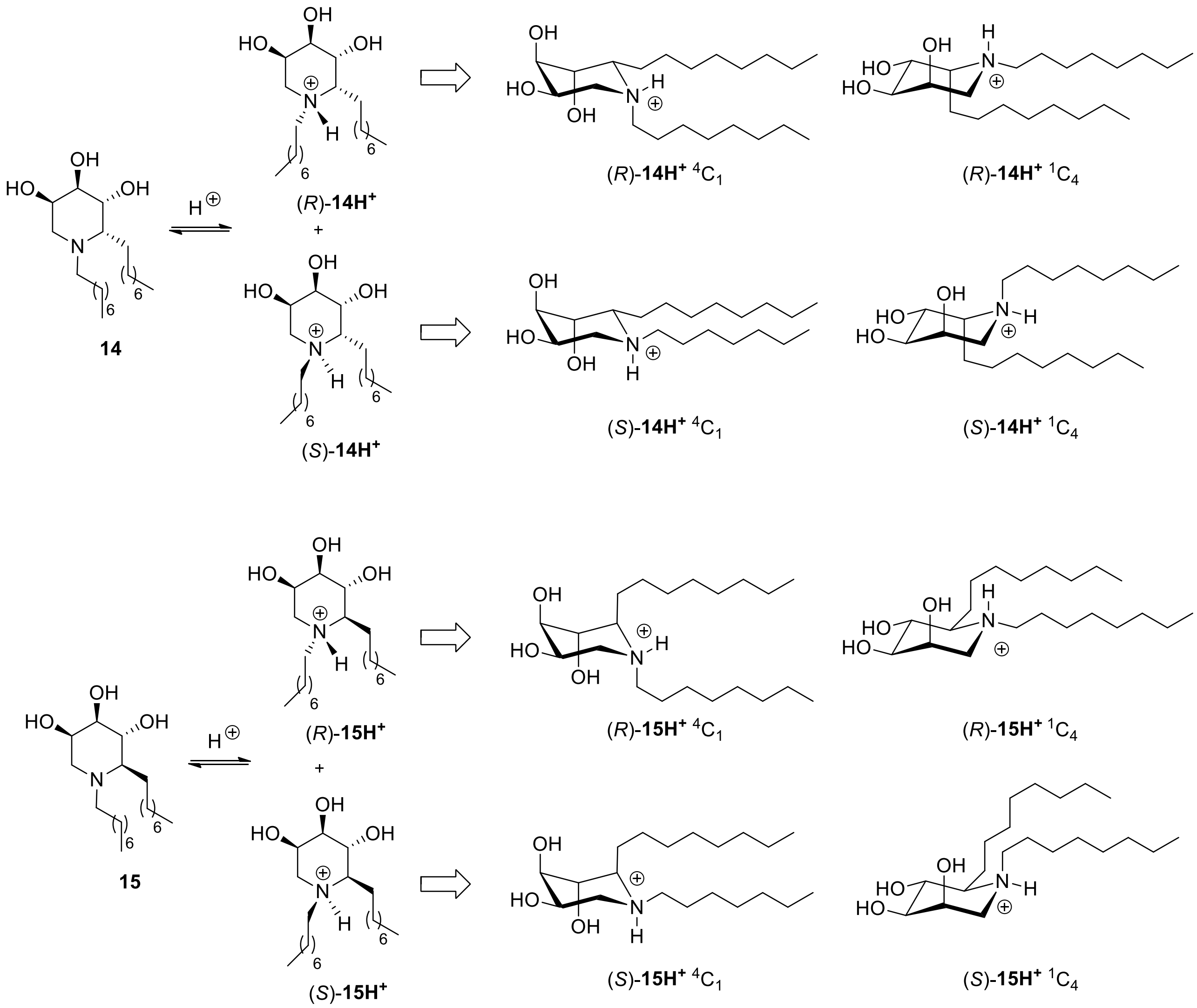

Figure S53. Starting docked structures into GCase from PDB ID: 2NSX

All the poses show interactions with the main residues of the binding site through the hydroxyl groups, although only (*R*)-**15H**$^+$ $^1C_4$ presents interactions with the three hydroxyl groups in a similar way to 5-hydroxymethyl-3,4-dihydroxypiperidine (IFG), i.e.: with Asn396 and Asp127.

However, this pose presents the aliphatic chains exposed to the solvent. In general, all poses present parts of the aliphatic chains exposed to the solvent, with the exception of (*S*)-**15H**$^+$ $^1C_4$ in which only a terminal ethyl group is exposed. The rest of the aliphatic chains interact with the binding site.

In general, $^1C_4$ conformation is preferred and the *S* isomer showed the best GLIDE score.

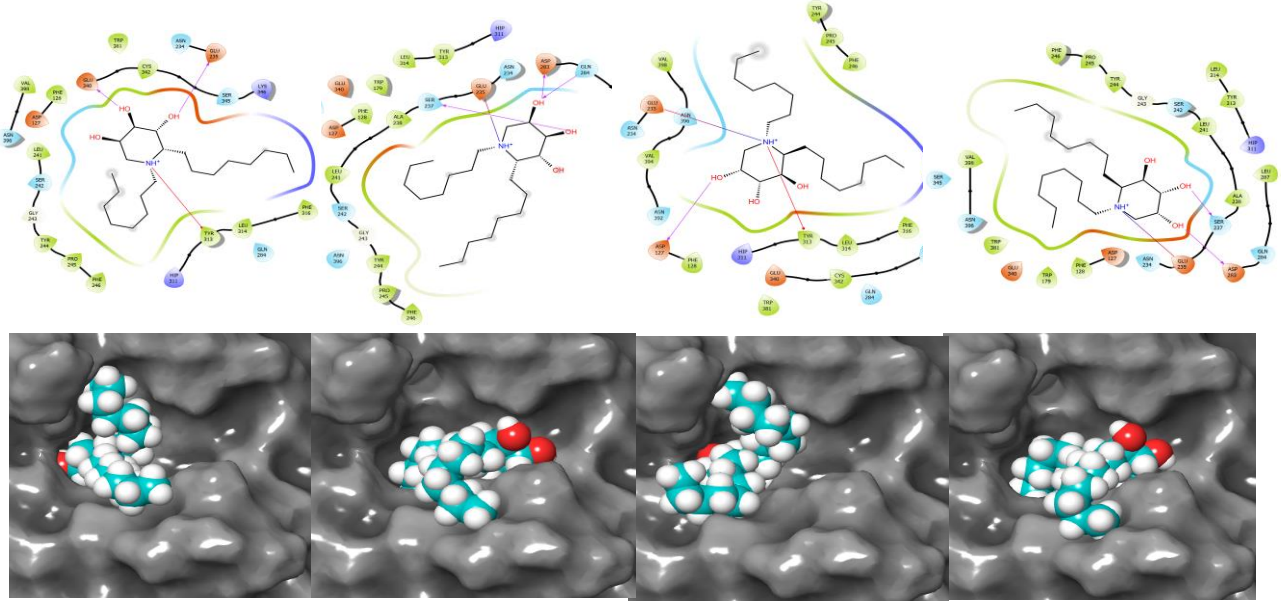

(R)-**14H**$^{+}$ $^{4}C_{1}$ (-4.331) | (R)-**14H**$^{+}$ $^{1}C_{4}$ (-6.231) | (S)-**14H**$^{+}$ $^{4}C_{1}$ (-5.144) | (S)-**14H**$^{+}$ $^{1}C_{4}$ (-6.071)

Figure S54. Best poses obtained after docking for **14H**$^{+}$. All views are in the same orientation. Top: 2D map of interactions. Bottom: The ligand is shown at the binding site as CPK model. GLIDE score is given between brackets.

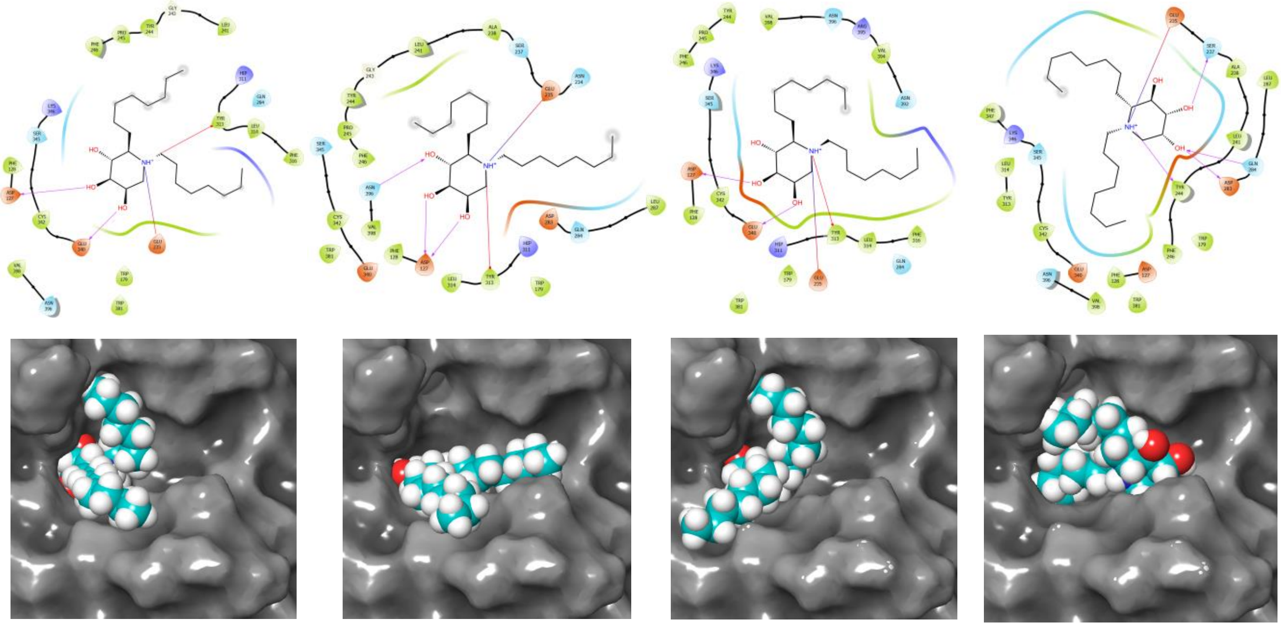

(R)-**15H**$^{+}$ $^{4}C_{1}$ (-5.680) | (R)-**15H**$^{+}$ $^{1}C_{4}$ (-6.354) | (S)-**15H**$^{+}$ $^{4}C_{1}$ (-6.260) | (S)-**15H**$^{+}$ $^{1}C_{4}$ (-6.614)

Figure S55. Best poses obtained after docking for **15H**$^{+}$. All views are in the same orientation. Top: 2D map of interactions. Bottom: The ligand is shown at the binding site as CPK model. GLIDE score is given between brackets.

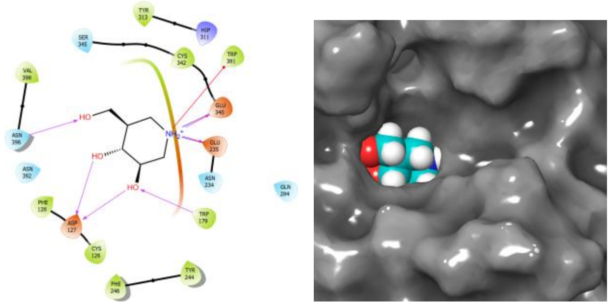

5-hydroxymethyl-3,4-dihydroxypiperidine
(-8.654)

Figure S56. Best pose obtained after docking for the ligand 5-hydroymethyl-3,4-dihydroxypiperidine (IFG). All views are in the same orientation. Top: 2D map of interactions. Bottom: The ligand is shown at the binding site as CPK model. GLIDE score is given between brackets.

Molecular Dynamics (MD) simulations

MD simulations were carried out with AMBER18 suite of programs.[3] Parameters for ligands (*R*)-**14H**$^+$, (*S*)-**14H**$^+$, (*R*)-**15H**$^+$ and (*S*)-**15H**$^+$ were generated with the antechamber module using the general Amber force field (GAFF2),[4] with partial charges calculated using AM1-BCC method. The ff14SB force field[5] was employed to properly model the protein. The protein, together with the corresponding ligand, was neutralized and immersed in a water box with a 10 Å buffer of TIP3P[6] water molecules. A two-stage geometry optimization approach was carried out: i) minimization of only the positions of solvent molecules executed by 500 cycles of steepest descent minimization followed by 500 cycles of conjugate gradient minimization and (ii) unrestrained minimization of all the atoms in the simulation cell executed by 2500 cycles of steepest descent minimization followed by 2500 cycles of conjugate gradient minimization. After system optimization, running of MD simulations was started on the systems by gradually heating each system in the NVT ensemble from 0 to 300 K for 100 ps using a Langevin thermostat with a coupling coefficient of 1.0/ps. Harmonic restraints of 10 kcal·mol-1 were applied to the solute, and the Langevin temperature coupling scheme[7] was used to control and equalize the temperature. The time step was kept at 2 fs during the heating stages, allowing potential inhomogeneity to self-adjust. Water molecules are treated with the SHAKE algorithm such that the angle between the hydrogen atoms is kept fixed. Long-range electrostatic effects are modelled using the particle-mesh-Ewald method.[8] Then 100 ps of density equilibration with a force constant of 2.0 kcal/mol·Å^2 on the complex was performed by releasing all the restraints. Finally, production trajectories were then run for 250 ns under the same simulation conditions. All MD simulations were replicated four times to ensure feasibility. MM/PBSA and MM/GBSA calculations using the procedure implemented in AMBER18 were carried out to estimate ligand-binding affinities.[9] The structures used in MM/P(G)BSA calculations were taken from the production runs. The average 500 snapshots were extracted from the whole MD trajectory at 500 ps intervals. For each snapshot, binding free energy was calculated as the difference between the free energy of the complex and the total of the free energies of the protein and the ligand. The cluster analysis of protein conformations was carried out using cpptraj module with average linkage as the clustering algorithm, and backbone atom RMSD as the distance metric. Pymol 2.0[10] was used for structural alignments and visualizations. For plotting graphs, MS Excel (2019) and OriginPro (2018) were used.

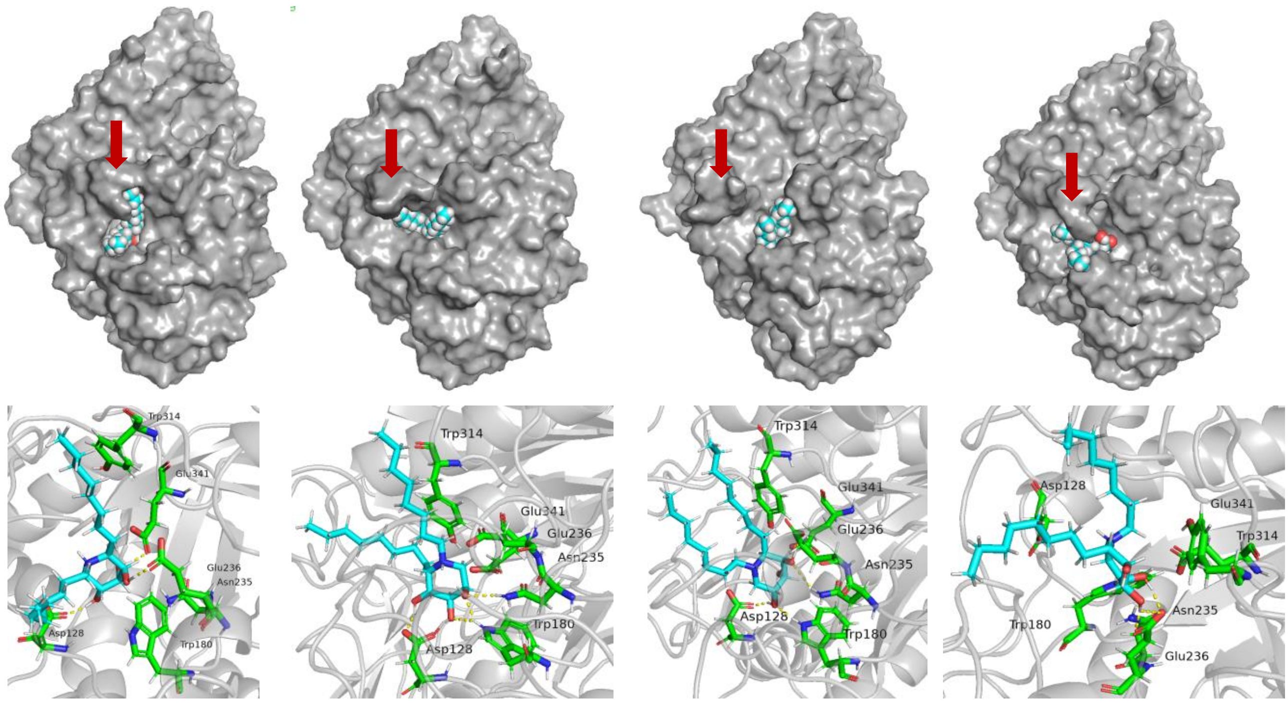

(*R*)-**14H**$^{+}$ $^{4}C_{1}$ (*R*)-**14H**$^{+}$ $^{1}C_{4}$ (*S*)-**14H**$^{+}$ $^{4}C_{1}$ (*S*)-**14H**$^{+}$ $^{1}C_{4}$

Figure S57. Final snapshots for MD unrestrained simulations of compound **14**. Top: Surface representation of the complex. The ligand (compound **14**) is shown as CPK with the C atoms in cyan. Bottom: Close-up view of GCase in complex with compound **14.** The ligand is colored in cyan. Residues are colored in green. Dashed yellow lines indicate H-bond interactions. All MD simulations were made by quadruplicate and the RMSD verified. The red arrow indicates the flexible loop formed by residues 345-349.

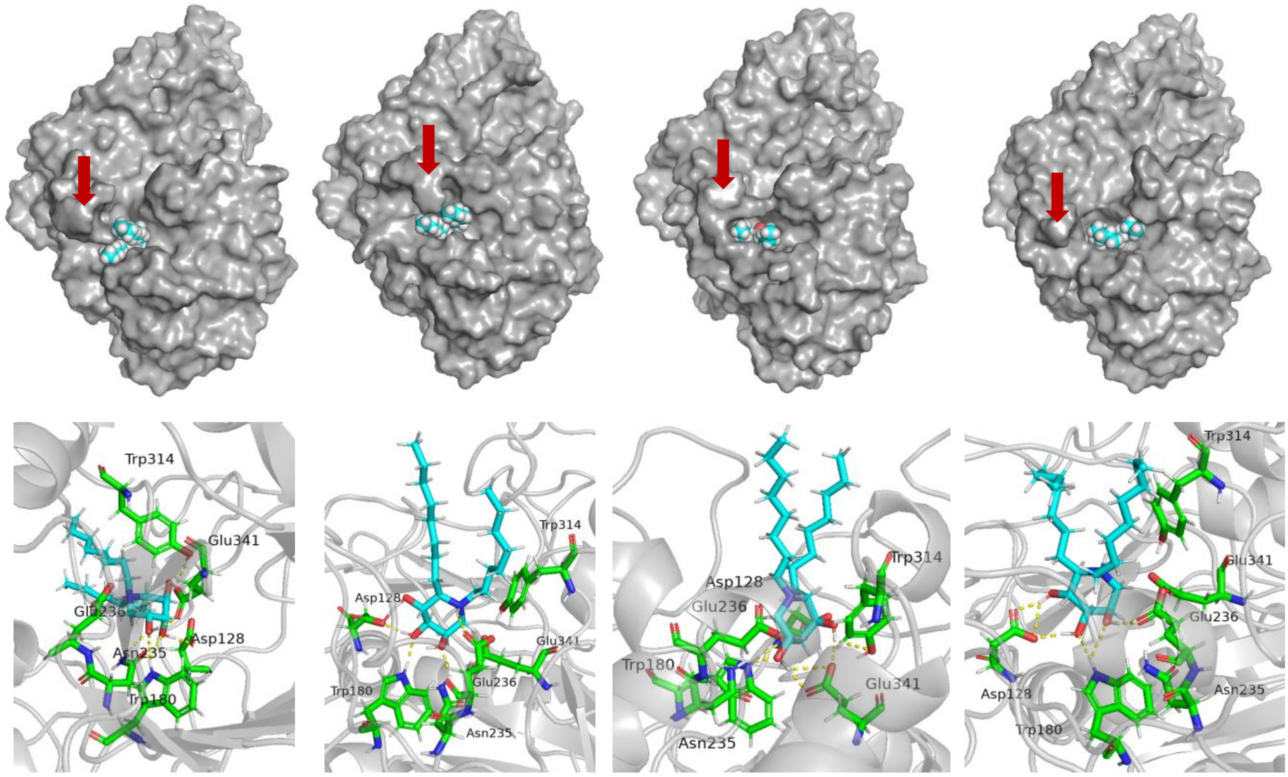

(*R*)-**15H** $^{++}$ $^{4}C_{1}$ (*R*)-**15H**$^{+}$ $^{1}C_{4}$ (*S*)-**15H**$^{+}$ $^{4}C_{1}$ (*S*)-**15H**$^{+}$ $^{1}C_{4}$

Figure S58. Final snapshots for MD unrestrained simulations of compound **15**. Top: Surface representation of the complex. The ligand (compound **15**) is shown as CPK with the C atoms in cyan. Bottom: Close-up view of GCase in complex with compound **15.** The ligand is colored in cyan. Residues are colored in green. Dashed yellow lines indicate H-bond interactions. All MD simulations were made by quadruplicate and the RMSD verified. The red arrow indicates the flexible loop formed by residues 345-349.

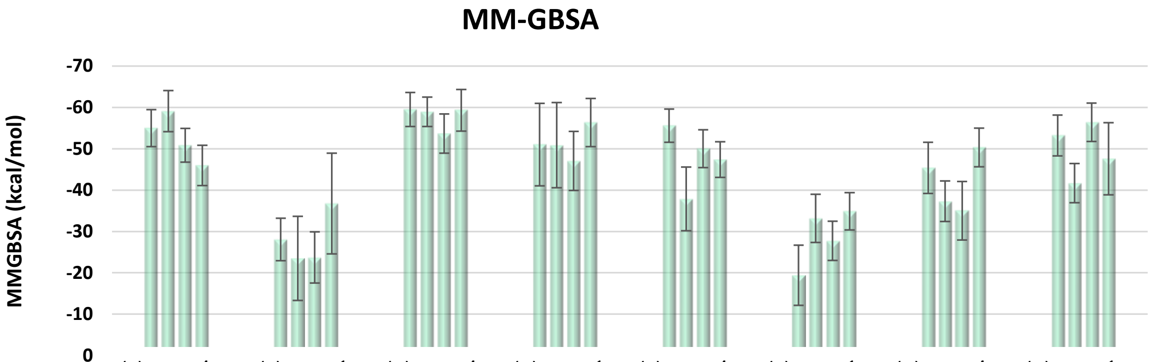

	(S)-**15H**+ $^4C_1$	(S)-**15H**+ $^1C_4$	(*R*)-**15H**+ $^4C_1$	(*R*)-**15H**+ $^1C_4$	(S)-**14H**+ $^4C_1$	(S)-**14H**+ $^1C_4$	(*R*)-**14H**+ $^4C_1$	(*R*)-**14H**+ $^1C_4$
replica 1	**-54.9831**	**-28.0447**	**-59.4868**	**-51.0149**	**-55.5775**	**-19.4003**	**-45.377**	**-53.2244**
replica2	**-59.1039**	**-23.495**	**-58.9253**	**-50.861**	**-37.8707**	**-33.1669**	**-37.3098**	**-41.7188**
replica 3	**-50.8435**	**-23.7052**	**-53.6712**	**-47.0525**	**-50.0465**	**-27.7487**	**-35.0347**	**-56.402**
replica 4	**-45.9907**	**-36.7638**	**-59.3089**	**-56.3415**	**-47.3841**	**-34.9025**	**-50.3419**	**-47.5852**

Figure S59. MMGBSA calculation for compounds **14** and **15**.

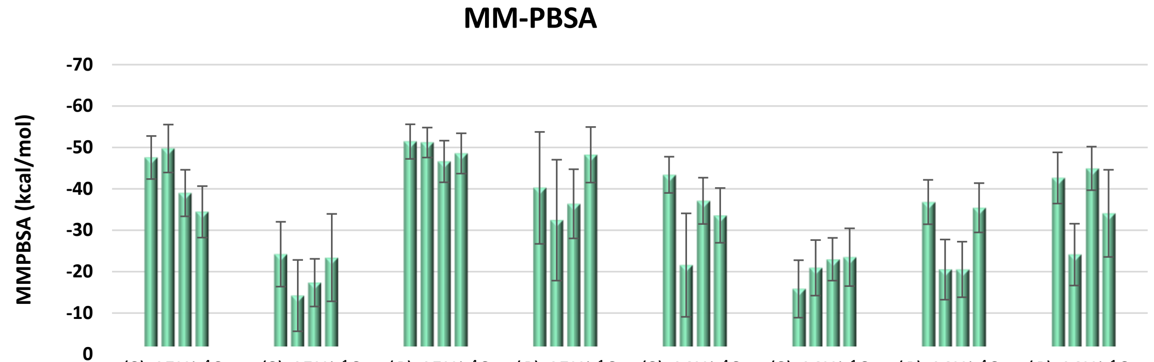

	(S)-**15H**+ $^4C_1$	(S)-**15H**+ $^1C_4$	(*R*)-**15H**+ $^4C_1$	(*R*)-**15H**+ $^1C_4$	(S)-**14H**+ $^4C_1$	(S)-**14H**+ $^1C_4$	(*R*)-**14H**+ $^4C_1$	(*R*)-**14H**+ $^1C_4$
replica 1	**-47.5948**	**-24.1799**	**-51.4538**	**-40.2421**	**-43.3862**	**-15.8083**	**-36.8053**	**-42.6413**
replica2	**-49.7441**	**-14.2029**	**-51.2257**	**-32.447**	**-21.5437**	**-20.9305**	**-20.4927**	**-24.1226**
replica 3	**-39.0043**	**-17.3354**	**-46.629**	**-36.3711**	**-37.1021**	**-22.9755**	**-20.4927**	**-44.9528**
replica 4	**-34.4508**	**-23.3625**	**-48.5604**	**-48.2287**	**-33.5799**	**-23.472**	**-35.413**	**-34.0754**

Figure S60. MMPBSA calculation for compounds **14** and **15**.

References

[1] Lieberman, R.L.; Wustman, B.A.; Huertas, P.; Powe, A.C.; Pine, C.W.; Khanna, R.; Schlossmacher, M.G.; Ringe, D.; Petsko, G.A. Structure of acid β-glucosidase with pharmacological chaperone provides insight into Gaucher disease. *Nat. Chem. Biol.* **2007**, *3*, 101-107.
[2] Schrödinger Release 2020-1: Maestro, Shcrödinger, LLC, New York, NY, 2020.
[3] Case, D. A.; Ben-Shalom, I. Y.; Brozell, S. R.; Cerutti, D. S.; III, T. E. C.; Cruzeiro, V. W. D.; Darden, T. A.; Duke, R. E.; Ghoreishi, D.; Gilson, M. K.; Gohlke, H.; Goetz, A. W.; Greene, D.; Harris, R.; Homeyer, N.; Huang, Y.; Izadi, S.; Kovalenko, A.; Kurtzman, T.; Lee, T. S.; LeGrand, S.; Li, P.; Lin, C.; Liu, J.; Luchko, T.; Luo, R.; Mermelstein, D. J.; Merz, K. M.; Miao, Y.; Monard, G.; Nguyen, C.; Nguyen, H.; Omelyan, I.; Onufriev, A.; Pan, F.; Qi, R.; Roe, D. R.; Roitberg, A.; Sagui, C.; Schott-Verdugo, S.; Shen, J.; Simmerling, C. L.; Smith, J.; Salomon-Ferrer, R.; Swails, J.; Walker, R. C.; Wang, J.; Wei, H.; Wolf, R. M.; Wu, X.; Xiao, L.; York, D. M.; Kollman, P. A., *AMBER 2018*, Reference Manual, *University of California, San Francisco* **2018**.
[4] Wang, J.; Wolf, R. M.; Caldwell, J. W.; Kollman, P. A.; Case, D. A. Development and testing of a general amber force field. *J. Comput. Chem.* **2004,** *25*, 1157-1174.
[5] Maier, J. A.; Martinez, C.; Kasavajhala, K.; Wickstrom, L.; Hauser, K. E.; Simmerling, C. ff14SB: Improving the Accuracy of Protein Side Chain and Backbone Parameters from ff99SB. *J. Chem. Theory Comput.* **2015,** *11*, 3696-3713.
[6] Jorgensen, W. L.; Chandrasekhar, J.; Madura, J. D.; Impey, R. W.; Klein, M. L. Comparison of simple potential functions for simulating liquid water. *J. Chem. Phys.* **1983,** *79*, 926-935.
[7] Matubayasi, N.; Nakahara, M. Reversible molecular dynamics for rigid bodies and hybrid Monte Carlo. *J. Chem. Phys.* **1999,** *110*, 3291-3301.
[8] Darden, T.; York, D.; Pedersen, L. Particle mesh Ewald: An N·log(N) method for Ewald sums in large systems. *J. Chem. Phys.* **1993,** *98*, 10089-10092.
[9](a) Wang, E.; Sun, H.; Wang, J.; Wang, Z.; Liu, H.; Zhang, J. Z. H.; Hou, T. End-Point Binding Free Energy Calculation with MM/PBSA and MM/GBSA: Strategies and Applications in Drug Design. Chem. Rev. **2019**, *119*, 9478-9508. (b) Genheden, S.; Ryde, U. The MM/PBSA and MM/GBSA methods to estimate ligand-binding affinities. *Expert Opin. Drug Discovery* **2015,** *10,* 449-461.
[10] The PyMOL Molecular Graphics System, Version 2.0 Schrödinger, LLC.
